# Loss of fragile site-associated tumor suppressor promotes antitumor immunity via macrophage polarization

**DOI:** 10.1038/s41467-021-24610-x

**Published:** 2021-07-14

**Authors:** Lijuan Zhang, Kai Zhang, Jieyou Zhang, Jinrong Zhu, Qing Xi, Huafeng Wang, Zimu Zhang, Yingnan Cheng, Guangze Yang, Hongkun Liu, Xiangdong Guo, Dongmei Zhou, Zhenyi Xue, Yan Li, Qi Zhang, Yurong Da, Li Liu, Zhinan Yin, Zhi Yao, Rongxin Zhang

**Affiliations:** 1 Key Laboratory of Immune Microenvironment and Diseases (Ministry of Education), Department of Immunology, School of Basic Medical Sciences, Tianjin Medical University, Tianjin, China; 2 Guangdong Province Key Laboratory for Biotechnology Drug Candidates, School of Life Sciences and Biopharmaceutics, Guangdong Pharmaceutical University, Guangzhou, China; 3 School of Life Science, Shanxi Normal University, Linfen, China; 4 Institute of Integrative Medicines for Acute Abdominal Diseases, Nankai Hospital, Tianjin, China; 5 Department of Radiology, The University of Texas Southwestern Medical Center, Dallas, TX USA; 6 The First Affiliated Hospital, Biomedical Translation Research Institute and Guangdong Province Key Laboratory of Molecular Immunology and Antibody Engineering, Jinan University, Guangzhou, China; 7 Key Laboratory of Immune Microenvironment and Diseases (Ministry of Education), Tianjin Medical University, Tianjin, China

**Keywords:** Cancer immunotherapy, Tumour-suppressor proteins, Monocytes and macrophages, Tumour immunology

## Abstract

Common fragile sites (CFSs) are specific breakage-prone genomic regions and are present frequently in cancer cells. The (E2-independent) E3 ubiquitin-conjugating enzyme FATS (fragile site-associated tumor suppressor) has antitumor activity in cancer cells, but the function of FATS in immune cells is unknown. Here, we report a function of FATS in tumor development via regulation of tumor immunity. *Fats*^*−/−*^ mice show reduced subcutaneous B16 melanoma and H7 pancreatic tumor growth compared with WT controls. The reduced tumor growth in *Fats*^*−/−*^ mice is macrophage dependent and is associated with a phenotypic shift of macrophages within the tumor from tumor-promoting M2-like to antitumor M1-like macrophages. In addition, FATS deficiency promotes M1 polarization by stimulating and prolonging NF-κB activation by disrupting NF-κB/IκBα negative feedback loops and indirectly enhances both CD4^+^ T helper type 1 (Th1) and cytotoxic T lymphocyte (CTL) adaptive immune responses to promote tumor regression. Notably, transfer of *Fats*^*−/−*^ macrophages protects mice against B16 melanoma. Together, these data suggest that FATS functions as an immune regulator and is a potential target in cancer immunotherapy.

## Abstract (web summary)

The role of common fragile site associated genes such as *FATS* in the immune system is unclear. Here the authors show that deletion of *Fats* in a mouse tumour model leads to reduced tumour growth and change of macrophage phenotype from an M2-like to M1-like function.

## Introduction

The tumor microenvironment (TME) contains innate and adaptive immune cells that recognize and destroy tumors^1^. However, tumors not only manage to escape the host immune system but also benefit from infiltrating immune cells by modifying the functions of these cells to establish an immunosuppressive microenvironment that is favorable for tumor progression^2^. During all stages of tumor development, tumor-associated macrophages (TAMs) are present in the TME and play pivotal roles in both antitumor and protumor effects depending on their classically activated M1 or alternatively activated M2 subtype, respectively^3^. Monocyte-derived macrophages polarize into either the M1 or the M2 subtype depending on environmental cues^4^. In general, M1 macrophages are potent antitumor cells that highly express MHCII and inducible nitric oxide synthase (NOS2), are efficient producers of inflammatory cytokines (interleukin-1β (IL-1β), IL-6, IL-12), and participate as inducer and effector cells in polarized Th1 responses^4^. However, M2 macrophages display protumor functions. Circulating monocytes are recruited by tumor cells and adopt features common to M2-like macrophages^5^. M2-like TAMs play a key role in tumor growth and progression by producing molecules to promote angiogenesis as well as the survival and metastasis of tumor cells^6,7^. Moreover, TAMs suppress effective antitumor immune responses by expressing immunosuppressive molecules (IL-10, transforming growth factor-β (TGF-β))^8,9^. Either ablation of TAMs^10,11^ or switching of macrophages into the antitumor M1-like phenotype^12,13^ results in a significant reduction in tumor growth. Based on current studies^14–16^, an immunotherapeutic strategy targeting M1/M2 macrophage polarization in the TME may be effective and promising.

As an inherent component of chromosomal structure, common fragile sites (CFSs) are evolutionarily conserved and breakage-prone genomic regions that exist in all individuals^17^. CFSs preferentially form gaps or breaks on metaphase chromosomes upon replication stress and are frequently found in cancer cells^18^. Current studies show that instability-induced alterations at fragile sites contribute to tumorigenesis, considering that genomic instability is a hallmark of cancer^19^. However, studies on fragile site functions in immunity are limited, such that GO analysis showed that the terms related to immune response were significantly overrepresented in genes located at fragile sites^20^. Functional characterization of fragile site-connected components indicated that a number of immune-related genes are associated with fragile sites, for example, LIF, which can induce macrophage differentiation^20^. However, limited experimental data have shown the link between CFSs and immune function to date. C10orf90 (also called D7Ertd443e in mice) is a fragile-site-associated gene that maps to the common fragile site FRA10F on chromosome 10q26 in the human genome^21^. Previous studies identified it as fragile site-associated tumor suppressor (FATS) because the gene was susceptible to breakage in an irradiation (IR)-induced mouse tumor model^21^. However, current studies on FATS have mainly focused on evaluating its mutation and potential functions in cancer cells, including non-small-cell lung cancer (NSCLC)^22^, breast cancer^23^, and ovarian cancer cells^24^. There are few studies on FATS and its functional characterization in immune cells, which play crucial roles in tumor formation and growth, that have been well established to date.

In this work, we utilize *Fats*^*−/−*^ mice to explore the role of FATS in tumor immunity. FATS deficiency reduces tumor growth in vivo and increases the survival of mice. We demonstrate that FATS functions as a regulator of macrophage polarization both in vitro and in vivo. Furthermore, the polarization of macrophages toward an antitumor phenotype induced by FATS deficiency correlates with the enhanced antitumor T cell activity within the TME. Our results suggest that FATS functions as a molecular switch that regulates antitumor immunity. We also provide insights into the role of FATS in cancer development.

## Results

### FATS deficiency prevents tumor growth in vivo

**Fig. 1:**
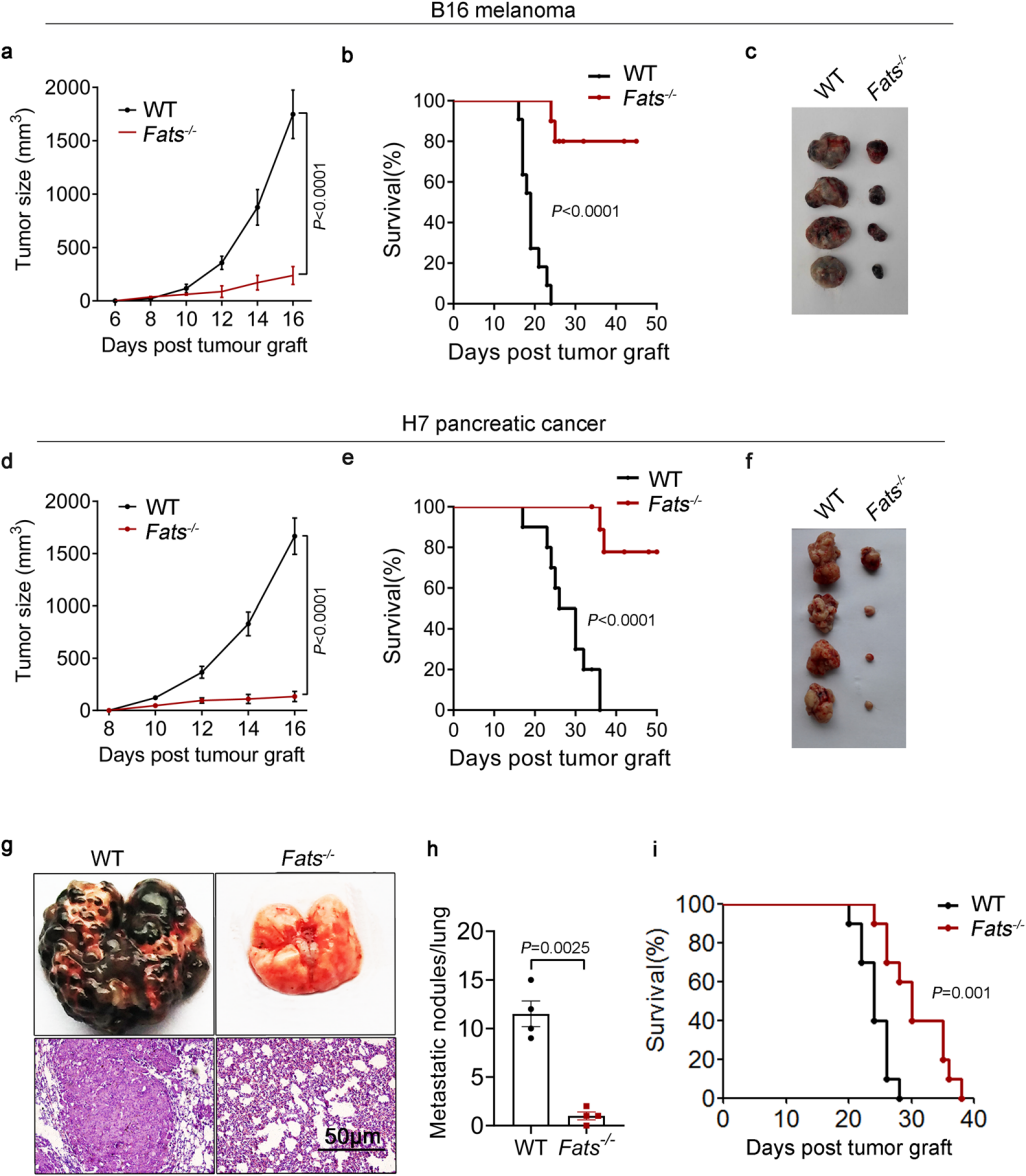
FATS deficiency prevents tumor growth in vivo. **a**–**c** Tumor volumes, survival data, and representative image of tumors in wild type (WT) and *Fats*^−/−^ mice after B16 melanoma cell inoculation (**a** *n* = 12 for WT, *n* = 10 for *Fats*^−/−^ mice from two independent experiments; **b** *n* = 11 for WT, *n* = 10 for *Fats*^−/−^ mice from two independent experiments). **d**–**f** Tumor volumes, survival data, and representative image of tumors in WT and *Fats*^−/−^ mice after H7 pancreatic cancer cell inoculation (**d** *n* = 7 mice per group; **e** *n* = 10 mice per group). **g**–**i** B16 melanoma cells were intravenously injected into WT or *Fats*^−/−^ mice to induce melanoma tumor formation and subsequent lung metastasis. On day 20, lungs were isolated to count tumors. **g** Representative images of hematoxylin and eosin-stained sections of lungs from B16 tumor-bearing WT and *Fats*^−/−^ mice on day 20. **h** Quantification of lung metastasis after B16 tumor cell challenge (*n* = 4 mice per group). **i** The survival of mice bearing B16 lung metastases (*n* = 10 mice per group from two independent experiments). Data are presented as mean ± s.e.m. in **a**, **d**, **h**; two-way ANOVA in **a**, **d**; two-tailed unpaired Student’s *t*-test in **h**; log-rank test in **b**, **e**, **i**. Source data are provided as a Source Data file.

To investigate whether FATS plays an important role in tumor growth, B16 melanoma cells were injected subcutaneously into *Fats*^−/−^ or WT mice. Compared to WT mice, *Fats*^−/−^ mice exhibited reduced tumor growth and prolonged survival (Fig. 1a–c). Similar results were observed in *Fats*^−/−^ mice injected subcutaneously with H7 pancreatic cancer cells (Fig. 1d–f). Moreover, we observed a significant reduction in lung metastasis of B16 tumors after induction of FATS deficiency, and *Fats*^−/−^ mice exhibited prolonged survival in the metastasis model (Fig. 1g–i). Collectively, these data demonstrate that FATS deficiency impairs tumor growth in vivo and prolongs animal survival.

### FATS deficiency inhibits M2-like TAM function within tumors

**Fig. 2:**
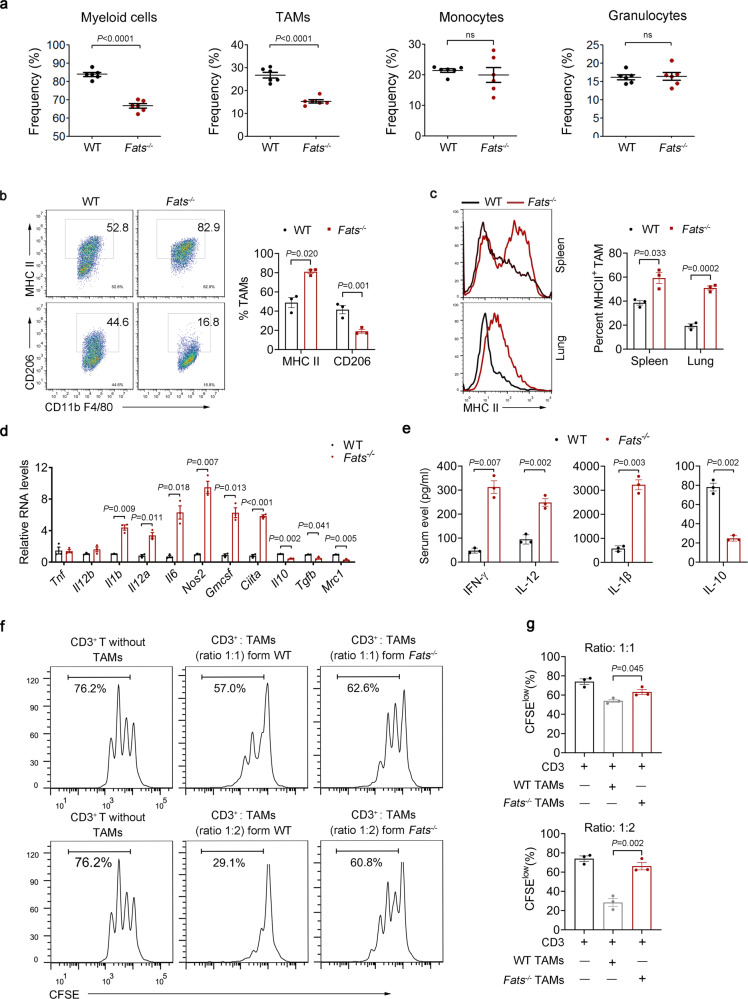
FATS deficiency inhibits M2-like TAMs within tumors. **a** Flow cytometric quantification of the total myeloid cell, TAM, monocyte, and granulocyte populations in B16 tumor-bearing WT and *Fats*^−/−^ mice (*n* = 6 mice per group). **b** Flow cytometric analysis and quantification of MHCII and CD206 expression in the CD11b^+^F4/80^+^ (TAM) cell population in B16 tumors on day 18 post implantation (*n* = 3 mice per group). **c** Flow cytometric analysis and quantification of MHCII expression in spleen and alveolar macrophages in WT and *Fats*^−/−^ mice with B16 metastases (*n* = 3 mice per group). **d** mRNA expression of selected M1 and M2 markers in TAMs isolated from B16 tumors as determined by Quantitative Real-time PCR (*n* = 3 biologically independent samples); data are presented relative to GAPDH expression levels and normalized to the mean expression levels in WT mice. **e** Serum levels of IFN-γ, IL-12, IL-1β, and IL-10 in B16 tumor-bearing mice were assessed using ELISA (*n* = 3 mice per group)**. f**, **g** In vitro suppressive activity of TAMs purified from B16 tumors on day 18 post implantation. **f** Representative histograms of CD3^+^ T cell proliferation at the corresponding CD3^+^ T to TAM ratio and **g** quantification of CD3^+^ T cell proliferation using carboxyfluorescein succinimidyl ester (CFSE) dilution (*n* = 3 biological replicates). Data are presented as mean ± s.e.m. in **a**–**e, g**; two-tailed unpaired Student’s *t*-test in **a**–**e** and **g**. ns not significant. Source data are provided as a Source Data file.

Studies over the past two decades have revealed that the TME is a determinant of tumor progression equally important to the tumor itself^2^. Bioinformatic analysis revealed that CFSs are associated with immune regulation^20^. Thus, we speculated that the delayed tumor growth in *Fats*^−/−^ mice might be associated with changes in the tumor immune microenvironment caused by FATS deficiency. As expected, quantification of tumor-infiltrating immune cells revealed that FATS deficiency significantly reduced the fractions of total myeloid cells (CD11b^+^) and TAMs (Ly6G^−^CD11b^+^F4/80^+^) but did not alter the fractions of monocytes (Ly6G-CD11b^+^Ly6C^hi^) and granulocytes (CD11b^+^Ly6G^+^) (Fig. 2a) as determined using the gating strategy shown in Supplementary Fig. 1a. Notably, FATS deficiency upregulated MHCII expression but decreased CD206 expression in TAMs (Fig. 2b), suggesting that FATS deficiency increases the M1-like macrophage fraction among TAMs. In addition, we analyzed macrophages in the spleens and lungs of mice with B16 metastases and found that *Fats*^−/−^ splenic and alveolar macrophages also displayed higher MHCII expression than control cells (Fig. 2c), suggesting M1 skewing. Furthermore, the mRNA expression of M1 and M2 markers was examined in TAMs isolated from *Fats*^−/−^ and WT B16 tumors. Higher expression of M1 markers (*Il12, Il1b, Il6*, and *Nos2*) was detected in *Fats*^−/−^ TAMs, whereas the expression of M2 markers (*Il10, Tgfb*, and *Mrc1*) was reduced (Fig. 2d). Similar results were observed in *Fats*^−/−^ tumor samples compared with WT tumors (Supplementary Fig. 2). Moreover, in B16 tumor-bearing *Fats*^−/−^ mice, serum levels of proinflammatory cytokines (IFN-γ, IL-12, and IL-1β) were increased, while the level of the anti-inflammatory cytokine IL-10 was significantly decreased (Fig. 2e). We subsequently tested the suppressive function of TAMs derived from B16 tumor-bearing *Fats*^−/−^ and WT mice on T cell proliferation, as a major pathogenic activity of TAMs is suppression of antitumor immune responses^8,25^. The proliferation of T cells was inhibited by B16 tumor-bearing WT TAMs compared with FATS-deficient TAMs or in the absence of TAMs, suggesting that the suppression of T cells by TAMs was relieved in B16 tumor-bearing *Fats*^−/−^ mice (Fig. 2f, g). Together, these results demonstrate that FATS deficiency significantly changes the M1/M2 ratio in TAMs, leading to a less immunosuppressive TME.

### Reduced tumor growth in ***Fats***^−^^/−^ mice is macrophage dependent

**Fig. 3:**
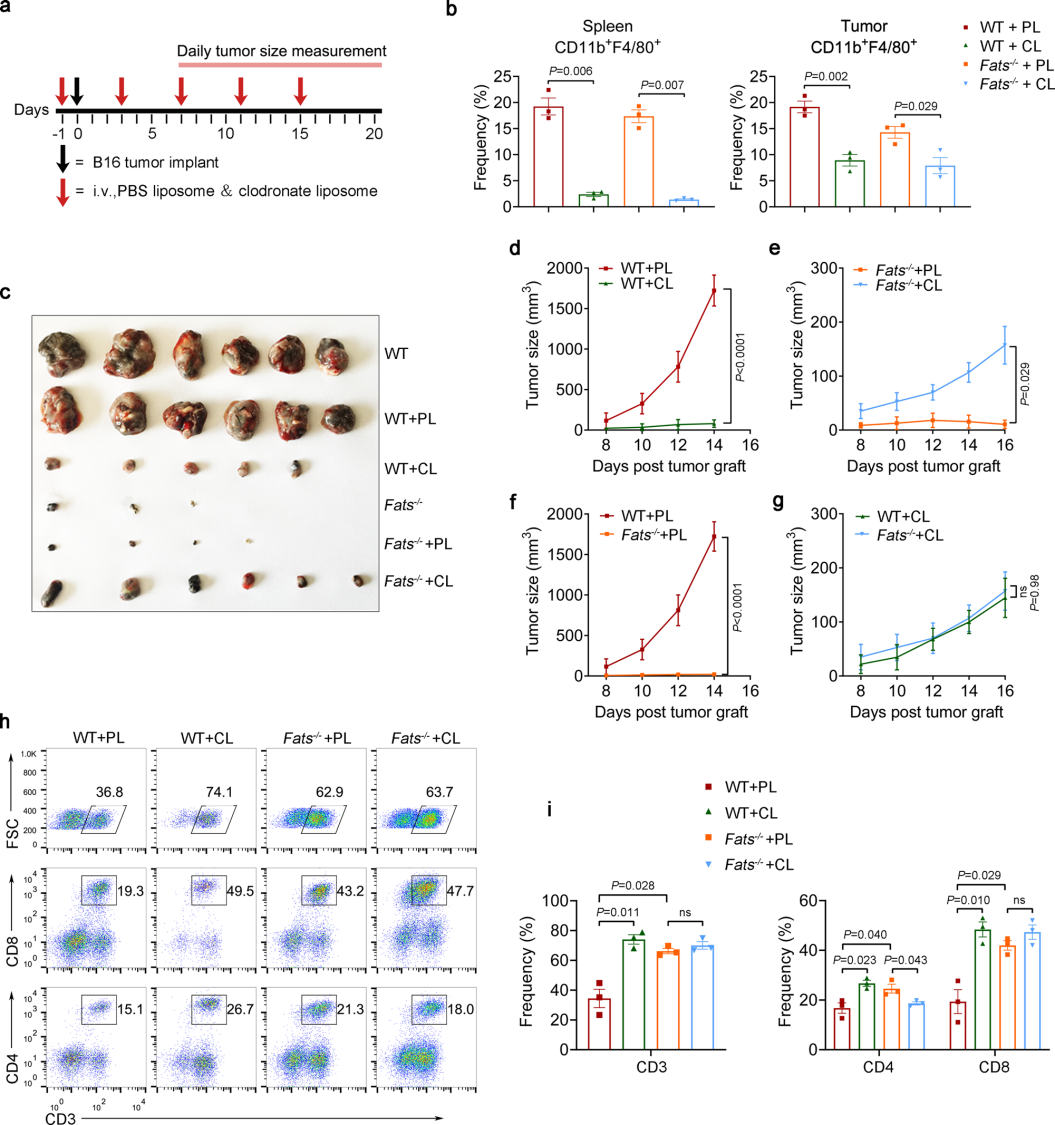
FATS deficiency inhibits B16 tumor growth via macrophages. **a** Schematic showing the in vivo depletion of macrophages. **b** Quantification of macrophages (CD11b^+^F4/80^+^) in spleens and tumors of WT and *Fats*^−/−^ mice from **a** on day 20 after PBS liposome (PL) or clodronate liposome (CL) treatment. Data are presented as mean ± s.e.m. (*n* = 3 mice per group; two-tailed unpaired Student’s *t*-test). **c** Representative image of tumors from WT and *Fats*^−/−^ mice from **a** with or without PL or CL treatment. (*n* = 6 mice per group, representative of two independent experiments). **d**–**g** Mean volumes of tumors derived from subcutaneously implanted B16 cells in WT and *Fats*^−/−^ mice from **a** after CL or PL treatment. Data are presented as mean ± s.e.m. (*n* = 6 mice per group; two-way ANOVA in **d**–**g**). **h** Flow cytometric analysis and **i** quantification of total CD3^+^ T, CD4^+^ T, and CD8^+^ T cells in tumors of mice from **a**. Data are presented as mean ± s.e.m. (*n* = 3 mice per group; two-tailed unpaired Student’s *t*-test). ns not significant. Source data are provided as a Source Data file.

To investigate whether FATS deficiency in macrophages is required for tumor repression, WT and *Fats*^−/−^ mice that had been prechallenged with B16 cells were serially treated with clodronate liposomes (Fig. 3a), which deplete macrophages from tissues^26^. The efficiency of macrophage depletion was confirmed by analyzing CD11b^+^F4/80^+^ cells in the spleen and tumors (Fig. 3b). In accordance with previous reports^10^, we observed that macrophage depletion inhibited B16 tumor growth in WT mice. In contrast, macrophage depletion in *Fats*^−/−^ mice did not inhibit tumor growth but instead promoted tumor growth to a certain extent (Fig. 3c–e). These results indicate that FATS deficiency reverses the function of macrophages in tumors, switching them from a protumor phenotype to an antitumor phenotype. When comparing tumor growth between the *Fats*^−/−^ and WT groups, both of which were treated with clodronate liposomes, we observed that FATS deficiency did not delay B16 tumor growth in the absence of macrophages (Fig. 3f–g), confirming that the reduced tumor growth in *Fats*^−/−^ mice is macrophage dependent. Notably, the number of B16 tumor-infiltrating T cells was substantially increased in WT mice but was not significantly changed in *Fats*^−/−^ mice after macrophage depletion (Fig. 3h, i), further indicating that FATS deficiency relieves immunosuppression in a macrophage-dependent manner.

### FATS blockade produces an intrinsic bias toward M1 polarization

**Fig. 4:**
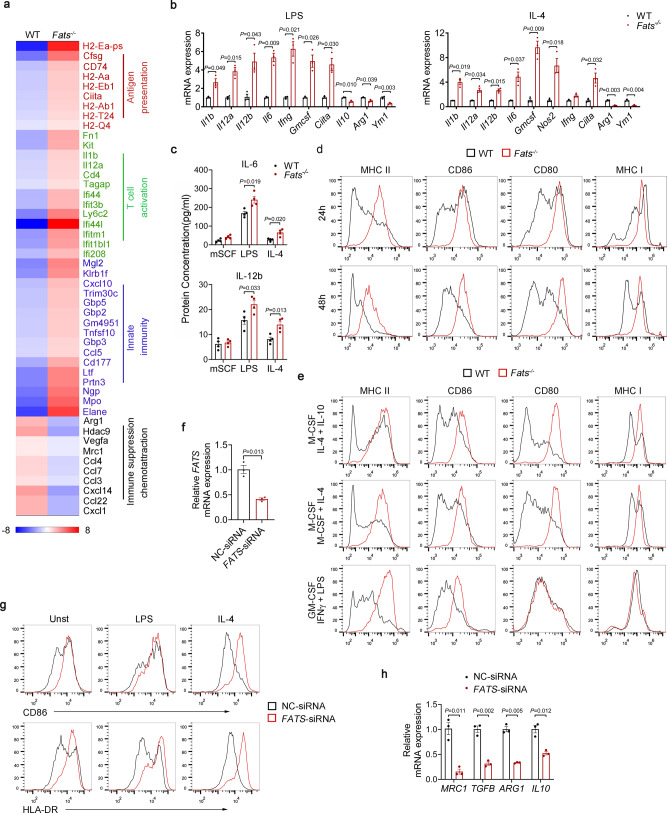
FATS blockade produces an intrinsic bias toward M1 polarization. **a** Heatmap of mRNA expression in WT and *Fats*^−/−^ BMDMs cultured under M-CSF differentiation conditions. **b** Relative expression levels of mRNAs related to the immune response in WT and *Fats*^−/−^ BMDMs after treatment with LPS for 6 h or IL-4 for 12 h by Quantitative Real-time PCR. (*n* = 3 biologically independent samples). **c** IL-6 and IL-12b concentrations in the supernatant of WT and *Fats*^−/−^ BMDMs stimulated with or without LPS or IL-4 (*n* = 4 biologically independent samples). **d** Flow cytometric analysis of MHCII, CD86, CD80, and MHCI expression in WT and *Fats*^−/−^ BMDMs cultured with B16 cell culture supernatant for 24 and 48 h. **e** Flow cytometric analysis of MHCII, CD86, CD80, and MHCI expression in WT and *Fats*^−/−^ BMDMs under TAM, M2, and M1 polarization conditions. **f** Human macrophages derived from CD14^+^ monocytes were treated with nontargeting control (NC) or *FATS* siRNA, and relative *FATS* expression was analyzed by Quantitative Real-time PCR (*n* = 3 biologically independent samples). **g** Flow cytometric analysis of HLA-DR and CD86 in human macrophages treated with NC siRNA or *FATS* siRNA and left unstimulated (Unst) or stimulated with LPS or IL-4 for 48 h. **h** The mRNA levels of *MRC1, TGFB, ARG1*, and *IL10* in human macrophages treated with NC siRNA or *FATS* siRNA and stimulated with IL-4 for 12 h (*n* = 3 biologically independent samples). Data are presented as mean ± s.e.m. in **b**, **c**, **f**, **h**. *P* values are calculated by two-tailed unpaired Student’s *t*-test in **b**, **c**, **f**, **h**; two independent experiments were carried out with similar results in **d**, **e**, **g**. Source data are provided as a Source Data file.

To investigate whether FATS directly regulates macrophage polarization, we analyzed mRNA and protein expression in primary mouse macrophages. First, RNA-sequencing analysis showed that the expression of genes associated with antigen presentation, T cell activation, and innate immunity was elevated in *Fats*^−/−^ BMDMs, while the expression of genes associated with immune suppression and chemoattraction was decreased in vitro under basal (in the presence of M-CSF) conditions (Fig. 4a), further confirming the significant changes in key M1/M2 markers on TAMs between WT and *Fats*^−/−^ mice-bearing melanoma tumors (Fig. 2d). Moreover, mRNA and protein levels were analyzed in vitro in primary mouse macrophages stimulated with lipopolysaccharide (LPS) or IL-4. The expression of proinflammatory cytokines that are associated with M1 macrophages was upregulated in *Fats*^−/−^ BMDMs. In contrast, the expression of genes associated with M2 macrophages (*Arg1* and *Ym1*) was inhibited (Fig. 4b, c and Supplementary Fig. 3a–c). Similar results were observed in *Fats*^−/−^ peritoneal macrophages (Supplementary Fig. 3d). In addition, the expression of CD80/86 and MHCI/II on BMDMs from WT and *Fats*^−/−^ mice was examined under different polarization conditions (Fig. 4d, e). B16 cell culture supernatant was used to culture BMDMs for 24 and 48 h (Fig. 4d). *Fats*^−/−^ BMDMs showed a higher expression of CD80/86 and MHCI/II than WT BMDMs after 24 h. Notably, MHCII and CD86 expression was significantly decreased in WT BMDMs after 48 h, while there was no obvious change in *Fats*^−/−^ BMDMs, suggesting that FATS deficiency increases M1 polarization under tumor conditions. Consistent with these results, under TAM, M2, and M1 polarization conditions (Fig. 4e), *Fats*^−/−^ BMDMs still had a higher expression of MHCII and CD86 than WT BMDMs, indicating the effect of FATS deficiency on skewing BMDMs toward M1 polarization. In addition, FATS deficiency enhanced the expression of MHCII on peritoneal macrophages (Supplementary Fig. 3e). Furthermore, silencing *FATS* in CD14^+^ monocyte-derived human macrophages increased the expression of M1-type proteins (HLA-DR and CD86) (Fig. 4g) and decreased that of M2-type genes (*MRC1, TGFB, ARG1, and IL10*) (Fig. 4h). The knockdown efficiency of *FATS* small interfering RNA (siRNA) was confirmed by Quantitative Real-time PCR, as shown in Fig. 4f. Collectively, these results confirm that FATS deficiency promotes macrophage polarization toward the M1-like phenotype.

### FATS deficiency sustains NF-κB activity

**Fig. 5:**
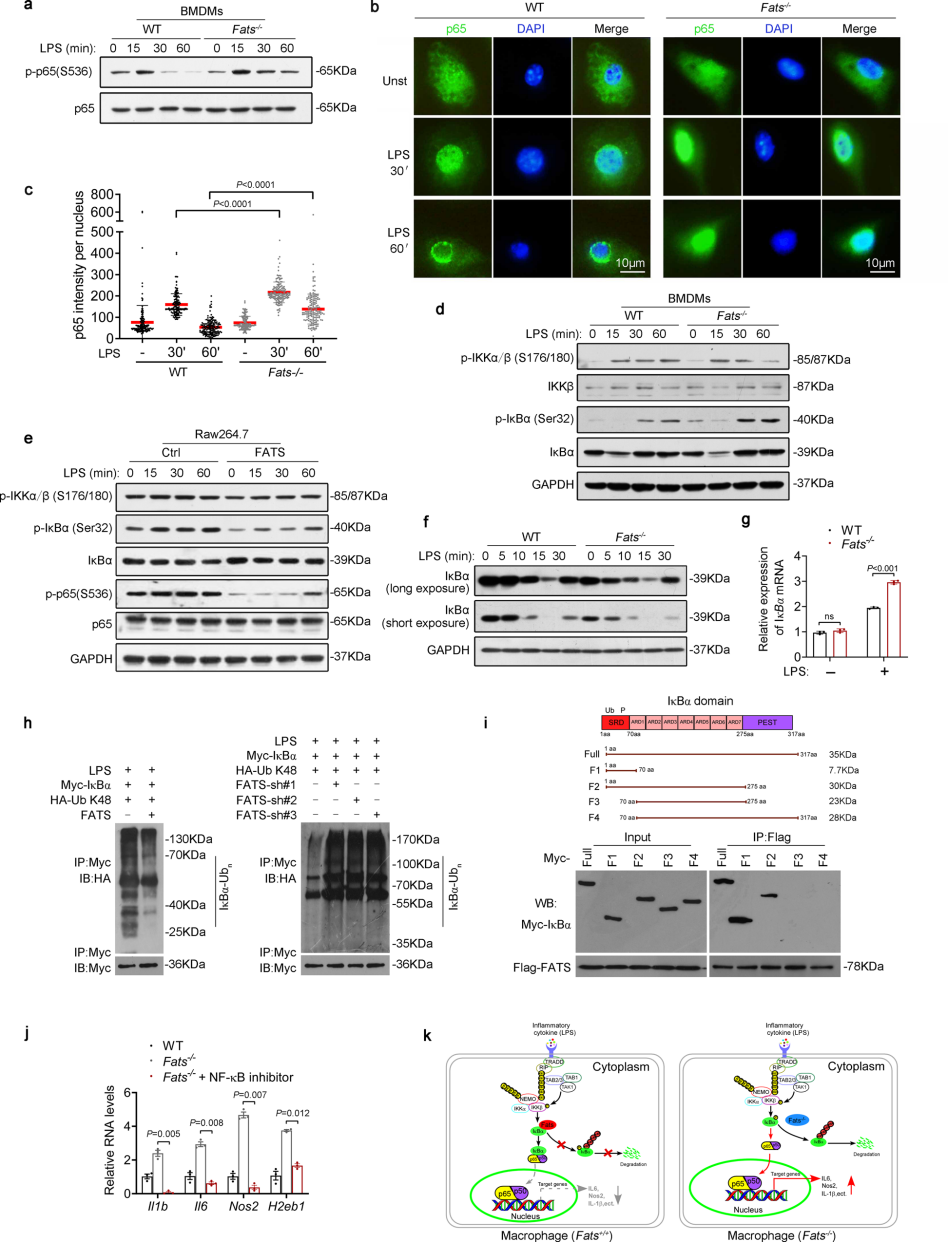
FATS deficiency sustains NF-κB activity. **a** Immunoblotting of p-p65/p65 in LPS-stimulated WT and *Fats*^−/−^ macrophages. **b** Nuclear translocation of p65 was assessed by immunofluorescence staining in WT and *Fats*^−/−^ macrophages scale bar = 10 μm. **c** Quantitative analysis of the fluorescence intensity of p65 in the nucleus of WT and *Fats*^−/−^ BMDMs. Data are presented as mean ± s.d. (*n* ≥ 150 cells for each group). **d** Immunoblotting of p-IKKα/β/IKKβ, p-IκBα/IκBα, and GAPDH in WT and *Fats*^−/−^ macrophages. **e** Immunoblotting of p-IKKα/β, p-IκBα/IκBα, p-p65/p65, and GAPDH in RAW264.7 mouse macrophages transduced with a lentiviral vector encoding mouse FATS or with empty vector (Ctrl). **f** Immunoblotting of endogenous IκBα in WT and *Fats*^−/−^ macrophages stimulated with LPS at different time points. **g** Relative mRNA expression of IκBα in WT and *Fats*^−/−^ macrophages stimulated with or without LPS. Data are presented as mean ± s.e.m. (*n* = 3 biologically independent samples). **h** Immunoblotting of K48-linked polyubiquitinated IκBα in the indicated cells. **i** Schematic illustration of wild-type and truncated IκBα (upper). Co-IP assays were performed using IκBα truncations, and their FATS binding activity was summarized. **j** mRNA expression levels in BAY11-7082-treated macrophages. Data are presented as mean ± s.e.m. (*n* = 3 biologically independent samples). **k** Schematic diagram showing that loss of FATS enhances the transcriptional activity of NF-κB via the promotion of K48-linked polyubiquitination of IκBα in macrophages. *P* values are calculated by two-tailed Student’s *t*-test in **c, g, j**. ns not significant. Two independent experiments were carried out with similar results in **a**, **d**–**f**, **h**, **i**. Source data are provided as a Source Data file.

LPS activates several signaling pathways, including the NF-κB, ERK, JNK, and p38/MAPK pathways as well as the signal transducer and activator of transcription (STAT) pathway in macrophages^27,28^. To investigate the mechanism of macrophage polarization under FATS-deficient conditions, intracellular TLR4 signaling was detected using western blot analysis. LPS induced p38/MAPK, ERK, JNK, NF-κB, and STAT activation by phosphorylation in both WT and *Fats*^−/−^ macrophages (Fig. 5a and Supplementary Fig. 4a, b). However, p-p65 activity was significantly enhanced in *Fats*^*−*^^/−^ macrophages compared with WT macrophages after LPS stimulation (Fig. 5a), whereas *Fats*^−^^/−^ macrophages displayed levels of p-p38, p-ERK, p-JNK, p-STAT1, and p-STAT3 similar to those in WT macrophages after LPS stimulation (Supplementary Fig. 4a, b). Similarly, increased p-p65 activity was also observed in *Fats*^*−/−*^ peritoneal macrophages compared with WT peritoneal macrophages (Supplementary Fig. 4c). Phosphorylation of the p65 subunit of NF-κB helps stabilize NF-κB in the nucleus for gene transcription^29^. Indeed, immunofluorescence staining showed that FATS deletion hampered the translocation of p65 from the nucleus to the cytoplasm in macrophages after LPS stimulation (Fig. 5b, c). We next examined the effect of FATS deletion on the stability and phosphorylation of proteins that regulate NF-κB activity, including IκBα and inhibitory kappa B kinase β (IKKβ). IKKβ promotes the degradation of IκBα, leading to the release of NF-κB from the inhibitory IκBα–NF-κB complex^30^. In LPS-stimulated *Fats*^*−/−*^ macrophages, FATS deletion increased the phosphorylation of IκBα, whereas there was no significant difference in the phosphorylation of IKKβ (Fig. 5d). To further verify the effect of FATS deletion on the NF-κB signaling pathway, the phosphorylation state of p65, IκBα, and IKKβ was detected in RAW264.7 cells transduced with a lentiviral expression construct encoding mouse FATS. We observed that phosphorylation of IκBα and p65 but not IKKβ was inhibited in FATS-expressing macrophages during the LPS response compared with that in control cells (Fig. 5e). These results indicate that FATS is an inhibitor of the TLR4–NF-κB activation pathway, and this inhibition may be associated with the regulation of IκBα phosphorylation.

To further explore the mechanism by which FATS regulates the NF-κB signaling pathway, degradation of endogenous IκBα was assessed in *Fats*^*−*^^/−^ and WT macrophages at different time points after LPS treatment. We observed that degradation of IκBα was enhanced in LPS-stimulated *Fats*^−/−^ macrophages compared with WT macrophages (Fig. 5f). In addition, the mRNA levels of IκBα were examined in *Fats*^*−*^^/−^ and WT macrophages under basal conditions or after stimulation with LPS. FATS deletion did not alter the IκBα mRNA level under basal conditions. Moreover, the mRNA level of IκBα was increased in *Fats*^−/−^ macrophages after LPS stimulation for 30 min (Fig. 5g), further confirming our previous conclusion that FATS deletion enhances the NF-κB transcriptional activity that triggers IκBα gene transcription. We also observed that the protein level of IκBα was higher in RAW264.7 cells infected with a lentiviral expression construct encoding mouse FATS than in control cells (Supplementary Fig. 4d). These results indicated that FATS suppressed the NF-κB activation pathway by inhibiting the degradation of IκBα.

It has been reported that FATS catalyzes the polyubiquitination of p53 mainly as K63-linked nonlinear polyubiquitination^31^. Herein, we detected the effect of FATS on K63-linked polyubiquitination of IκBα; however, we did not observe any obvious differences in K63-linked polyubiquitination of IκBα between *Fats*^−/−^ cells and control cells (data not shown), indicating that FATS dysregulation has no impact on K63-linked polyubiquitination of IκBα. Interestingly, we found that loss of FATS increased but overexpression of FATS significantly decreased the level of K48-linked polyubiquitination of IκBα (Fig. 5h). Furthermore, the co-immunoprecipitation (co-IP) assay showed that FATS interacts with the N-terminal signal response domain (SRD) of IκBα (Fig. 5i). The SRD can receive the phosphorylation signal and subsequent K48-linked ubiquitination signal, further mediating the degradation of IκBα by targeting the protein to the proteasome^32^. In addition, treatment with BAY11-7082, a specific NF-κB inhibitor, suppressed the inflammatory phenotype observed in *Fats*^*−*^^/−^ macrophages (Fig. 5j). Therefore, our data support the idea that FATS represses the K48-linked ubiquitination of IκBα by inhibiting the phosphorylation of IκBα by upstream kinases, subsequently promoting cytoplasmic accumulation of IκBα and repressing the transcriptional activity of NF-κB. Loss of FATS in macrophages enhanced the transcriptional activity of NF-κB via promotion of K48-linked polyubiquitination of IκBα and nuclear accumulation of NF-κB (Fig. 5k).

### *Fats*^*−*^^/−^ macrophages promote Th1 responses

**Fig. 6:**
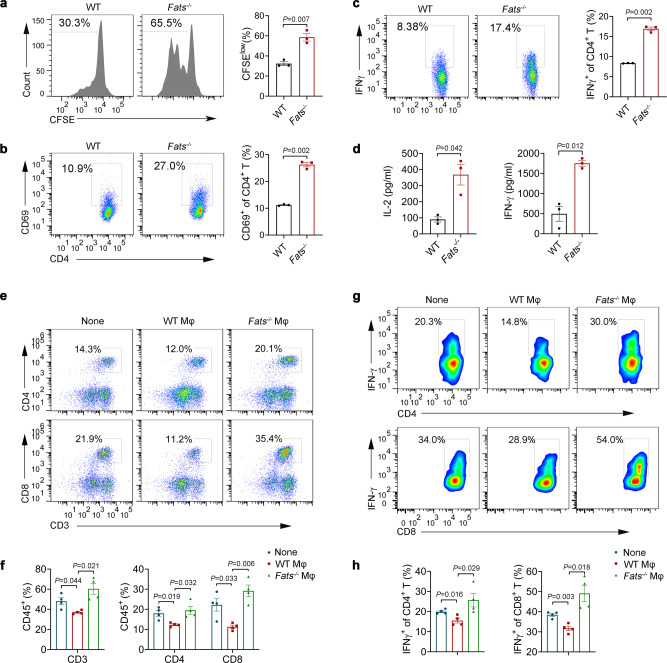
*Fats*^−/−^ macrophages promote Th1 responses. **a**–**d** BMDMs from WT and *Fats*^−/−^ mice were preincubated with soluble ovalbumin (OVA) overnight and cocultured with CFSE-labeled CD4^+^ OT-II T cells. **a** The proliferation of CD4^+^ OT-II T cells was analyzed using flow cytometry after coculture for 72 h. Flow cytometric analysis and quantification of CD69 (**b**) and IFN-γ (**c**) expression in CD4^+^ OT-II T cells after coculture for 72 h. **d** Supernatants were collected from BMDMs cocultured with OT-II CD4^+^ T cells after 3 days, and the IL-2 and IFN-γ concentrations were analyzed using ELISA kits (*n* = 3 biologically independent samples). **e**–**h** BMDMs from WT and *Fats*^−/−^ mice were transferred into recipient WT mice prechallenged with B16 cells. On day 20, tumors were isolated, and flow cytometry was used to determine the frequencies of tumor-infiltrating CD3^+^ T, CD4^+^ T, and CD8^+^ T cells among CD45^+^ cells (**e**, **f**) as well as to assess IFN-γ production in tumor-infiltrating CD4^+^ T and CD8^+^ T cells in B16 tumors (**g**, **h**) from recipient WT mice treated without (None) or with WT BMDMs (WT Mφ) or *Fats*^*−/−*^ BMDMs (*Fats*^*−/−*^ Mφ) (*n* = 4 mice per group). Data are presented as mean ± s.e.m. in **a**–**d**, **f**, **h**. *P* values are calculated by two-tailed unpaired Student’s *t*-test in **a**–**d**, **f**, **h**. Source data are provided as a Source Data file.

One of the hallmarks of M1 macrophage polarization is the acquisition of antigen-presenting features, which leads to efficient Th1 responses^33,34^. To investigate whether FATS deficiency promotes T cell proliferation and activation via macrophages, WT and *Fats*^−/−^ BMDMs were incubated with soluble ovalbumin (OVA) overnight and used to activate CD4^+^ OT-II and CD8^+^ OT- I T cells in vitro. We assessed T cell proliferation 3 days after coculture with WT or *Fats*^*−/−*^ BMDMs and analyzed the activation of specific T cell subsets by flow cytometry. WT BMDMs only weakly induced the proliferation and activation of antigen-restricted CD4^+^ T cells. Conversely, *Fats*^*−/−*^ BMDMs significantly induced CD4^+^ T cell proliferation (CFSE), CD4^+^ T cell activation (CD69), and the Th1 response (IFN-γ), as indicated by the increases in the population of IFN-γ-producing CD4^+^ cells (Fig. 6a–c) and protein expression levels of IL-2 and IFN-γ (Fig. 6d). However, FATS deficiency did not influence CD8^+^ T cell proliferation, as evidenced by the similar CFSE levels in OT-I cells after coculture with LPS-stimulated or LPS-unstimulated *Fats*^*−/−*^ or WT BMDMs (Supplementary Fig. 5a). These results were consistent with our previous finding that FATS blockade in macrophages substantially increased the expression of MHCII but not MHCI under M1 polarization conditions (Fig. 4e and Supplementary Fig. 5b). To further verify the effect of *Fats*^*−/−*^ macrophages on the adaptive immune system in vivo, WT and *Fats*^*−/−*^ BMDMs were transferred into recipient WT mice prechallenged with B16 cells. The tumor-specific T cell response within the tumors was analyzed by flow cytometry on day 20. The numbers of both CD4^+^ and CD8^+^ T cells were significantly increased (Fig. 6e, f), and IFN-γ expression in both CD4^+^ T and CD8^+^ T cells was substantially increased in the tumors of mice transferred with *Fats*^*−/−*^ BMDMs but not in those transferred with WT BMDMs (Fig. 6g, h). Together, these results indicated that FATS-deficient macrophages directly promoted CD4^+^ T cell proliferation and Th1 responses in vivo and in vitro but may indirectly promote CD8^+^ T cell responses in vivo.

Numerous studies have shown that Th1 cells can generate, augment, and maintain tumor-specific CD8^+^ CTLs responses^35^. For example, IL-2 secreted by Th1 cells acts as a growth factor for CTLs^36^ and functions to recruit CTLs and retain them at the tumor site. IFN-γ production by Th1 cells results in upregulation of MHC molecules on tumor cells, leading to enhanced recognition by T cells (CTLs and Th cells). Therefore, we speculated that the activation of CD8^+^ T cells in mice adoptively transferred with *Fats*^−/−^ BMDMs was potentially caused by macrophage-mediated activation of Th1 cells. Collectively, these data further reveal that FATS deficiency stimulates macrophage polarization toward the antitumor phenotype (M1), which has an enhanced capacity to activate Th1 responses.

### FATS deficiency promotes antitumor T cell activity within the TME

**Fig. 7:**
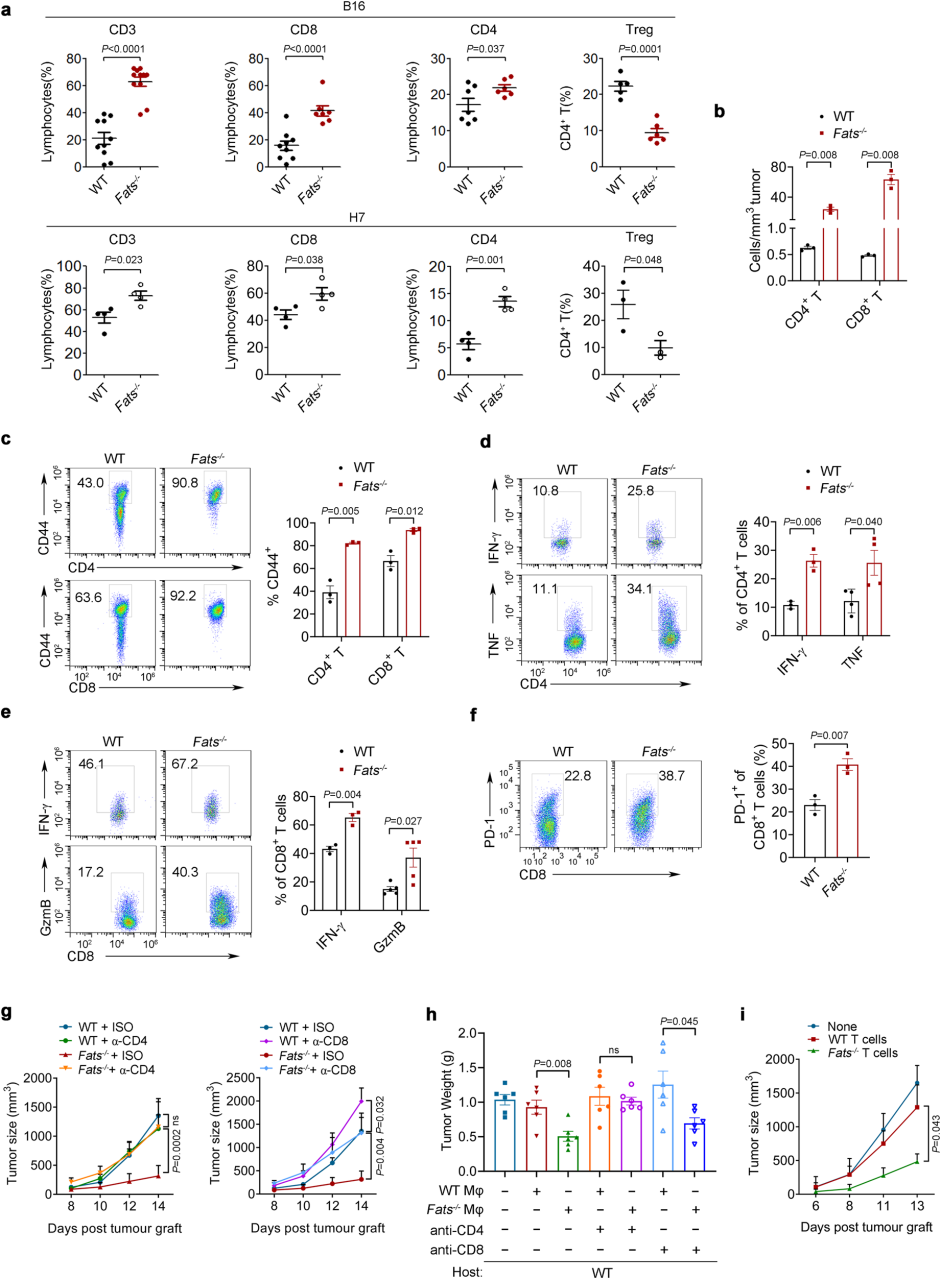
FATS deficiency promotes antitumor T cell activity within the TME. **a** Phenotypic analysis of tumor-infiltrating T cells after subcutaneous injection of B16 and H7 cells. Total CD3^+^, CD8^+^, CD4^+^, and regulatory T cell (Treg: CD4^+^CD25^+^Foxp3^+^) populations among CD45^+^ cells in B16 tumors (WT *n* = 10 mice, *Fats*^*−/−*^ *n* = 11 mice for CD3; WT *n* = 9 mice, *Fats*^*−/−*^ *n* = 7 mice for CD8; WT *n* = 7 mice, *Fats*^*−/−*^ *n* = 6 mice for CD4; WT *n* = 5 mice, *Fats*^*−/−*^ *n* = 6 mice for Treg) and H7 tumors (*n* = 4 mice per group for CD3, CD8, and CD4; *n* = 3 mice per group for Treg). **b** The densities of tumor-infiltrating CD4^+^ T and CD8^+^ T cell populations in B16 tumors (*n* = 3 mice per group). **c** Flow cytometric analysis and quantification of surface CD44 expression on tumor-infiltrating CD4^+^ T and CD8^+^ T cells in B16 tumors (*n* = 3 mice per group). **d** Tumor-infiltrating CD4^+^ T cells were gated and assessed for the expression of IFN-γ (*n* = 3 mice per group) and TNF (*n* = 4 mice per group) in B16 tumors. **e** Tumor-infiltrating CD8^+^ T cells were gated and assessed for expression of IFN-γ (*n* = 3 mice per group) and Granzyme B (*n* = 5 mice per group) in B16 tumors. **f** Flow cytometric analysis and quantification of PD-1 expression on tumor-infiltrating CD8^+^ T cells in B16 tumors (*n* = 3 mice per group). Data are presented as mean ± s.e.m. in **a**–**f**; *P* values are calculated by two-tailed Student’s *t*-test in **a–f**. **g** CD4^+^ T or CD8^+^ T cells were depleted as indicated. The mean tumor volumes in WT and *Fats*^−/−^ mice are shown. Data are presented as mean ± s.e.m. (*n* = 6 mice per group; two-way ANOVA). **h** CD8^+^ T or CD4^+^ T cells were depleted in host WT mice. B16 tumor burdens after treatment with WT or *Fats*^−/−^ macrophages are shown. Data are presented as mean ± s.e.m. (*n* = 6 mice per group; two-tailed Student’s *t*-test). **i** Mean B16 tumor volumes after adoptive transfer of tumor-infiltrating T cells from WT and *Fats*^−/−^ mice. Data are presented as mean ± s.e.m. (*n* = 6 mice per group; two-way ANOVA). ns not significant. All tumor samples were collected on day 20 (melanoma) or day 30 (pancreatic cancer) after tumor cell implantation. Source data are provided as a Source Data file.

To investigate whether FATS deficiency in situ leads to enhanced T cell-mediated antitumor activity within the TME, CD4^+^ and CD8^+^ T cell phenotypes were examined in tumor-bearing WT and *Fats*^−/−^ mice. Consistent with the results from our macrophage adoptive transfer experiment, FATS deficiency significantly increased the percentages and numbers of CD4^+^ T cells and CD8^+^ T cells while decreasing the number of regulatory T cells (Tregs) in both B16 and H7 tumors (Fig. 7a, b). The gating strategy for T cell subsets is shown in Supplementary Fig. 1b. FATS deficiency enhanced the expression of CD44, another commonly used marker for T cell activation, on CD4^+^ T and CD8^+^ T cells (Fig. 7c). Furthermore, FATS deficiency increased IFN-γ and TNF levels in B16 tumor-infiltrating CD4^+^ T cells (Fig. 7d), suggesting enhanced Th1 responses. Similarly, FATS deletion increased the expression of Granzyme B and IFN-γ in B16 tumor-infiltrating CD8^+^ T cells (Fig. 7e), which indicated the positive role of FATS deficiency in CTL activation^37^. PD-1 expression was also increased in CD8^+^ T cells (Fig. 7f). In addition, in the model of B16 melanoma lung metastasis, pLNs and mLNs were larger in *Fats*^−/−^ mice than in WT mice (Supplementary Fig. 6a). The percentages of both CD4^+^ T cells and CD8^+^ T cells were increased in LNs of *Fats*^−/−^ mice compared with WT mice (Supplementary Fig. 6b). Moreover, activation of CD4^+^ T cells and CD8^+^ T cells was significantly higher in the lungs of *Fats*^−/−^ mice than in those of WT mice (Supplementary Fig. 6c).

In addition, we tested the requirement for the adaptive immune system by implanting B16 tumor cells into WT and *Fats*^−/−^ mice subjected to antibody-mediated depletion of CD4^+^ or CD8^+^ T cells. Depletion of either CD4^+^ or CD8^+^ T cells partially abrogated the protective effects of FATS deficiency (Fig. 7g). The efficiency of CD4^+^ T cell depletion (Supplementary Fig. 7a) and CD8^+^ T cell depletion (Supplementary Fig. 7b) in spleen and tumor tissues was confirmed. However, FATS deficiency did not reduce the tumor burden in the context of CD4^+^ T cell depletion (Fig. 7g and Supplementary Fig. 7c). CD8^+^ T cell depletion reversed the inhibition of tumor growth on day 10 but not on day 16 or at later time points in *Fats*^−/−^ mice (Supplementary Fig. 7c), suggesting that *Fats*^*−/−*^ macrophages control tumor growth in a manner mainly dependent on CD4^+^ T cells. To eliminate the influence of FATS-deficient T cells, we further depleted CD4^+^ and CD8^+^ T cells only in WT mice and then transferred these mice with WT BMDMs or *Fats*^−/−^ BMDMs. As expected, only after CD4^+^ T cell depletion did the tumor weight in host WT mice show no significant change between mice transferred with WT BMDMs and those transferred with *Fats*^−/−^ BMDMs (Fig. 7h). Moreover, tumor-infiltrating T cells were isolated from B16 tumor-bearing WT or *Fats*^−/−^ mice, mixed with B16 tumor cells and adoptively transferred into new WT recipient mice. Compared with transfer of tumor-entrained WT T cells, adoptive transfer of T cells from B16 tumor-bearing *Fats*^−/−^ mice protected against B16 tumor growth (Fig. 7i). These findings confirm that strategies targeting FATS could enhance T cell-mediated antitumor activity.

### FATS is a potential target for tumor immunotherapy

**Fig. 8:**
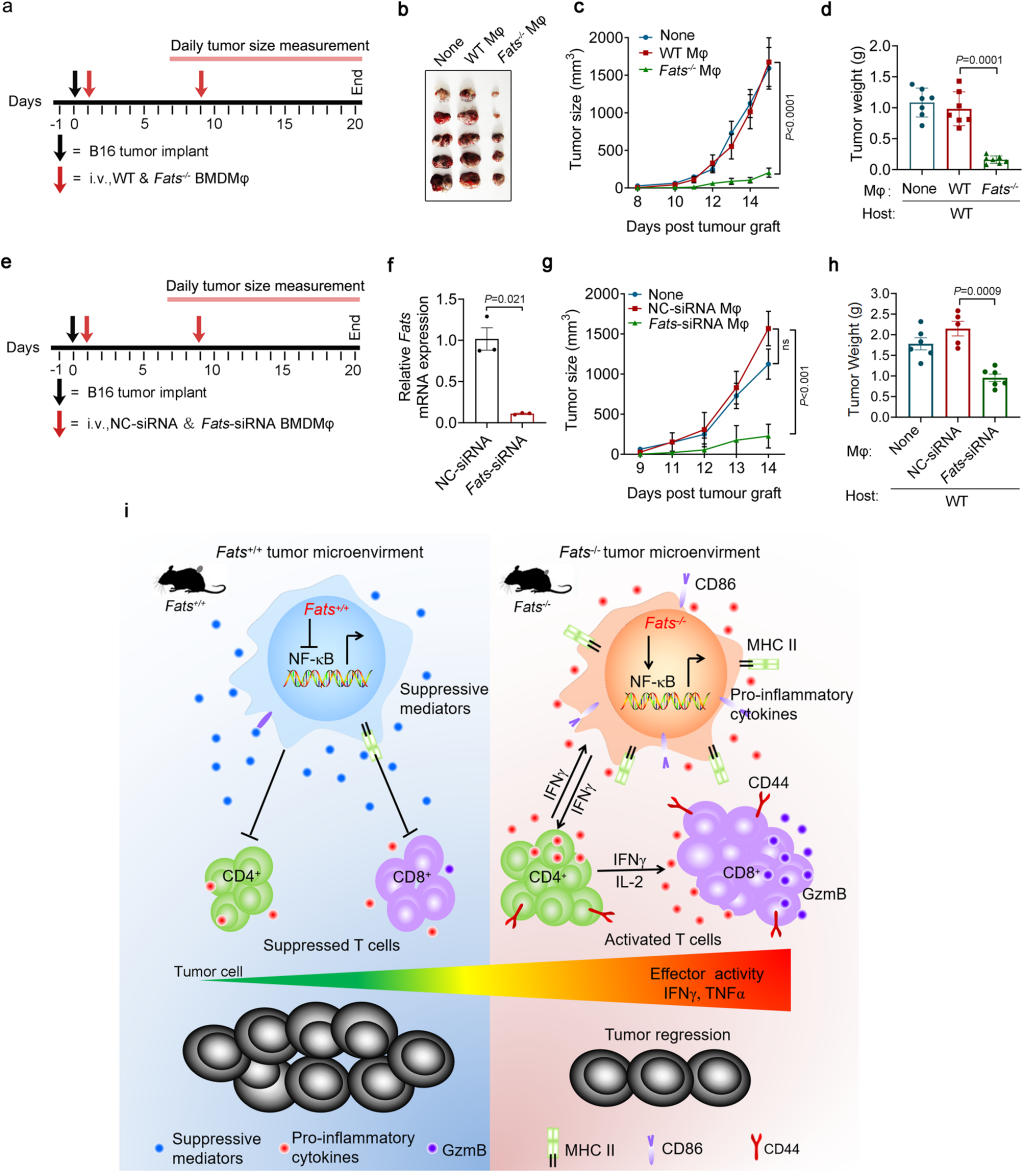
FATS is a potential target for tumor immunotherapy. **a** Schematic of the BMDMs adoptive transfer experiment. **b**–**d** Representative image (*n* = 5 mice per group), tumor volumes (*n* = 7 mice per group; two-way ANOVA) and quantitative analysis of the weights (*n* = 7 mice per group; two-tailed Student’s *t*-test) of B16 tumors in recipient WT mice treated without (None) or with WT BMDMs (WT Mφ) or *Fats*^*−/−*^ BMDMs (*Fats*^−/−^Mφ) from **a** on day 20. **e** Schematic of the siRNA-transfected BMDMs adoptive transfer experiment. BMDMs treated with nontargeting control (NC) or *Fats* siRNA were intravenously injected into recipient WT mice on day 1 and day 9 separately. **f** The relative mRNA level of *Fats* was measured by Quantitative Real-time PCR, and the efficiency of siRNA was confirmed (*n* = 3 biologically independent samples). **g, h** Tumor volumes (*n* = 6 mice per group; two-way ANOVA) and quantitative analysis of the tumor weights in recipient WT mice treated without (None) or with BMDMs transfected with NC siRNA (NC-siRNA Mφ) or *Fats* siRNA (*Fats*-siRNA Mφ) (*n* = 6 mice for none and *Fats*-siRNA Mφ group, *n* = 5 mice for NC-siRNA Mφ group; two-tailed Student’s *t*-test) of B16 tumors in recipient WT mice from **e** on day 20. The data are representative of two independent experiments with similar results. **i** Model depicting the effect of FATS deficiency on tumor immune stimulation. FATS signaling in macrophages inhibits the activation of NF-κB and induces an immunosuppressive M2-like state featuring abundant secretion of inhibitory cytokines and suppression of CD4^+^ and CD8^+^ T cell activation (left panel). By contrast, FATS deficiency promotes the NF-κB activation and shifts macrophage polarization toward an M1-like phenotype characterized by higher antigen presentation capability and secretion of numerous proinflammatory cytokines; this phenotype further promotes the proliferation of CD4^+^ T cells and induces Th1 responses, which in turn promote CD8^+^ T cell priming and thereby enhances antitumor immunity (right panel). Data are presented as mean ± s.e.m. in **c**, **d**, **f**–**h**. ns not significant. Two independent experiments were carried out with similar results in **a**–**d**. Source data are provided as a Source Data file.

Since FATS signaling blockade promotes M1 polarization, which can stimulate Th1 and CTL responses, we sought to investigate whether FATS blockade could be used as a potential tumor immunotherapy approach. To this end, WT and *Fats*^−/−^ BMDMs were adoptively transferred into recipient WT mice pre-challenged with B16 cells, as shown in Fig. 8a. B16 tumor growth was significantly inhibited in host mice adoptively transferred with *Fats*^−/−^ BMDMs but not in those adoptively transferred with WT BMDMs (Fig. 8b–d). Similar results were observed when host mice were adoptively transferred with nontargeting control (NC) siRNA- or *Fats* siRNA-transfected BMDMs (Fig. 8e–h). These results suggest that FATS functions as a molecular switch and could be a therapeutic target for enhancing antitumor immunity (Fig. 8i).

## Discussion

FATS is involved in DNA damage-induced tumorigenesis^21,24^. Its functional deficit leads to a high frequency of localized deletion events in the genome under replication stress, such as that imposed by IR^21^. Therefore, affected individuals are at increased risk of developing tumors caused by chromosomal aberrations. Previous studies demonstrated that FATS functions as a tumor suppressor, since *Fats* was susceptible to breakage in an IR-induced mouse tumor model and in other types of cancer cells^21,24^. In the current study, we found that the growth of transplanted tumors (generated from B16 melanoma cells) was significantly inhibited in *Fats*^−/−^ mice and that the survival of tumor-bearing *Fats*^−/−^ mice was prolonged. We tried to identify the potential mechanisms accounting for this discrepancy and found a significant change in macrophages between *Fats*^−/−^ mice and WT mice. In addition, we analyzed samples from patients with several types of cancer by using TCGA data (Supplementary Fig. 8), which showed that lower expression of FATS correlated with a higher overall survival rate. These findings encouraged us to further explore the mechanism of FATS in the immune response. Based on our results, we believe FATS may play a role similar to that of immune checkpoints. Specifically, FATS may act as a tumor promoter in the context of immune cell function during cancer development. Furthermore, given that CFSs are an inherent component of chromosomal structure in all individuals and are evolutionarily conserved, they may play a protective role against cancer or other diseases. CFSs preferentially form gaps or breaks on metaphase chromosomes upon replication stress, viral integration, and amplicon induction. Thus, we speculate that in somatic cells, deletion of a fragile site gene usually causes the cells to become cancerous, but at the same time, in immune cells, brittle fracture or lack of a gene locus may spontaneously induce a self-defense system to remove the mutant cells to protect the body; however, this mechanism is just speculatory, and subsequent elegantly designed experiments are needed to verify our hypothesis.

In the context of cancer, the host plays important roles in regulating the strength and timing of the anticancer response. The host immune system has the ability to identify and eliminate cancerous/transformed cells^1^. For instance, the risk of developing cancers is increased when the immune system is compromised^38,39^. Macrophages are essential components of the immune system and are necessary for a protective host defense. However, TAMs are very vulnerable to tumor conditions and adapt to become accomplices (M2 macrophages) of the tumor in vivo at both the primary site and metastatic sites. TAMs can produce growth factors in the TME and participate in intercellular interactions^8,25,40–42^. Based on these findings, we examined the immune profile (particularly focusing on TAMs) of the TME in both WT and *Fats*^*−/−*^ mice bearing melanoma tumors. A significant difference was found between WT and *Fats*^*−/−*^ mice, as after FATS deficiency, M2-like TAM infiltration into tumors was reduced, while M1-like TAM infiltration was increased. Because M1-like macrophages have good antitumor properties, while M2-like macrophages have the opposite properties^15^, these results further support our hypothesis that FATS-deficient macrophages show antitumor activity. Consistent with these results, the in vitro study indicated that *Fats*^*−/−*^ macrophages exhibited an intrinsic bias toward M1 polarization, as evidenced by the high expression of proinflammatory cytokines. In addition, M2 polarization of both BMDMs and peritoneal macrophages from *Fats*^*−/−*^ mice was suppressed. The results of transduction of *FATS* siRNA in human macrophages also confirmed the function of FATS in regulating macrophage polarization. Using the in vivo macrophage adoptive transfer approach, we further proved that *Fats*^*−/−*^ macrophages significantly inhibited B16 tumor growth, suggesting that strategies that manipulate macrophage polarization by targeting FATS may be attractive immunotherapies for cancer.

Macrophage polarization is a complex process. It involves the activation of many intracellular signaling pathways. Some studies have pointed out that JNK signaling is required for the polarization of adipose tissue-related macrophages toward an M1-like phenotype^43^. Gene ablation experiments showed that Akt1 and 2 interact to regulate M1 and M2 macrophage polarization^44^. JAK/STAT pathways are also involved in macrophage polarization^45^. It has also been found that metformin can promote M1 macrophages and inhibit M2 macrophages through the Notch signaling pathway, finally inhibiting the growth of liver cancer cells^46^. However, complete polarization of macrophages to the M1 type requires activation of the TLR-4 and IFN-γ signaling pathways^47,48^. NF-κB is claimed to be the master regulator of inflammation and immunity^30^. Recent studies have indicated that the NF-κB signaling pathway plays an important role in M1 polarization of macrophages^47,49^. Duygu Sag et al.^50^ demonstrated that *Abcg1* gene defects can activate the NF-κB signaling pathway and promote the polarization of M1 macrophages, which ultimately leads to significant inhibition of melanoma growth. Consistent with these results, we observed that the NF-κB signaling pathway was significantly activated in *Fats*^*−/−*^ macrophages. Blocking NF-κB activity significantly reversed the increases in MHCII and proinflammatory cytokine expression in LPS-stimulated *Fats*^*−/−*^ macrophages, indicating that NF-κB activity is involved in FATS-mediated regulation of M1 polarization. NF-κB is reported to be constitutively activated in transformed cells in many kinds of tumors and contribute to cell survival^51^. Treatment with an NF-κB agonist can promote the polarization of macrophages toward the M1 type in tumors and exert antitumor effects, but it can also promote the proliferation and invasion of tumor cells^52^. Therefore, it is important to delete or downregulate FATS expression only in immune cells, a strategy that could effectively avoid this limitation; in other words, NF-κB signaling could be activated in macrophages with no or low FATS expression without promoting tumor cell activation. Furthermore, we found that FATS deficiency sustains NF-κB activity by disrupting NF-κB/IκBα-negative feedback loops and further promotes the polarization of macrophages toward the M1 phenotype.

Moreover, *Fats*^*−/−*^ macrophages expressed high levels of MHCII molecules and the costimulatory molecule CD86, thereby exhibiting a more potent antigen presentation function. In addition, *Fats*^*−/−*^ macrophages exhibited increased production of IL-12, which promotes the secretion of IFN-γ by T, NK, and NKT cells and induces Th1 responses^53^. An increasing number of studies have reported the crucial role of activated Th1 cells in effective antitumor immunity^35,54,55^, and infiltration of these cells into tumor tissue is an indicator of more favorable prognosis in pancreatic cancer^56^. Consistent with published results, *Fats*^*−/−*^ macrophages promoted the proliferation and activation of CD4^+^ T lymphocytes and steered them toward the Th1 phenotype in vitro. Accordingly, transfer of *Fats*^*−/−*^ BMDMs increased the number of CD4^+^ T cells and promoted Th1 responses. CD4^+^ Th-derived cytokines are required for full activation of CD8^+^ T cells, which can give rise to tumor-specific CTLs^35,57^. This finding may explain why in the in vitro experiment, *Fats*^*−/−*^ macrophages did not increase the activation of CD8^+^ T cells, whereas transfer of *Fats*^*−/−*^ BMDMs substantially increased the activation of cytotoxic CD8^+^ T cells within the tumor. In addition, IFN-γ production by CD4^+^ Th1 cells further promoted macrophage polarization toward the M1 phenotype, indicating a positive feedback loop in M1 macrophage polarization.

Together, these results indicate that FATS deficiency significantly increases the activation of both macrophage-mediated innate and T cell-mediated adaptive antitumor immune responses while inhibiting immunosuppressive responses, suggesting that FATS may play a role similar to that of immune checkpoints. In this study, we provide evidence for the potential applications of FATS in cancer immunotherapy.

## Methods

### Cell lines and plasmids

The mouse melanoma cells (B16), pancreatic cancer cells (H7), RAW264.7 cells, and human HEK293T cells were obtained from American Type Culture Collection, and authenticated using short tandem repeat profiling. All cells were cultured in DMEM (Gibco) supplemented with 10% FBS (HyClone) and 1% penicillin–streptomycin (Gibco). All cells were cultured at 37 °C in an atmosphere of 5% CO_2_ in air. Cells were injected into mice after reaching 60–80% confluence. Myc-IkBa and HA-UB K48 expression constructs were purchased from Vigene Biosciences. The FATS overexpression plasmid was purchased from GeneCopoeia.

### Animal care

Female C57BL/6 mice (6–8 weeks old), FATS knockout C57BL/6 mice (*Fats*^*−/−*^), OT-I and OT-II TCR transgenic C57BL/6 mice were maintained under SPF conditions in a controlled environment of 20–22 °C, with a 12/12 h light/dark cycle, 50–70% humidity, and food and water provided ad libitum. C57BL/6 female mice were purchased from the Academy of Military Medical Science (Beijing, China) and acclimated for 7 days in the laboratory before experimentation. *Fats*^*−/−*^ mice, constructed using the TALEN-mediated double-hit genome modification technique on a C57BL/6 background, were provided by Professor Zheng Li of Tianjin Medical University Cancer Hospital. OT-I and OT-II TCR transgenic mice were originally obtained from The Jackson Laboratory. All animal experiments were performed in accordance with the guidelines for animal care and approved by the Animal Ethics Committee of Tianjin Medical University (Permit Number: SYXK 2009-0001).

### Tumor studies

WT and *Fats*^*−/−*^ mice were challenged with B16 (2 × 10^5^ cells per mouse) or H7 cells (1 × 10^6^ cells per mouse) by subcutaneous injection. All tumor cell lines formed solid tumors in mice. Tumors were measured every 2 days with a caliper, and the volume (length × (width)^2^/2) was calculated. Mice that had no visible or palpable tumors that could be measured on consecutive measurement days were considered to exhibit complete regression. Animals were euthanized if they exhibited signs of distress or when the total tumor volume reached 2500 mm^3^. The tumors, spleens, and draining lymph nodes were collected and subjected to further analysis. In the model of lung-metastatic melanoma, B16 cells (5 × 10^5^ cells per mouse) were intravenously injected into WT and *Fats*^*−/−*^ mice (aged 8–10 weeks). To evaluate tumor metastasis, mice were euthanized on day 20, and tumors in the lungs were counted. Spleens, lymph nodes, and lung tissues were collected and subjected to further analysis.

### Isolation of single cells from mouse immune organs and tumors

Single-cell suspensions derived from spleens and draining lymph nodes were prepared by mechanical disruption and filtering through a 70-μm cell strainer (BD Biosciences). Red blood cells were solubilized with red cell lysis buffer (Solarbio). For preparation of cells from tumor tissues, tumors were isolated, minced into small pieces, and digested with type-IV collagenase, hyaluronidase, and DNase I (0.05 mg/ml each; Sigma, USA) for 30 min at 37 °C. Single-cell suspensions were obtained by grinding the digested tissues and filtering them through a 70-μm cell strainer. Red blood cells were solubilized with red cell lysis buffer (Solarbio). Tumor-infiltrating mononuclear cells were isolated using Ficoll density gradient centrifugation and analyzed by flow cytometry. Cells were washed once with PBS prior to use for flow cytometric analysis or magnetic bead purification.

### Flow cytometric analysis

Single-cell suspensions from the indicated tissues were prepared as mentioned above. Cells were then washed once, blocked using anti-CD32/CD16 (BioLegend) to avoid non-specific binding, and stained with antibodies to cell surface markers (Supplementary Table 2). For intracellular transcription factor staining, cells were fixed and permeabilized with a FoxP3 Staining Buffer Set (eBioscience) and were then stained with FoxP3 antibody. For intracellular cytokine staining, cells were restimulated with Cell Stimulation Cocktail (eBioscience) for 4 h at 37 °C. Intracellular staining was performed using a Fixation & Permeabilization Buffer Set (eBioscience) by incubation for 30 min at 4 °C and stained with antibodies to cytokines (IFN-γ, TNF and GranzymeB) according to the manufacturer’s instructions. Stained cells were acquired by flow cytometry using a BD FACSCanto II, and the data were processed using FlowJo software (version 7.6.1). Dead cells were excluded on the basis of forward and side scatter. Antibody information is listed in Supplementary Table 2.

### Purification of myeloid cells and TAMs from tumors

Single-cell suspensions from mouse tumors were prepared as described in the previous section. Tumor cells were subsequently separated from debris over a Ficoll gradient (Sigma-Aldrich). Myeloid cells were stained with anti-mouse CD11b antibody for flow sorting in a FACSAria II cell sorter in the flow cytometry core facility at the basic medical research center. In another experiment, TAMs were stained with anti-mouse CD11b and anti-mouse F4/80 antibodies for flow sorting in a FACSAria II cell sorter (BD Biosciences). Isotype control antibodies were used as negative controls. The purity of the flow-sorted populations was above 90%. The cells were then harvested for subsequent T cell suppression tests and characterized by Quantitative Real-time PCR. Antibody information is listed in Supplementary Table 2.

### T cell suppression assay

Spleens from WT mice were isolated and passed through 40 μm filters to generate a single-cell suspension. After red blood cells lysis, CD3^+^ T cells were purified using CD3 microbeads (Miltenyi Biotech) according to the manufacturer’s protocol and labeled with 2.5 μM CFSE (Invitrogen) for 10 min at 37 °C. The labeled CD3^+^ T cells were intensively washed with PBS and were then plated in complete RPMI medium supplemented with 0.05 M β-mercaptoethanol in round-bottom 96-well plates (1 × 10^5^ cells per well) coated with 5 μg/ml anti-CD3 (Invitrogen, 16-0031-86) and 2 μg/ml anti-CD28 (Invitrogen, 16-0281-85) antibodies. Purified TAMs (CD11b^+^F4/80^+^) prepared as described above were added at the indicated ratios, and the plates were incubated at 37 °C. After 72 h, the cells were harvested, and the CFSE signal in the gated CD3^+^ T cell population was measured by flow cytometry.

### Cell depletion experiments

WT and *Fats*^*−/−*^ female mice treated with or without i.v. injection of 1 mg/mouse clodronate liposomes or PBS liposomes (Clodronate Liposomes.com, Amsterdam, The Netherlands) were used for in vivo macrophage depletion experiments. Treatment started one day before challenge with B16 tumor cells and continued every 4 days (Q4D) during the study. Tumor measurements were taken every 2 days. When the average volume of the tumors was 2500 mm^3^, mice were euthanized, tumor tissues were collected, and tumor-infiltrating mononuclear cells were isolated and analyzed as mentioned above. For in vivo depletion of CD4^+^ T or CD8^+^ T cells, 200 μg anti-CD4 (Sungene, clone: GK1.5) or anti-CD8a (Sungene, clone: 53-6.7) antibodies was injected intraperitoneally beginning 1 day before tumor implantation and continuing every 4 days (Q4D) for the duration of the study. Control animals were injected intraperitoneally with isotype control antibody (Sungene, clone: MPC-11) on the same dosing schedule. CD4^+^ or CD8^+^ T cell depletion was verified by flow cytometric analysis of spleens and tumor tissues.

### Peritoneal macrophage isolation

Peritoneal macrophages were harvested in 5 ml of PBS from the mouse peritoneal cavity 72 h after intraperitoneal injection of 3% thioglycollate solution. After washing with PBS, cells were cultured in RPMI medium supplemented with 10% FBS and 1% pen/strep for 2 h at 37 °C in 5% CO_2_. After 2 h, nonadherent cells were removed by washing three times with PBS, and cells were analyzed via flow cytometry or Quantitative Real-time PCR or were stimulated with LPS and analyzed by immunoblotting. The purity of CD11b^+^F4/80^+^ cells was verified to be 98% by flow cytometry. In some studies, peritoneal macrophages were preincubated with an inhibitor of NF-κB (BAY11-7082, 5 μM, MCE) for 2 h prior to the addition of polarizing stimuli.

### Mouse macrophage differentiation

Bone marrow-derived cells were aseptically harvested from 6–8-week-old female mice by flushing the femurs and tibias of euthanized mice. Red blood cells were solubilized with red cell lysis buffer (Solarbio), and the remaining cells were cultured in RPMI medium supplemented with 10% serum and 10 ng/ml recombinant murine M-CSF (PeproTech, 315–02) for 6 days; fresh medium was added on day 3. The purity of CD11b^+^F4/80^+^ macrophages was verified to be 95% by flow cytometry.

### Human macrophage differentiation

Fresh human buffy coat blood was obtained from Tianjin Blood Center (Tianjin, China). Ethical consent was obtained by the Tianjin blood center and samples were anonymised before use. Ficoll-Paque PLUS (GE Healthcare Biosciences AB, 17-0891-09) was used to isolate peripheral blood mononuclear cells (PBMCs). Cells were centrifuged at 400 × *g* for 30 min and washed with PBS three times to obtain PBMCs. CD14^+^ monocytes were isolated from PBMCs using CD14 MicroBeads (Miltenyi Biotec, 130-050-201) according to the manufacturer’s instructions. Purified CD14^+^ monocytes were analyzed using flow cytometry to ensure their purity (>95%). Primary CD14^+^ cells were incubated with 50 ng/ml recombinant human M-CSF (PeproTech, 300-25) for 6 days, and fresh medium was added on day 3.

### Macrophage polarization

To induce M1-like polarization, bone marrow-derived cells (BMDCs) were stimulated with recombinant murine GM-CSF (PeproTech, 10 ng/ml) for 6 days followed by IFN-γ (Sigma, 20 ng/ml) and LPS (Sigma, 100 ng/ml) for 24 h. To induce M2-like polarization, BMDCs were stimulated with M-CSF (PeproTech, 10 ng/ml) for 6 days followed by M-CSF (PeproTech, 10 ng/ml) and IL-4 (PeproTech, 20 ng/ml) for 24 h. To induce TAM polarization, BMDCs were stimulated with M-CSF for 6 days followed by IL-4 (PeproTech, 20 ng/ml) and IL-10 (PeproTech, 20 ng/ml) for 24 h. For inhibitor studies, macrophages were incubated with BAY11-7082 (MCE, 5 μM) for 2 h prior to the addition of polarizing stimuli. Cells were harvested and analyzed by Quantitative Real-time PCR or flow cytometry.

### Gene silencing and transfection

Macrophages prepared as described above were transfected with 20 nM targeting siRNA or nontargeting control siRNA (NC-siRNA) using Lipofectamine RNAiMAX Reagent according to the manufacturer’s instructions (Invitrogen). siRNAs targeting mouse *Fats* (5′-GCAACATGTACCAGTAGCA) and human *FATS* (5′-GAGATCAAATTGCCCTTAA) and NC-siRNA were purchased from RiboBio. After transfection, cells were cultured in RPMI medium containing 10% serum and recombinant murine or human M-CSF (PeproTech) for 48 h or were polarized as described above. For gene transfection, HEK293T cells were allowed to grow to 60–70% confluence and were then transfected with 2 µg of the indicated vectors using PEI. *FATS*-sh#1 (5′-GCTCAACCTCAATTGTAGTTC), *FATS*-sh#2 (5′-CCTCAGTTCATTTCTCGCTCA), and *FATS*-sh#3 (5′-CCTCTGAGTGATAACTTGTTC) were purchased from GeneCopoeia. After 72 h, cell lysates were collected and analyzed by immunoblotting and immunoprecipitation.

### In vivo macrophage adoptive transfer experiment

A total of 2 × 10^5^ B16 tumor cells were injected subcutaneously into WT mice on day 0 of the experiment. Primary BMDMs were harvested from donor WT and *Fats*^*−/−*^ mice and prepared into a single-cell suspension. On day 1 of the experiment, purified BMDMs (5 × 10^5^ cells per mouse) were injected intravenously into B16 cell-challenged host WT mice. On day 9 after tumor cell implantation, B16 cell-challenged host WT mice received the second macrophage adoptive transfer treatment administered via the same protocol as the first treatment. Tumor dimensions were measured three times per week beginning on day 7.

### T cell adoptive transfer

WT and *Fats*^*−/−*^ mice were implanted with 2 × 10^5^ B16 tumor cells by subcutaneous injection. On day 18 after tumor cell implantation, tumor-infiltrated T cells were isolated by flow sorting and mixed 1:1 with B16 tumor cells, and a total of 5 × 10^5^ cells were injected subcutaneously into new recipient WT mice. Tumor growth was monitored for up to 20 days.

### BMDM antigen presentation and cross-presentation assay

BMDMs with or without LPS treatment were preincubated with 0.5 mg/ml soluble OVA overnight for antigen loading and were then intensively washed with RPMI medium. BMDMs prepared as described above were further cocultured with CFSE-prelabeled OT-I or OT-II splenocytes at a BMDM:T cell ratio of 1:4. The proliferation of OT-I or OT-II T cells was assessed by flow cytometry after 72 h. To further detect the ability of BMDMs to induce MHC-II-specific activation of CD4^+^ T cells, cocultured OT-II CD4^+^ T cells were stimulated with 50 ng/ml PMA and 1 μM ionomycin for 4 h in the presence of 5 μg/ml brefeldin A before being harvested and assessed by flow cytometry to detect CD69 expression and IFN-γ production. At the same time, the content of cytokines in the supernatant was assessed by enzyme-linked immunosorbent assay (ELISA).

### Cytokine measurement

Peripheral blood was collected from WT and *Fats*^*−/−*^ tumor bearing mice 20 days after subcutaneous implantation of B16 cells and allowed to clot for 1–2 h. Serum was separated by centrifugation at 1800 × *g* for 10 min. In other experiments, BMDMs were stimulated with LPS (100 ng/ml) or IL-4 (20 ng/ml) for 24 h or were left untreated. Macrophage supernatants were collected. Cytokine levels in the above-mentioned serum or supernatants were measured using Bio-Plex Pro™ Mouse Cytokine & Chemokine Assay Kits (Bio-Rad) according to the manufacturer’s instructions. Additionally, the supernatants of BMDMs cocultured with OT-II CD4^+^ T cells were collected after 72 h, and mouse IFN-γ and IL-2 ELISA Kits (Multi Sciences) were used to detect the contents of IFN-γ and IL-2 according to the manufacturer’s instructions.

### RNA sequencing

Freshly isolated mouse bone marrow cells from four WT and four *Fats*^*−/−*^ mice were pooled into one replicate set each of WT and *Fats*^*−/−*^ cells and were fully differentiated into macrophages in RPMI medium containing 10% FBS, 1% pen/strep, and 10 ng/ml M-CSF for 6 days. BMDMs were removed from the dishes, and RNA was harvested using a Qiagen Allprep kit. RNA libraries prepared from 1 μg of RNA per sample were prepared for sequencing using standard Illumina protocols. RNA sequencing was performed by GENEWIZ. mRNA profiles were generated by single-end deep sequencing using the Illumina HiSeq 2000 platform.

### Sequence analysis

Sequence files obtained from the Illumina HiSeq platform that passed quality filtering were aligned to the mouse transcriptome (mm9 build) using the Bowtie2 aligner4. Gene-level count summaries were analyzed for significant changes using DESeq. Individual *P* values were adjusted for multiple testing by calculating *q* values as described by Storey using fdrtooltrimmer. For a given gene, the *q* value is the smallest false discovery rate at which the gene can be considered significant. We analyzed biological processes that are defined by the Gene Ontology Consortium. Each gene ontology term defines a set of genes. The entire list of genes, sorted by the *q* value in ascending order, was subjected to a nonparametric variant of gene set enrichment analysis (GSEA), in which the parametric Kolmogorov–Smirnov *P* value was replaced with the exact rank order *P* value. We applied the Bonferroni adjustment to gene set *P* values to correct for the number of gene sets tested. Heatmaps of expression levels were generated using in-house hierarchical clustering software that implements Ward clustering. The colors qualitatively correspond to fold changes.

### Quantitative Real-time PCR

Total RNA was extracted with TRIzol (Invitrogen) from the indicated cells. cDNA was prepared from 2 μg of RNA with a M-MLV Reverse Transcriptase cDNA Synthesis Kit (Invitrogen). SYBR Green-based qPCR was performed using primers for human and mouse *Arg1, Mrc1, Tgfb,* and *Il10* and for mouse *Il1b, Il12a, Il12b, Il6, Ifng, Gmcsf, Tnf, Nos2, Ciita, H2eb1*, and *Ym1*. mRNA levels were normalized to that of *Gapdh* (ΔCt = Ct gene of interest−Ct Gapdh) and are reported as relative mRNA expression (ΔΔCt = 2^−(ΔCt sample−ΔCt control)^) or fold change values. Primer information is listed in Supplementary Table 1.

### Western blot analysis

The whole-cell lysates were prepared using RIPA lysis buffer in the presence of 1% phosphatase inhibitor cocktail and 1 mM phenylmethanesulfonyl fluoride (PMSF). A total of 20–30 μg of protein was electrophoresed on Bio-Rad precast gradient gels and transferred to a PVDF membrane (Millipore, USA). After blocking with 5% non-fat milk at room temperature for 1 h, the membranes were incubated with primary antibodies (1:1000 dilution) overnight at 4 °C. The membrane was then incubated with horseradish peroxidase-conjugated secondary antibody (Cell Signaling Technology, 1:5000) and immunoreactive bands were visualized using the ECL Western Blotting Detection System (Millipore, USA). Antibody information is listed in Supplementary Table 2.

### Co-immunoprecipitation analysis

Co-IP analysis was performed in accordance with standard procedures. Lysates from 1 × 10^7^ cells transfected with the indicated constructs were incubated with 30 µl of Flag-conjugated agarose beads (Sigma-Aldrich, M8823) overnight at 4 °C. Beads containing affinity-bound proteins were washed six times and were then eluted with 50 μl of 2× sample buffer, and the eluates were boiled in a water bath (100 °C) for 10 min. The collected proteins were separated on SDS-polyacrylamide gels and subjected to western blot analysis using anti-myc (Proteintech, 16286-1-AP, 1:4000), anti-HA (Proteintech, 51064-2-AP, 1:3000), and anti-Flag (Sigma-Aldrich, F3165, 1:2000) antibodies.

### Ubiquitination assays

Ubiquitination assays were performed in accordance with the following standard procedures: Myc-tagged IκBα and HA-Ub K48 were overexpressed in HEK293T cells (1 × 10^7^ cells) in the indicated group. Cell lysates were incubated with 30 µl of Myc-conjugated agarose beads (Sigma-Aldrich, E6654) overnight at 4 °C. Beads containing affinity-bound proteins were washed six times and were then eluted with 50 μl of 2× sample buffer, and the eluates were boiled in a water bath (100 °C) for 10 min. The collected proteins were separated on SDS-polyacrylamide gels and subjected to western blot analysis using anti-HA (Proteintech, 51064-2-AP, 1:3000) and anti-Myc (Proteintech, 16286-1-AP, 1:4000) antibodies.

### Immunofluorescence

WT and *Fats*^*−/−*^ BMDMs were grown on chamber slides (BD Discovery Labware, Bedford, MA) in complete RPMI medium for 24 h at 37 °C. Cells were treated with or without LPS at 100 ng/ml for 30 or 60 min. Immediately after LPS treatment, cells were washed with ice-cold PBS twice and were then fixed with 4% paraformaldehyde/PBS for 15 min, permeabilized with 0.2% Triton X-100/PBS for 20 min, and preblocked in 5% BSA/PBS overnight. Slides were then incubated with a rabbit anti-p65 antibody (Cell Signaling Technology, #8242, 1:100) in blocking solution overnight at 4 °C. The next day, slides were washed three times with PBS and incubated with FITC-conjugated goat anti-rabbit IgG (Jackson ImmunoResearch Laboratories, 111-095-003, 1:200) at room temperature in the dark for 1 h. After washing with PBS, slides were counterstained with 4′,6-diamidino-2-phenylindole (DAPI) to identify nuclei. Immunofluorescence images were acquired with an upright fluorescence microscope (OLYMPUS BX51) and analyzed by ImageJ.

### Bioinformatics analysis

Kaplan–Meier plotter (https://kmplot.com)^58^ was used to assess the correlation between *FATS* expression and overall survival of Kidney renal clear cell carcinoma patients, kidney renal papillary cell carcinoma patients, stomach adenocarcinoma patients, uterine corpus endometrial carcinoma patients, and liver hepatocellular carcinoma patients. We analyzed TCGA data for association between *FATS* expression and overall survival of the skin cutaneous melanoma patients. Illumina HiSeq RNAseqV2 mRNA expression and clinical data for 488 skin cutaneous melanoma samples were downloaded from the TCGA data portal. We scored subjects as above (high) or below (low) the median expression for *FATS* genes and compared survival using a log-rank test at 5% significance. Kaplan–Meier curves were plotted for these two groups.

### Statistical analyses

Data from all experiments were statistically analyzed with GraphPad Prism (6.0–8.0). Data were analyzed with two-way ANOVA when comparing two independent groups. Two-tailed unpaired Student’s *t*-test was utilized to analyze the difference between the two groups. Evaluation of survival patterns in tumor-bearing mice was performed using the Kaplan–Meier method, and survival was analyzed with the Mantel–Cox (log-rank) test. Survival was defined as mice with tumors <2500 cm^3^. Data were presented as mean ± s.d. or s.e.m. Differences at *P* < 0.05 were considered statistically significant.

### Reporting summary

Further information on research design is available in the Nature Research Reporting Summary linked to this article.

## Supplementary information

Supplementary Information

Reporting Summary

## Data Availability

The data that support the findings of this study are available within the article and its Supplementary Information or from the corresponding authors on reasonable request, as are unique reagents used in this article. The original fastq data of RNA-seq are not available but the processed excel data are in the manuscript Source Data File. Source data are provided with this paper.

## Source data

Source Data
