# Supplementary material for: Loss of fragile site-associated tumor suppressor promotes antitumor immunity via macrophage polarization
Source: Nat Commun. 2021 Jul 14;12:4300. doi: 10.1038/s41467-021-24610-x (PMC8280123; doi:10.1038/s41467-021-24610-x)
Supplement: Supplementary file 1 — Supplementary Information [file 41467_2021_24610_MOESM1_ESM.pdf]

Loss of fragile site-associated tumor suppressor promotes antitumor immunity via macrophage polarization

Lijuan Zhang[1,#], Kai Zhang[1,#], Jieyou Zhang[1,#], Jinrong Zhu[2], Qing Xi[1], Huafeng Wang[3], Zimu Zhang[1], Yingnan Cheng[1], Guangze Yang[1], Hongkun Liu[1], Xiangdong Guo[1], Dongmei Zhou[1], Zhenyi Xue[1], Yan Li[1], Qi Zhang[4] , Yurong Da[1], Li Liu[5], Zhinan Yin[6], Zhi Yao[1] and Rongxin Zhang[2,7,] *

[1]Key Laboratory of Immune Microenvironment and Diseases (Ministry of Education), Department of Immunology, School of Basic Medical Sciences, Tianjin Medical University, Tianjin 300070, China; [2]Guangdong Province Key Laboratory for Biotechnology Drug Candidates, School of Life Sciences and Biopharmaceutics, Guangdong Pharmaceutical University, Guangzhou 510006, China; [3] School of Life Science, Shanxi Normal University, Linfen, China; [4] Institute of Integrative Medicines for Acute Abdominal Diseases, Nankai Hospital, Tianjin, China; [5] Department of Radiology, The University of Texas Southwestern Medical Center, Dallas, Texas, USA; [6] The First Affiliated Hospital, Biomedical Translation Research Institute and Guangdong Province Key Laboratory of Molecular Immunology and Antibody Engineering, Jinan University, Guangzhou 510632, China; [7] Key Laboratory of Immune Microenvironment and Diseases (Ministry of Education), Tianjin Medical University, Tianjin 300070, China.

[#] These authors contributed equally to this work.

* Email: rxzhang@gdpu.edu.cn.

Supplementary Figures

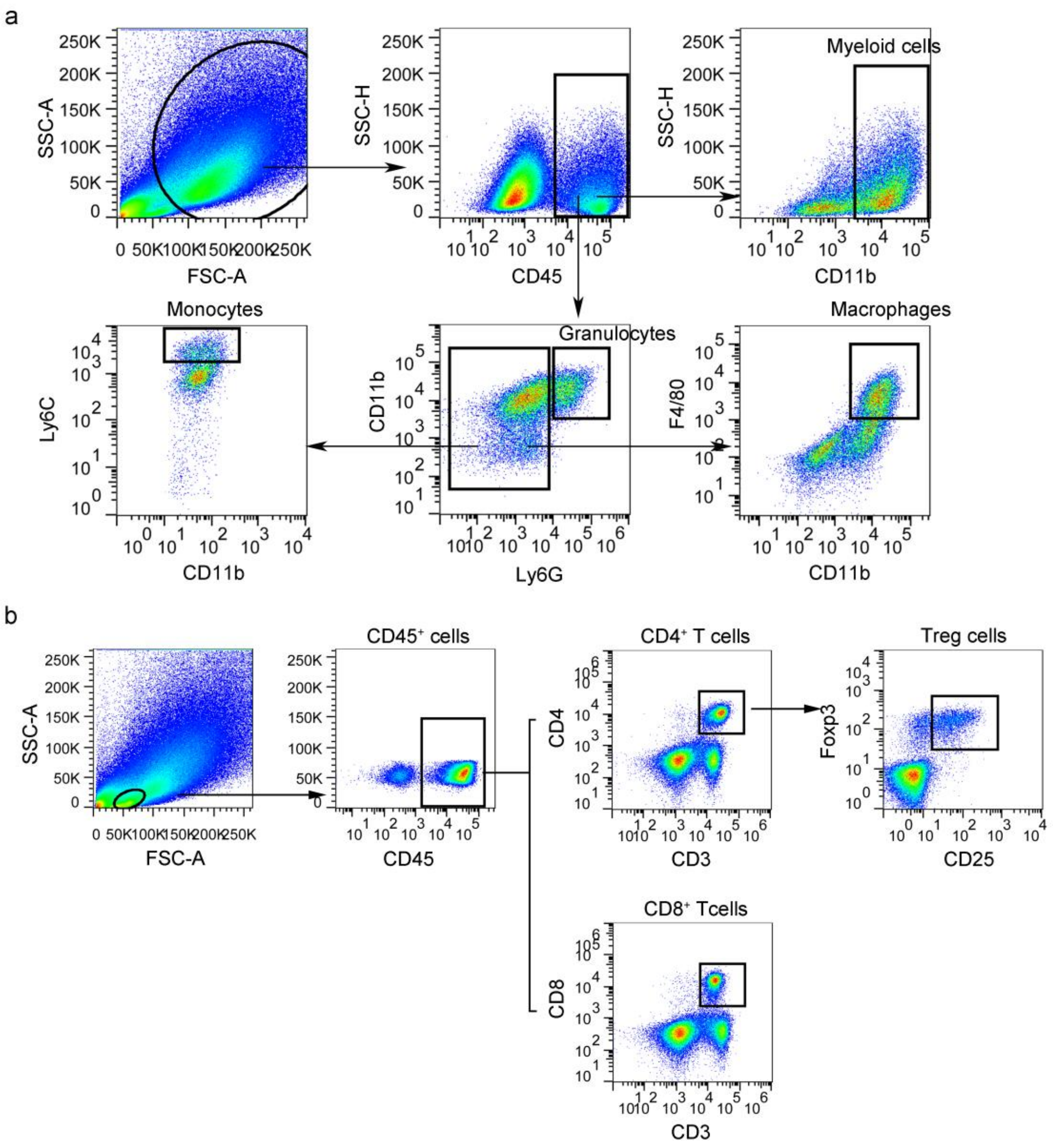

Supplementary Figure 1 Gating strategy to identify myeloid cell and T cell subsets

Single-cell suspension was stained with different fluorophore-conjugated antibodies and analyzed by flow cytometry. **a** Gating strategy to identify myeloid cell subsets. Among single cells, CD45^{+} cells were selected for further analyzed to identify the total myeloid cells (CD11b^{+}), granulocytes (CD11b^{+}Ly6G^{+}), tumor associated macrophages (Ly6G^{-} CD11b^{+} F4/80^{+}, TAMs) and monocytes (Ly6G^{-} CD11b^{+} Ly6c^{hi}) populations. **b** Gating strategy to identify T cell subsets. CD45^{+} cells were selected for further analyzed to identify the CD4^{+} T cells (CD3^{+}CD4^{+}), CD8^{+} T cells (CD3^{+}CD8^{+}) and Treg cells (CD3^{+}CD4^{+}CD25^{+}Foxp3^{+}) populations.

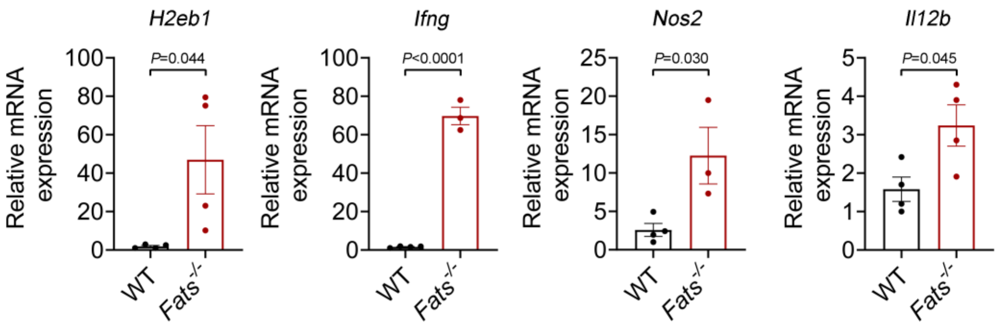

Supplementary Figure 2 FATS deficiency enhanced M1 like makers expression in tumor

B16 melanoma cells (2×10^5) were subcutaneously injected into WT and *Fats*$^{-/-}$ mice. Mice were executed at day 18 after tumor implantation, tumor samples were respectively isolated from WT and *Fats*$^{-/-}$ mice and the expression of *H2eb1, Ifng, Nos2* and *Il12b* was analyzed by RT-PCR. Data are presented as mean ± s.e.m. (*n*=4 for WT, *n*=3-4 for *Fats*$^{-/-}$ mice; two-tailed unpaired Student's *t*-test). Source data are provided as a Source Data file.

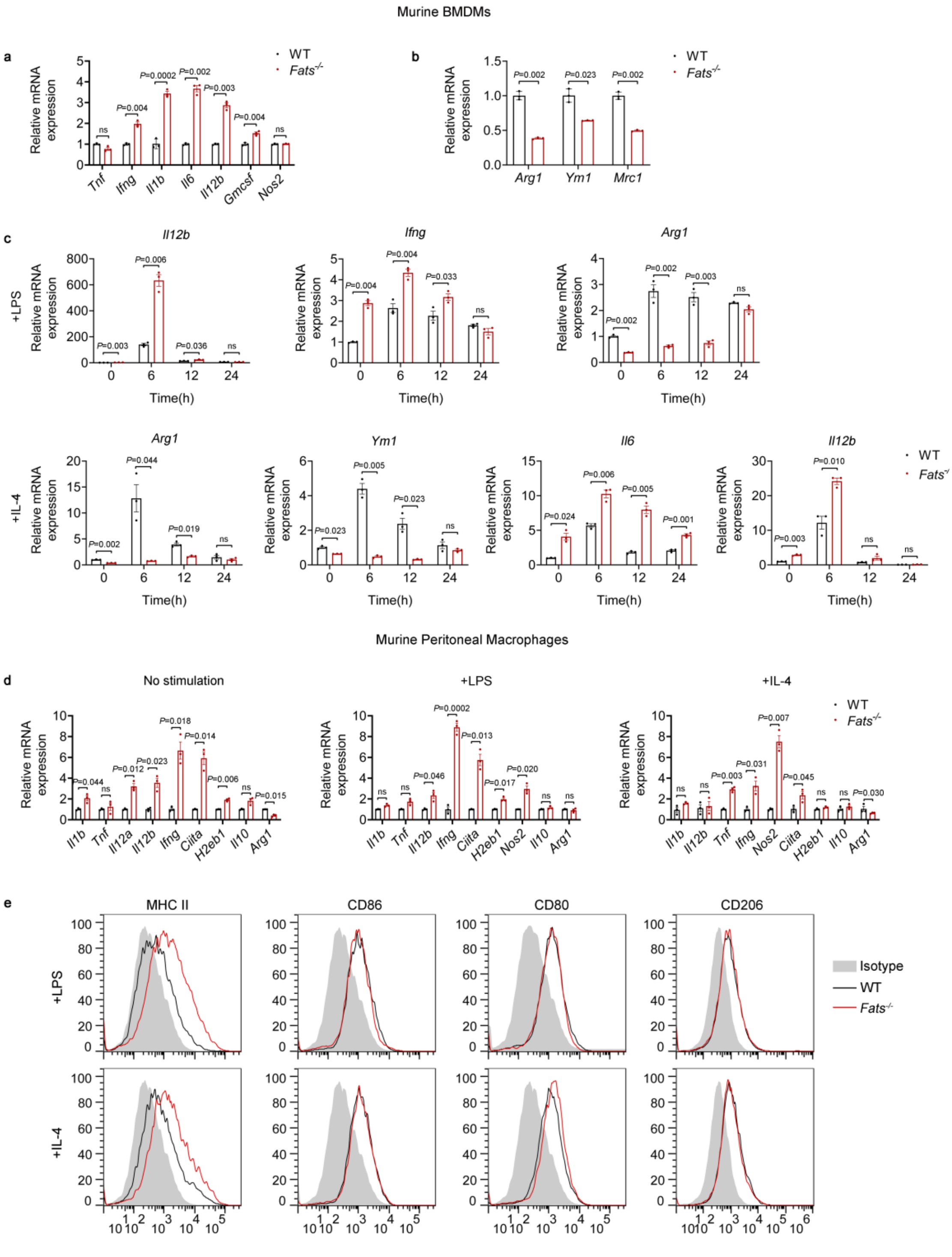

Supplementary Figure 3 FATS deficiency produces an intrinsic bias toward M1 polarization

a, b Relative expression of mRNAs related to inflammatory cytokines and M2 macrophage markers (*Arg1*, *Ym1* and *Mrc1*) in WT and *Fats*$^{-/-}$ BMDMs cultured under M-CSF differentiation conditions (*n*=3 biologically independent samples). **c** Time course of cytokine mRNA expression in WT and

Fats$^{/-}$ BMDMs treated with LPS or IL-4 (*n*=3 biologically independent samples). **d** Relative expression of mRNAs in WT and *Fats*$^{-/-}$ peritoneal macrophages stimulated *in vitro* under basal conditions (No stimulation), pro-inflammatory (LPS) or anti-inflammatory (IL-4) conditions (*n*=3 biologically independent samples). **e** Flow cytometry analysis of MHCII, CD86, CD80 and CD206 expression in WT and *Fats*$^{-/-}$ peritoneal macrophages stimulated with LPS or IL-4. Data are presented as mean ± s.e.m. in **a-d**. *P* values are calculated by two-tailed unpaired Student's *t*-test in **a-d**. ns, not significant. Two independent experiments were carried out with similar results in **e.** Source data are provided as a Source Data file.

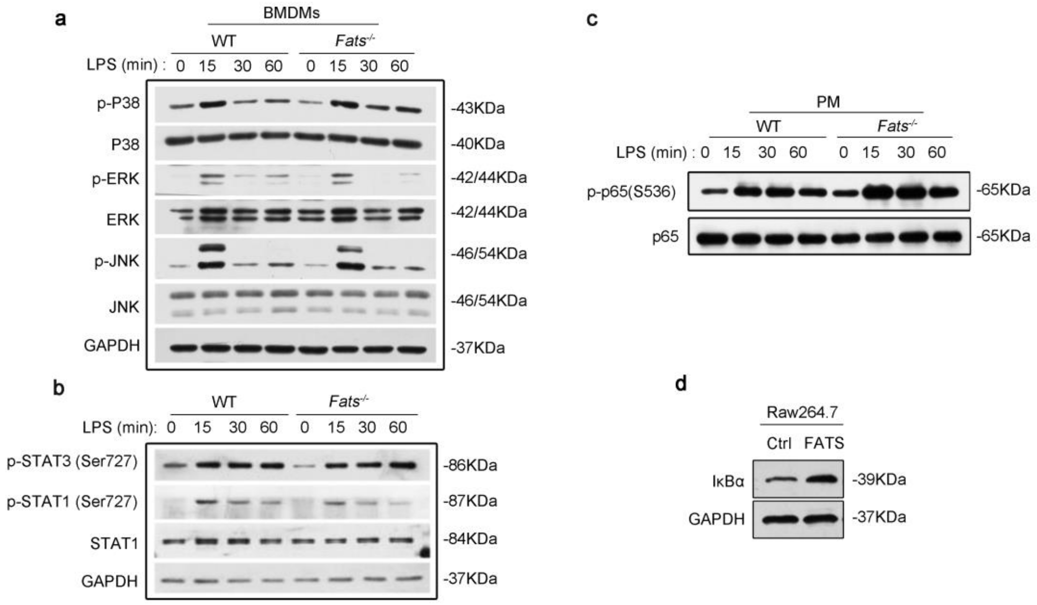

Supplementary Figure 4 FATS inhibits NF-κB activity by promoting the stability of IκBα

a, b The activities of p38/MAPK, ERK, JNK, STAT1 and STAT3 in WT and *Fats*$^{-/-}$ BMDMs after LPS (100 ng/ml) treatment were detected by western blotting. **c** The levels of p-p65 and p65 in WT and *Fats*$^{-/-}$ primary peritoneal macrophages (PM) after LPS (100 ng/ml) treatment were detected by western blotting. **d** The levels of IκBα in RAW264.7 macrophages infected with lentiviral vector encoding murine FATS (FATS) or enmpty vector (Ctrl). Two independent experiments were carried out with similar results in **a-d.** Source data are provided as a Source Data file.

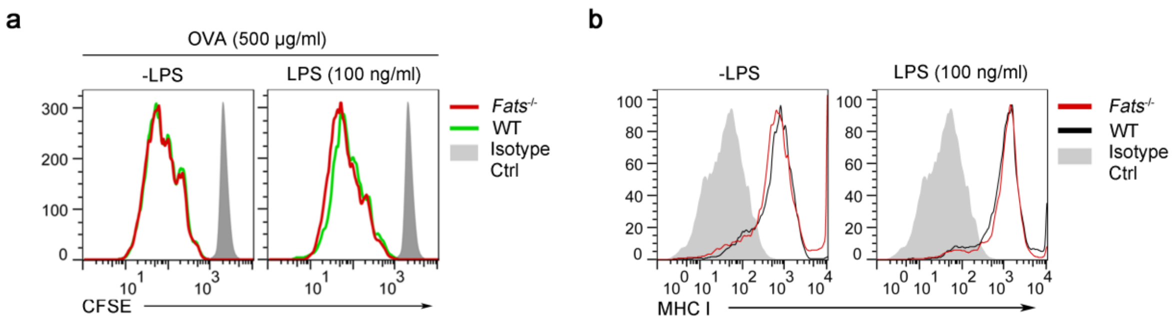

Supplementary Figure 5 FATS deficiency does not alter macrophage antigen cross-presentation ability

a BMDMs from WT or *Fats*$^{-/-}$ mice were stimulated with or without LPS, preincubated with soluble ovalbumin (OVA) overnight and cocultured with CFSE-labeled CD8^{+} OT-I T cells. The proliferation of CD8^{+} OT-I T cells was analyzed by flow cytometry after 72h. **b** Flow cytometric analysis the expression of MHCI in WT and *Fats*$^{-/-}$ BMDMs stimulated with or without LPS *in vitro*. Two independent experiments were carried out with similar results in **a** and **b.** Source data are provided as a Source Data file.

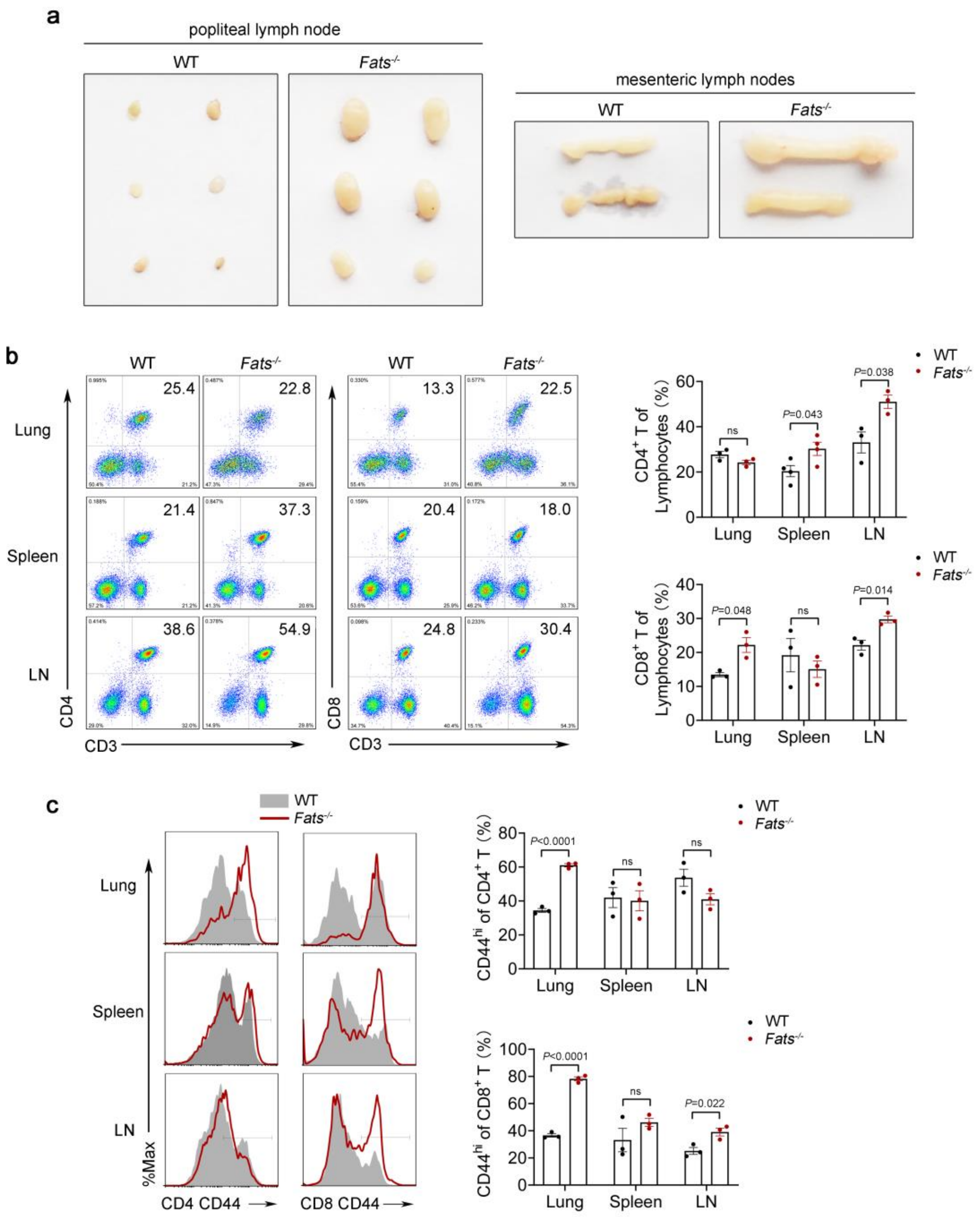

Supplementary Figure 6 FATS deficiency influences the frequency and activation of T cells

a-c B16 melanoma cells (5×10^5) were intravenously injected into WT and *Fats*$^{-/-}$ mice to induce melanoma with lung metastasis. On day 20, spleen, lymph node and lung tissue were isolated. **a** Representative pictures of popliteal lymph nodes (pLNs) and mesenteric lymph nodes (mLNs) (*n*=3 mice per group). **b** Flow cytometric analysis and quantification of the frequency of CD4$^+$ T and CD8$^+$ T cells in spleen, draining lymph node and lung (*n*=3-4 mice per group). **c** Flow cytometric analysis and quantification of surface CD44 expression in CD4$^+$ T and CD8$^+$ T cells in spleen, draining lymph node and lung (*n*=3 mice per group). Data are presented as mean ± s.e.m. in **b** and **c**. *P* values are

calculated by two-tailed unpaired Student's *t*-test in **b** and **c**. ns, not significant. Source data are provided as a Source Data file.

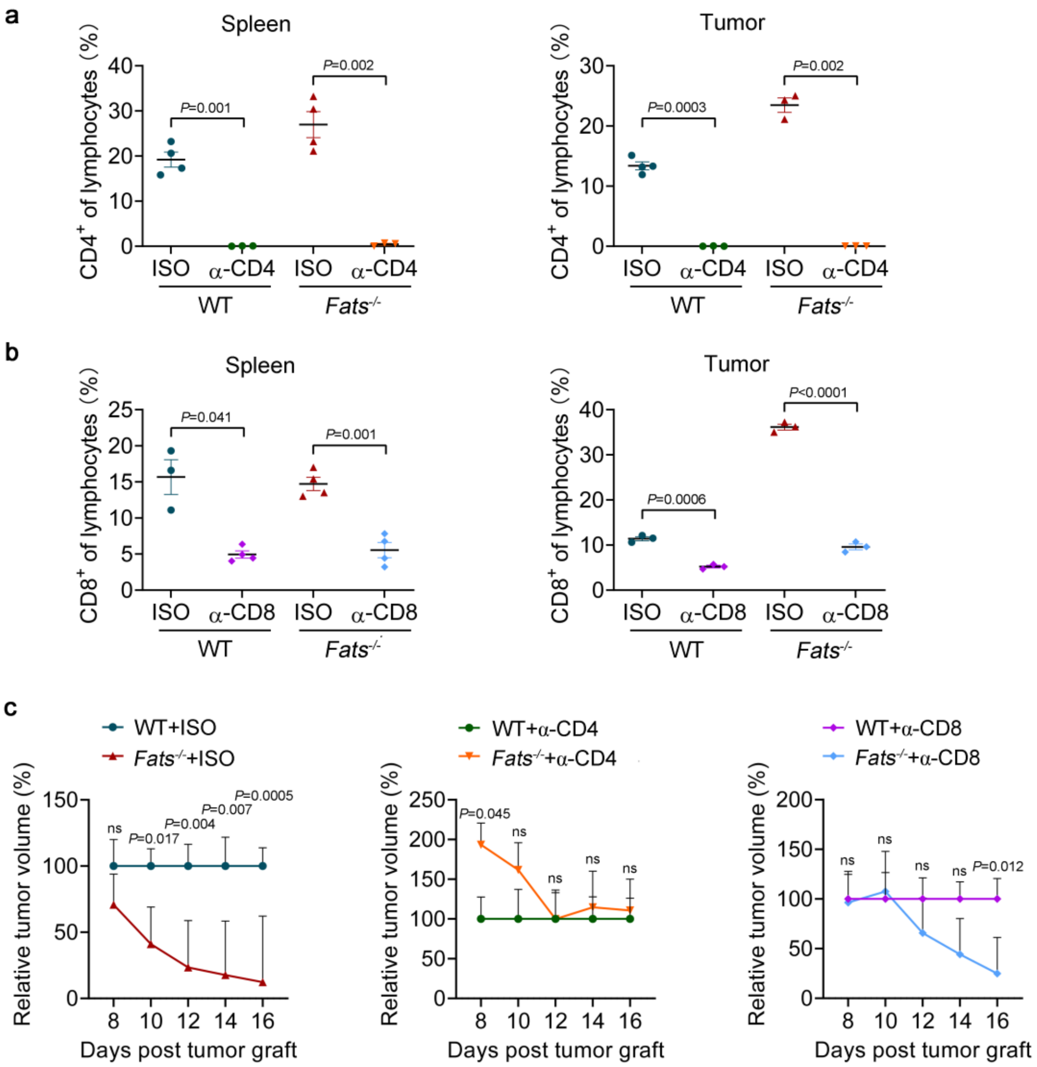

Supplementary Figure 7 *Fats*$^{-/-}$ Macrophage requires T cells for inhibiting tumor growth

a Flow cytometric quantification of CD4^{+} T cells in spleen and tumors in WT and *Fats*$^{-/-}$ mice after the treatment with depleting antibodies against CD4 (*n*=3-4 mice per group). **b** Flow cytometric quantification of CD8^{+} T cells in spleen and tumors in WT and *Fats*$^{-/-}$ mice after the treatment with depleting antibodies against CD8 (*n*=3-4 mice per group). **c** The relative tumor volume in WT and *Fats*$^{-/-}$ mice received depleting antibodies against CD4 or CD8a during tumor growth (*n*=6 mice per group). Data are presented as mean ± s.e.m. in **a, b** and **c**. *P* values are calculated by two-tailed unpaired Student's *t*-test in **a-c**. ns, not significant. Source data are provided as a Source Data file.

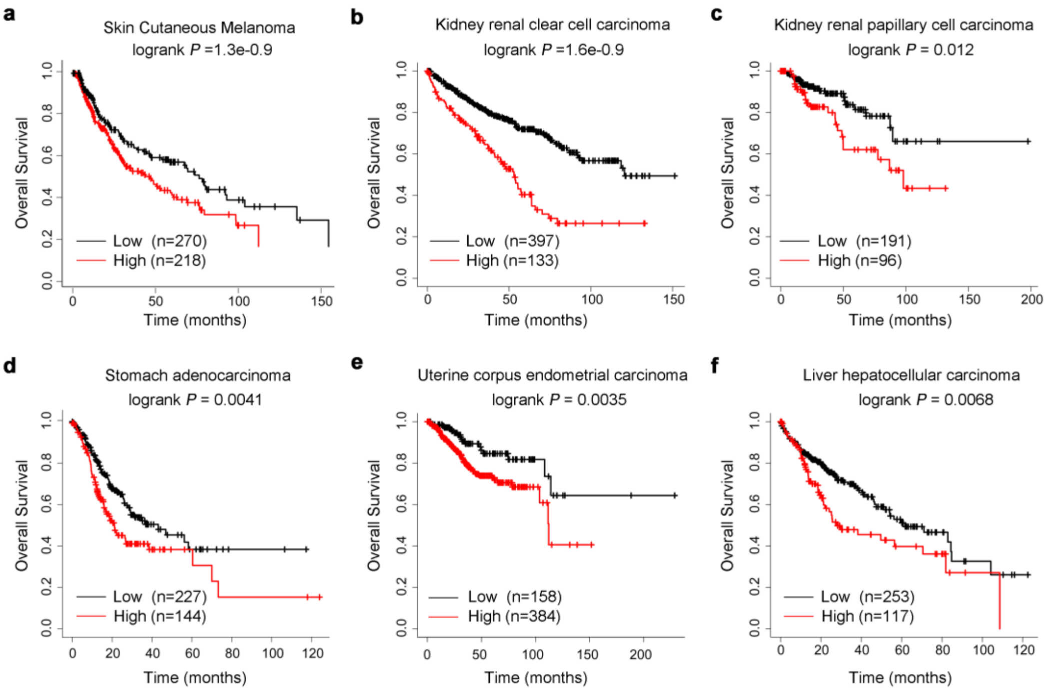

Supplementary Figure 8 *FATS* expression signatures predict survival in cancer patients

a-f Association of *FATS* expression with survival in Skin Cutaneous Melanoma patients, Kidney renal clear cell carcinoma patients, Kidney renal papillary cell carcinoma patients, Stomach adenocarcinoma patients, Uterine corpus endometrial carcinoma patients and Liver hepatocellular carcinoma patients. Primary tumor samples with *FATS* expression data were scored as above or below the median expression level, and tested for association with patient survival using a logrank test in **a-f**.

Supplementary Table 1

The list of primers

Gene	Species	Forward Primer	Reverse Primer
Tnf	Mouse	GAGGCCAAGCCCTGGTATG	CGGGCCGATTGATCTCAGC
Il12a	Mouse	AGACATCACACGGGACCAAAC	CCAGGCAACTCTCGTTCTTGT
Il12b	Mouse	CTGGAGCACTCCCCATTCCTA	GCAGACATTCCCGCCTTTG
Il6	Mouse	CTGCAAGAGACTTCCATCCAG	AGTGGTATAGACAGGTCTGTTGG
Ifng	Mouse	ACAGCAAGGCGAAAAAGGATG	TGGTGGACCACTCGGATGA
H2eb1	Mouse	GCGGAGAGTTGAGCCTACG	AGGCCCGTGGACACAATTC
Ciita	Mouse	GGAGGAGATCGAACTCAGCTC	GTTCCGCAATGTTGGCATAGG
Gmcsf	Mouse	GGCCTTGGAAGCATGTAGAGG	GGAGAACTCGTTAGAGACGACTT
Nos2	Mouse	GTTCTCAGCCCAACAATACAAGA	GTGGACGGGTCGATGTCAC
Il1b	Mouse	GAAATGCCACCTTTTGACAGTG	TGGATGCTCTCATCAGGACAG
Il10	Mouse	GCTGGACAACATACTGCTAACC	ATTTCCGATAAGGCTTGGCAA
Arg1	Mouse	CTCCAAGCCAAAGTCCTTAGAG	GGAGCTGTCATTAGGGACATCA
Mrc1	Mouse	CTCTGTTCAGCTATTGGACGC	TGGCACTCCCAAACATAATTTGA
Ym1	Mouse	CAGGTCTGGCAATTCTTCTGAA	GTCTTGCTCATGTGTGTAAGTGA
Tgfb	Mouse	CCACCTGCAAGACCATCGAC	CTGGCGAGCCTTAGTTTGGAC
Gapdh	Mouse	AGGTCGGTGTGAACGGATTTG	GGGGTCGTTGATGGCAACA
Fats	Mouse	TGCACAGGCGATTTGCTTTAT	ACCTTTGCCTAGATTGTCCCC
FATS	Human	TGTGCCATTGCTCAGTCTCG	TCTGTGAATGTTGACTCCGCT
ARG1	Human	GTGGAAACTTGCATGGACAAC	AATCCTGGCACATCGGGAATC
MRC1	Human	GGGTTGCTATCACTCTCTATGC	TTTCTTGTCTGTTGCCGTAGTT
TGFB	Human	GGCCAGATCCTGTCCAAGC	GTGGGTTTCCACCATTAGCAC
IL10	Human	GACTTTAAGGGTTACCTGGGTTG	TCACATGCGCCTTGATGTCTG
GAPDH	Human	ACAACTTTGGTATCGTGGAAGG	GCCATCACGCCACAGTTTC

Supplementary Table 2

Antibodies used in this study

Antibodies	Supplier	Application	Catalog number	Clone name	Dilution
APC-anti-mouse CD45	eBioscience	Flow	17-0451-82	30-F11	1:200
FITC-anti-mouse CD45	eBioscience	Flow	11-0451-81	30-F11	1:200
FITC-anti-mouse CD11b	eBioscience	Flow	11-0112-81	M1/70	1:200
PE-anti-mouse Ly6C	eBioscience	Flow	12-5932-82	HK1.4	1:200
PE-Cy7-anti-mouse Ly6G	eBioscience	Flow	25-5931-81	RB6-8C5	1:200
APC-anti-mouse CD11c	eBioscience	Flow	17-0114-81	N418	1:200
PE-anti-mouse MHC Class II (I-A/I-E)	eBioscience	Flow	12-5321-82	M5/114.15.2	1:200
FITC-anti-mouse CD3ε	eBioscience	Flow	11-0031-82	145-2C11	1:200
APC-anti-mouse CD8a	eBioscience	Flow	17-0081-81	53-6.7	1:200
PE-anti-mouse CD44	eBioscience	Flow	12-0441-81	IM7	1:200
PE-anti-mouse CD86	eBioscience	Flow	12-0861-81	PO3.1	1:200
PE-anti-mouse FoxP3	eBioscience	Flow	12-4771-82	NRRF-30	1:200
PE-anti-mouse IFNγ	eBioscience	Flow	12-7311-82	XMG1.2	1:200
PE-anti-mouse TNF-α	eBioscience	Flow	12-7321-82	MP6-XT22	1:200
PE-anti-human HLA-DR	eBioscience	Flow	12-9956-41	LN3	1:200
PE-anti-human CD86	eBioscience	Flow	12-0869-41	IT2.2	1:200
FITC-anti-mouse IgG2b Isotype	eBioscience	Flow	11-4732-42	eBMG2b	1:200
PE-anti-mouse IgG2b Isotype	eBioscience	Flow	12-4732-42	eBMG2b	1:200
APC-anti-mouse IgG2b Isotype	eBioscience	Flow	17-4732-42	eBMG2b	1:200
PE-anti-mouse H-2K^b (MHC class I)	Biolegend	Flow	116507	AF6-88.5	1:200
FITC-anti-mouse CD206	Biolegend	Flow	141703	C068C2	1:200
APC-anti-mouse CD4	Biolegend	Flow	100516	RM4-5	1:200
FITC-anti-mouse CD25	Biolegend	Flow	101907	3C7	1:200
FITC-anti-human/mouse Granzyme B	Biolegend	Flow	515403	GB11	1:200
PE-anti-mouse CD11b	Sungene	Flow	M10117-09B	M1/70	1:100
APC-anti-mouse F4/80	Sungene	Flow	M100F1-11A	BM8	1:100
PE-anti-mouse CD80	Sungene	Flow	M10801-09B	16-10A1	1:100
PE-anti-mouse PD-1	Sungene	Flow	M12791-09B	J43	1:100
anti-CD3	Invitrogen	T-Cell activation	16-0031-86	145-2C11	1:200
anti-CD28	Invitrogen	T-Cell activation	16-0281-85	37.51	1:500
FITC-conjugated goat anti-rabbit IgG	Jackson ImmunoResearch Laboratories	IF	111-095-003		1:200
anti-NF-κB p65	Cell Signaling Technology	IF	8242	D14E12	1:100
anti-NF-κB p65	Cell Signaling Technology	western blot	8242	D14E12	1:1000
anti-Phospho-NF-κB p65 (Ser536)	Cell Signaling Technology	western blot	3033	93H1	1:1000
anti-p38	Cell Signaling Technology	western blot	8690	D13E1	1:1000

anti-Phospho-p38 (Thr180/Tyr182)	Cell Signaling Technology	western blot	4511	D3F9	1:1000
anti-p44/42 MAPK (Erk1/2)	Cell Signaling Technology	western blot	4695	137F5	1:1000
anti-JNK2	Cell Signaling Technology	western blot	9258	56G8	1:1000
anti-Phospho-SAPK/JNK (Thr183/Tyr185)	Cell Signaling Technology	western blot	4668	81E11	1:1000
anti-Stat1	Cell Signaling Technology	western blot	14994	D1K9Y	1:1000
anti-Phospho-Stat3 (Ser727)	Cell Signaling Technology	western blot	49081	D8C2Z	1:1000
anti-Phospho-IκBα (Ser32)	Cell Signaling Technology	western blot	2859	14D4	1:1000
anti-IκBα	Cell Signaling Technology	western blot	4814	L35A5	1:1000
anti-IKKβ	Cell Signaling Technology	western blot	8943	D30C6	1:1000
anti-Phospho-IKKα/β (Ser176/180)	Cell Signaling Technology	western blot	2697	16A6	1:1000
anti-GAPDH	Cell Signaling Technology	western blot	2118	14C10	1:1000
anti-Phospho-p44/42 MAPK (Erk1/2) (Thr202/Tyr204)	Cell Signaling Technology	western blot	9101		1:1000
anti-rabbit IgG, HRP-linked Antibody	Cell Signaling Technology	western blot	7074		1:5000
anti-Phospho-STAT1-S727	ABclonal Technology	western blot	AP0453		1:2000
anti-Myc	Proteintech	western blot	16286-1-AP		1:4000
anti-HA	Proteintech	western blot	51064-2-AP		1:3000
anti-Flag	Sigma-Aldrich	western blot	F3165		1:2000
