# Supplementary material for: Loss of fragile site-associated tumor suppressor promotes antitumor immunity via macrophage polarization
Source: Nat Commun. 2021 Jul 14;12:4300. doi: 10.1038/s41467-021-24610-x (PMC8280123; doi:10.1038/s41467-021-24610-x)
Supplement: Supplementary file 2 — Reporting Summary [file 41467_2021_24610_MOESM2_ESM.pdf]

nature research

Corresponding author(s): Rongxin Zhang

Last updated by author(s): Jun 8, 2021

Reporting Summary

Nature Research wishes to improve the reproducibility of the work that we publish. This form provides structure for consistency and transparency in reporting. For further information on Nature Research policies, see our Editorial Policies and the Editorial Policy Checklist.

Statistics

For all statistical analyses, confirm that the following items are present in the figure legend, table legend, main text, or Methods section.

n/a	Confirmed	
☐	☒	The exact sample size (*n*) for each experimental group/condition, given as a discrete number and unit of measurement
☐	☒	A statement on whether measurements were taken from distinct samples or whether the same sample was measured repeatedly
☐	☒	The statistical test(s) used AND whether they are one- or two-sided<br>*Only common tests should be described solely by name; describe more complex techniques in the Methods section.*
☒	☐	A description of all covariates tested
☒	☐	A description of any assumptions or corrections, such as tests of normality and adjustment for multiple comparisons
☐	☒	A full description of the statistical parameters including central tendency (e.g. means) or other basic estimates (e.g. regression coefficient) AND variation (e.g. standard deviation) or associated estimates of uncertainty (e.g. confidence intervals)
☐	☒	For null hypothesis testing, the test statistic (e.g. *F*, *t*, *r*) with confidence intervals, effect sizes, degrees of freedom and *P* value noted<br>*Give P values as exact values whenever suitable.*
☒	☐	For Bayesian analysis, information on the choice of priors and Markov chain Monte Carlo settings
☒	☐	For hierarchical and complex designs, identification of the appropriate level for tests and full reporting of outcomes
☒	☐	Estimates of effect sizes (e.g. Cohen's *d*, Pearson's *r*), indicating how they were calculated

Our web collection on statistics for biologists contains articles on many of the points above.

Software and code

Policy information about availability of computer code

Data collection	qRT-PCR data were collected by the ABI 7500 Fast Dx instruments' Sequence Detection Software v2.0.5. Western blot images were manual exposure and were scaned using HP Scanjet F4050. For immunofluorescence, fluorescent images were acquired using an upright fluorescence microscope (OLYMPUS BX51) and analyzed by ImageJ (1.52a). Flow cytometry was performed on a BD FACSCanto II and data were analyzed using Flowjo 7.6.1. or Flowjo V10.
Data analysis	Microsoft Excel 2007 and GraphPad Prism (6.0-8.0) for Statistical analysis and graph plotting. Web site: Kaplan-Meire plotter (https://kmplot.com) was used to analyze the correlation between FATS expression and overall survival of Kidney renal clear cell carcinoma patients, Kidney renal papillary cell carcinoma patients, Stomach adenocarcinoma patients, Uterine corpus endometrial carcinoma patients and Liver hepatocellular carcinoma patients. TCGA database was used to assess the correlation between FATS expression and Skin Cutaneous Melanoma patients. R (version:4.0.1) was used for analysis.

For manuscripts utilizing custom algorithms or software that are central to the research but not yet described in published literature, software must be made available to editors and reviewers. We strongly encourage code deposition in a community repository (e.g. GitHub). See the Nature Research guidelines for submitting code & software for further information.

Data

Policy information about availability of data

All manuscripts must include a data availability statement. This statement should provide the following information, where applicable:

- Accession codes, unique identifiers, or web links for publicly available datasets
- A list of figures that have associated raw data
- A description of any restrictions on data availability

The source data are provided as a source data file.

All other data that support the findings of this study are available from the corresponding author (R.Z.). upon reasonable request.

Field-specific reporting

Please select the one below that is the best fit for your research. If you are not sure, read the appropriate sections before making your selection.

- [x] Life sciences
- [ ] Behavioural & social sciences
- [ ] Ecological, evolutionary & environmental sciences

For a reference copy of the document with all sections, see nature.com/documents/nr-reporting-summary-flat.pdf

Life sciences study design

All studies must disclose on these points even when the disclosure is negative.

Sample size	For the animal experiments, sample size was determined based on pilot studies and previous experimental experiences. For cell and biochemical data, we aimed to collect data from three biological replicates when possible.
Data exclusions	No data were excluded from the analyses.
Replication	All attempts at replication were successful. How many times each experiment was performed and which statistical analysis was used is indicated in the figure legends.
Randomization	All samples were randomly allocated into experimental groups.
Blinding	The investigators were not blinded to the group allocation during data collection and/or data analysis. Data reported for mouse experiments are not subjective but rather based on quantitative flow cytometry. For other experiments for human samples or cells, blinding were not performed due to feasibility.

Reporting for specific materials, systems and methods

We require information from authors about some types of materials, experimental systems and methods used in many studies. Here, indicate whether each material, system or method listed is relevant to your study. If you are not sure if a list item applies to your research, read the appropriate section before selecting a response.

Materials & experimental systems

n/a	Involved in the study	
[ ]	[x]	Antibodies
[ ]	[x]	Eukaryotic cell lines
[x]	[ ]	Palaeontology and archaeology
[ ]	[x]	Animals and other organisms
[ ]	[x]	Human research participants
[x]	[ ]	Clinical data
[x]	[ ]	Dual use research of concern

Methods

n/a	Involved in the study	
[x]	[ ]	ChIP-seq
[ ]	[x]	Flow cytometry
[x]	[ ]	MRI-based neuroimaging

Antibodies

Antibodies used	Information of antibodies used in this study were provided in supplementary Table 2. The supplier name, application, catalog number, clone name and the dilution used for each antibody were described in supplementary Table 2.
Validation	All commercially available antibodies were validated by vendors. Validation statements are provided on the manufacture's website. We examined primary antibodies according to manuals, and got similar results with validation results on manufacturer's website or relevant citations.

Eukaryotic cell lines

Policy information about cell lines

Cell line source(s)	The murine cancer cell lines including melanoma (B16), pancreatic cancer (H7), RAW264.7 and HEK293T cells were obtained from ATCC biobank.
Authentication	Murine melanoma (B16), pancreatic cancer (H7), and RAW264.7 cells and HEK293T cells were obtained from ATCC (2016) and authenticated using short tandem repeat profiling.
Mycoplasma contamination	all cell lines were tested negative for mycoplasma contamination

Commonly misidentified lines (See ICLAC register)	No commonly misidentified cell lines were used in the study.

Animals and other organisms

Policy information about studies involving animals; ARRIVE guidelines recommended for reporting animal research

Laboratory animals	Female C57BL/6 mice (six-eight weeks old), FATS knock-out mice (Fats-/-), OT-I and OT-II TCR transgenic mice were maintained under SPF conditions in a controlled environment of 20-22, with a 12/12h light/dark cycle, 50-70% humidity, and food and water provided ad libitum.
Wild animals	The study did not involve wild animals.
Field-collected samples	The study did not involve samples collected from the field.
Ethics oversight	All the animal experiments were carried out in the animal unit, Tianjin Medical University (Tianjin, China) according to procedures authorized and specifically approved by the institutional ethical committee (Permit Number: SYXK 2009-0001).

Note that full information on the approval of the study protocol must also be provided in the manuscript.

Human research participants

Policy information about studies involving human research participants

Population characteristics	Fresh human buffy coat blood from healthy adults with type A blood were purchased from Tianjin Blood Center (Tianjin, China).
Recruitment	Participants were recruited from the Tianjin Blood Center (Tianjin, China). No selection bias was observed.
Ethics oversight	We have complied with all relevant ethical regulations and were given guidelines for study procedures from Tianjin Blood Center (Tianjin, China) and Tianjin medical university (Tianjin, China). Fresh human buffy coat blood was obtained from Tianjin Blood Center. Informed consent was not necessary when we purchased buffy coat from the blood center.

Note that full information on the approval of the study protocol must also be provided in the manuscript.

Flow Cytometry

Plots

Confirm that:

- [x] The axis labels state the marker and fluorochrome used (e.g. CD4-FITC).
- [x] The axis scales are clearly visible. Include numbers along axes only for bottom left plot of group (a 'group' is an analysis of identical markers).
- [x] All plots are contour plots with outliers or pseudocolor plots.
- [x] A numerical value for number of cells or percentage (with statistics) is provided.

Methodology

Sample preparation	Samples were prepared as described in the methods section. Animals were culled according to procedures authorized and specifically approved by the institutional ethical committee (permit SYXK 2009-0001). Single-cell suspensions derived from spleens and draining lymph nodes were prepared by mechanical disruption, filtered through a 70-µm cell strainer (BD Biosciences). Red blood cells were solubilized with red cell lysis buffer (Solarbio). For tumor tissues, tumors were isolated, minced into small pieces and digested with 0.05 mg/ml each of type-IV collagenase, hyaluronidase and DNase I (Sigma, USA) for 30 min at 37°C. Single-cell suspensions were obtained by grinding the digested tissues and filtering them through a 70-µm cell strainer. Red blood cells were solubilized with red cell lysis buffer (Solarbio). Tumor-infiltrating mononuclear cells were isolated using Ficoll density gradient centrifugation and analyzed by flow cytometry. Live/Dead stains were used to exclude dead cells.Peripheral blood mononuclear cells were isolated with human Lymphoprep solution (Axis-shield PoC AS, Oslo,Norway) per the manufacturer's instruction.Single cell suspensions collected for further analysis by flow cytometry.
Instrument	The instrument used for data collection were BD FACS Canto II. Cat No:338960
Software	All the flow cytometry data were collected using BD FACS Canto II and the data were processed using FlowJo software (version 7.6.1 or version10).
Cell population abundance	For all cell populations analysed, abundances are indicated in the figure plots. Myeloid cells were stained with anti-CD11b-FITC (M1/70) antibodies for flow sorting on a FACS Aria II. Tumor-associated macrophages (TAM) were stained with anti-CD11b-FITC (M1/70) and anti-F4/80-APC (BM8) antibodies for flow sorting on a FACS Aria II. Tumor-infiltrated CD3+T cells were isolated by flow sorting on a FACS Aria II. The purity of flow-sorted populations was above 90%. In other experiment, CD3+ Tcells were purified using anti-CD3 microbeads (Miltenyi Biotech) and the purity of CD3+ T cells, examined by flow cytometry, was above 90%.

Gating strategy	All gate strategies captured cells by FSC vs SSC area, single cells by FSC height versus area. Gating strategies beyond this differed by experiment. Single-cell suspension was stained with different fluorophore-conjugated antibodies and analyzed by flow cytometry. Among single cells, CD45+ cells was selected for further analyzed to identify the total myeloid cells (CD11b+), granulocytes (CD11b+Ly6G+), tumor associated macrophages (Ly6G- CD11b+ F4/80+, TAMs), monocytes (Ly6G- CD11b+ Ly6chi), CD4+ T cells (CD3+CD4+), CD8+ T cells (CD3+CD8+), Treg (CD3+CD4+CD25+Foxp3+) populations. Dead cells were excluded on the basis of forward and side scatter.

☒ Tick this box to confirm that a figure exemplifying the gating strategy is provided in the Supplementary Information.
