# Supplementary material for: Loss of fragile site-associated tumor suppressor promotes antitumor immunity via macrophage polarization
Source: Nat Commun. 2021 Jul 14;12:4300. doi: 10.1038/s41467-021-24610-x (PMC8280123; doi:10.1038/s41467-021-24610-x)
Supplement: Supplementary file 3 — Source Data [file 41467_2021_24610_MOESM3_ESM.zip › sounce data/western blot.pptx]

## Slide 1
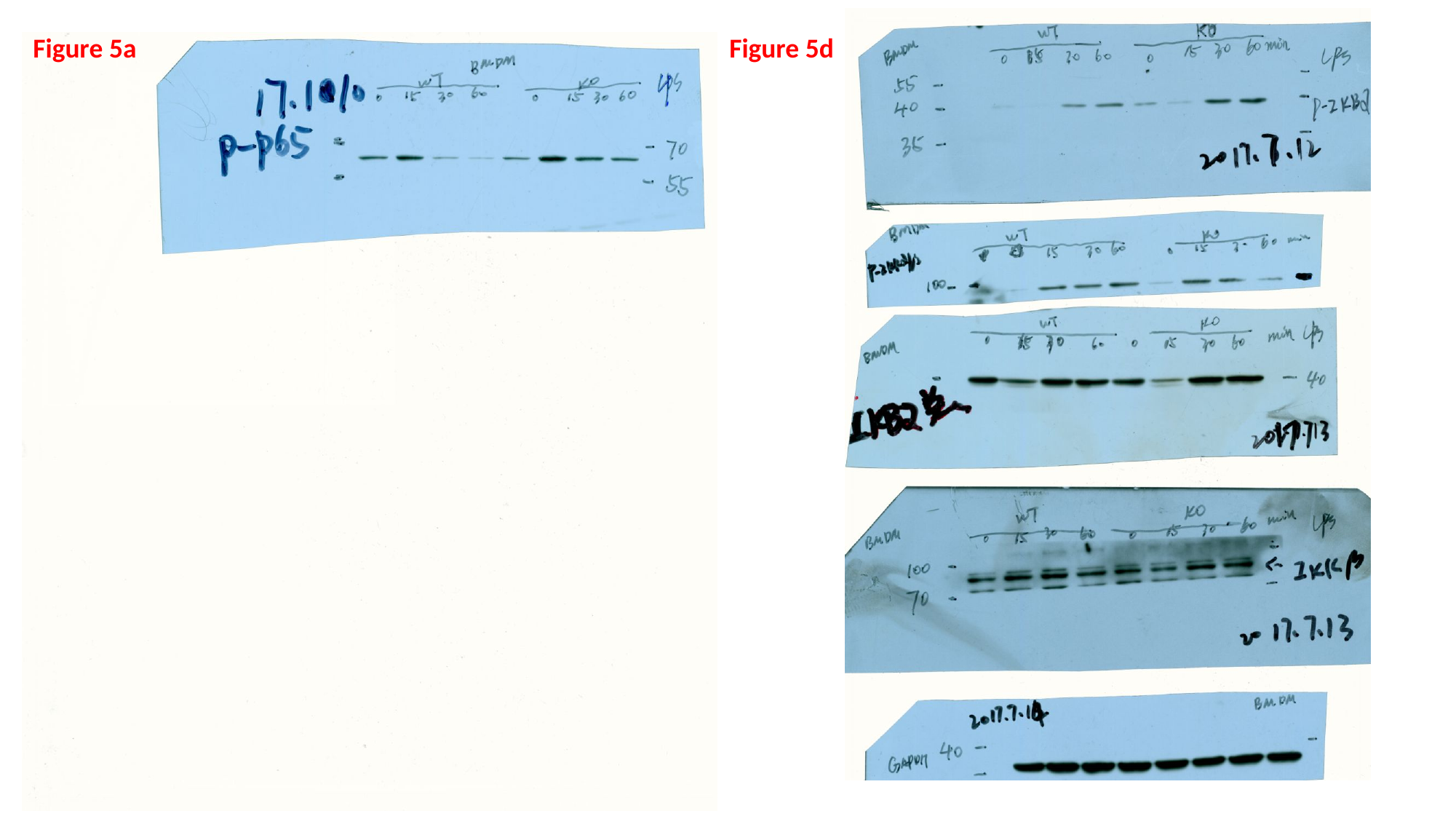

Figure 5a
Figure 5d

## Slide 2
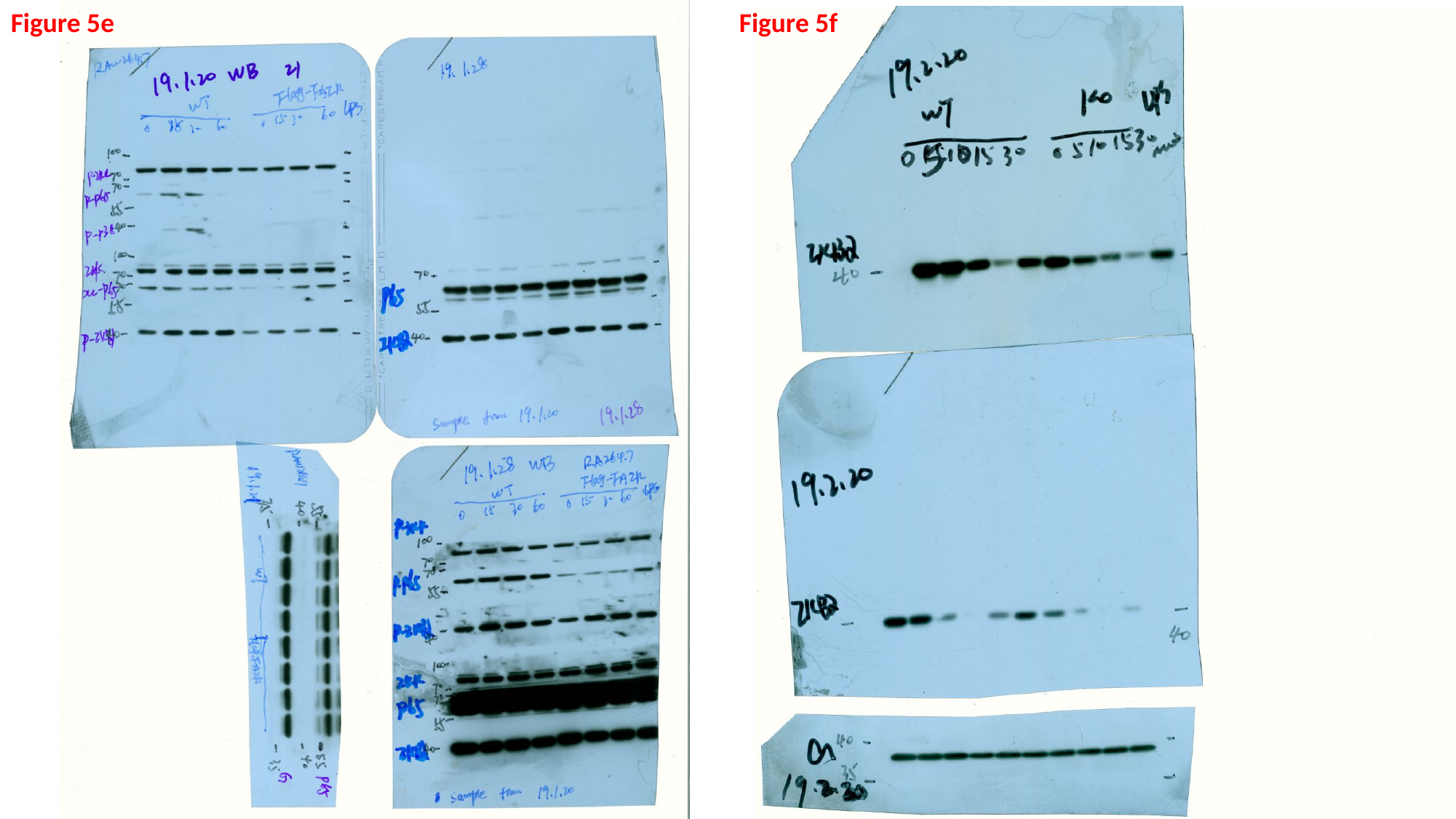

Figure 5e
Figure 5f

## Slide 3
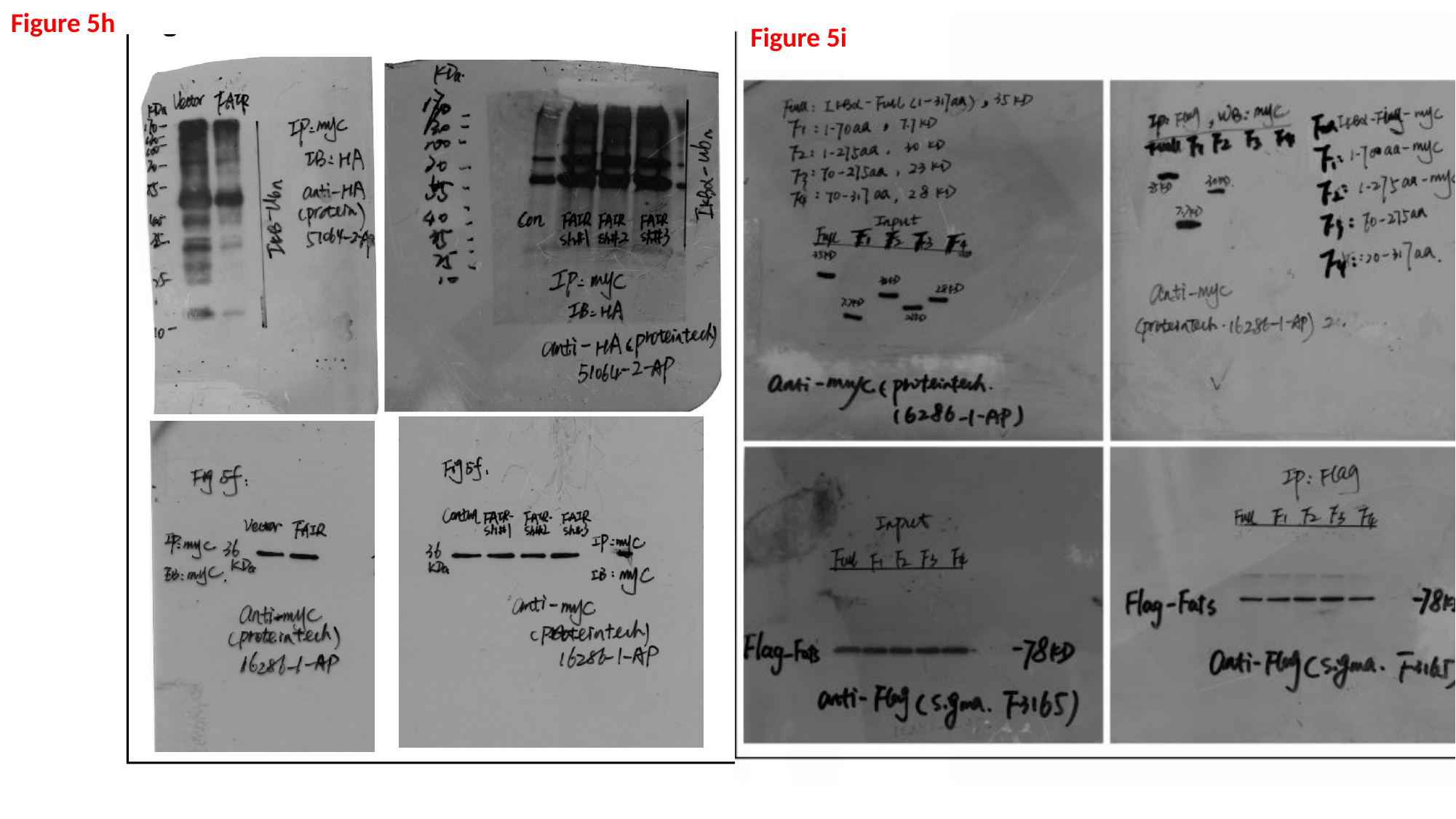

Figure 5h
Figure 5i

## Slide 4
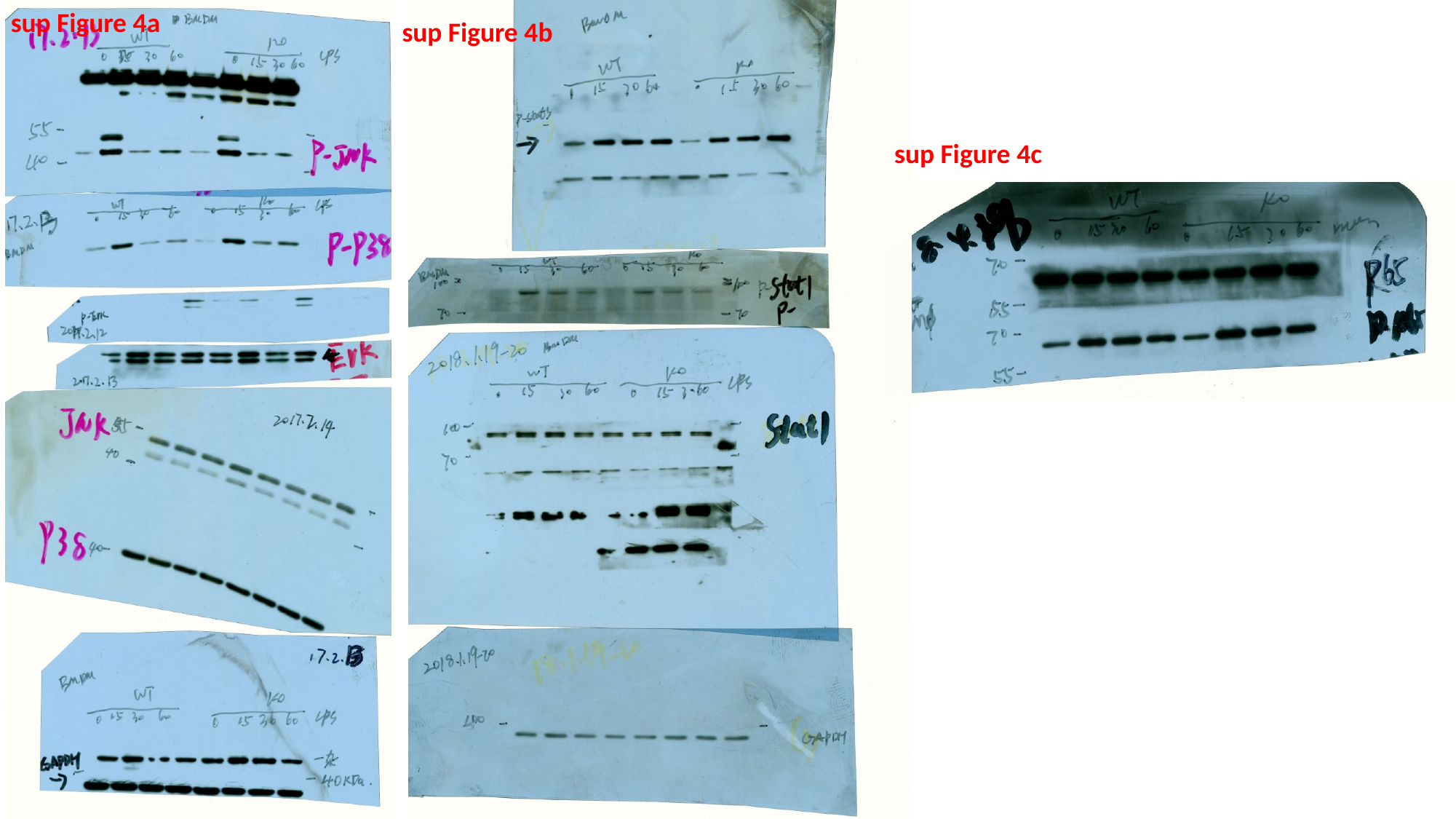

sup Figure 4a
sup Figure 4b
sup Figure 4c
