# Supplementary figures and images for: Loss of fragile site-associated tumor suppressor promotes antitumor immunity via macrophage polarization
Source: Nat Commun. 2021 Jul 14;12:4300. doi: 10.1038/s41467-021-24610-x (PMC8280123; doi:10.1038/s41467-021-24610-x)

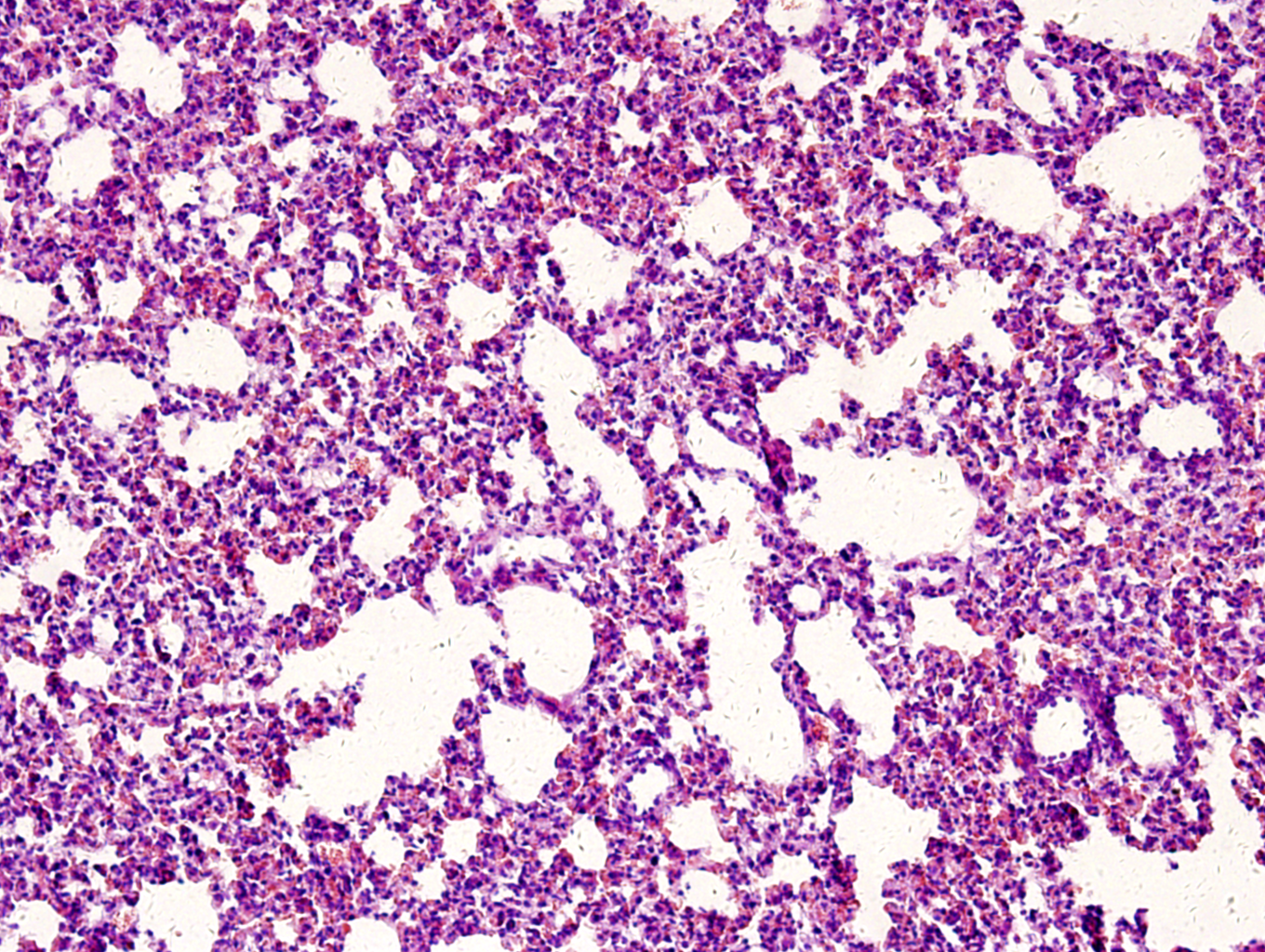

Supplement: Supplementary file 3 — Source Data [file 41467_2021_24610_MOESM3_ESM.zip › sounce data/Figure 1g HE/FKO 10x.tif]

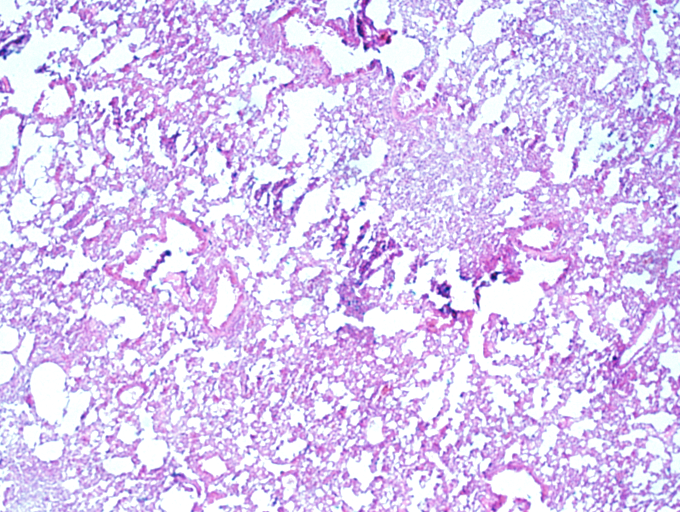

Supplement: Supplementary file 3 — Source Data [file 41467_2021_24610_MOESM3_ESM.zip › sounce data/Figure 1g HE/FKO1 4x.tif]

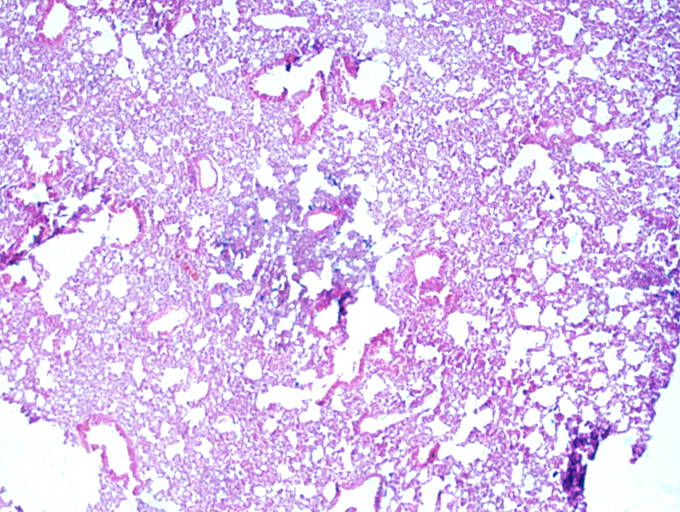

Supplement: Supplementary file 3 — Source Data [file 41467_2021_24610_MOESM3_ESM.zip › sounce data/Figure 1g HE/FKO2 4x.tif]

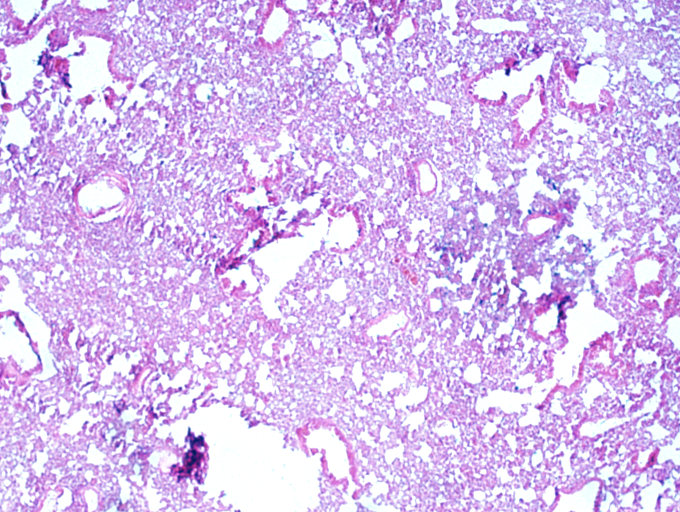

Supplement: Supplementary file 3 — Source Data [file 41467_2021_24610_MOESM3_ESM.zip › sounce data/Figure 1g HE/FKO3 4x.tif]

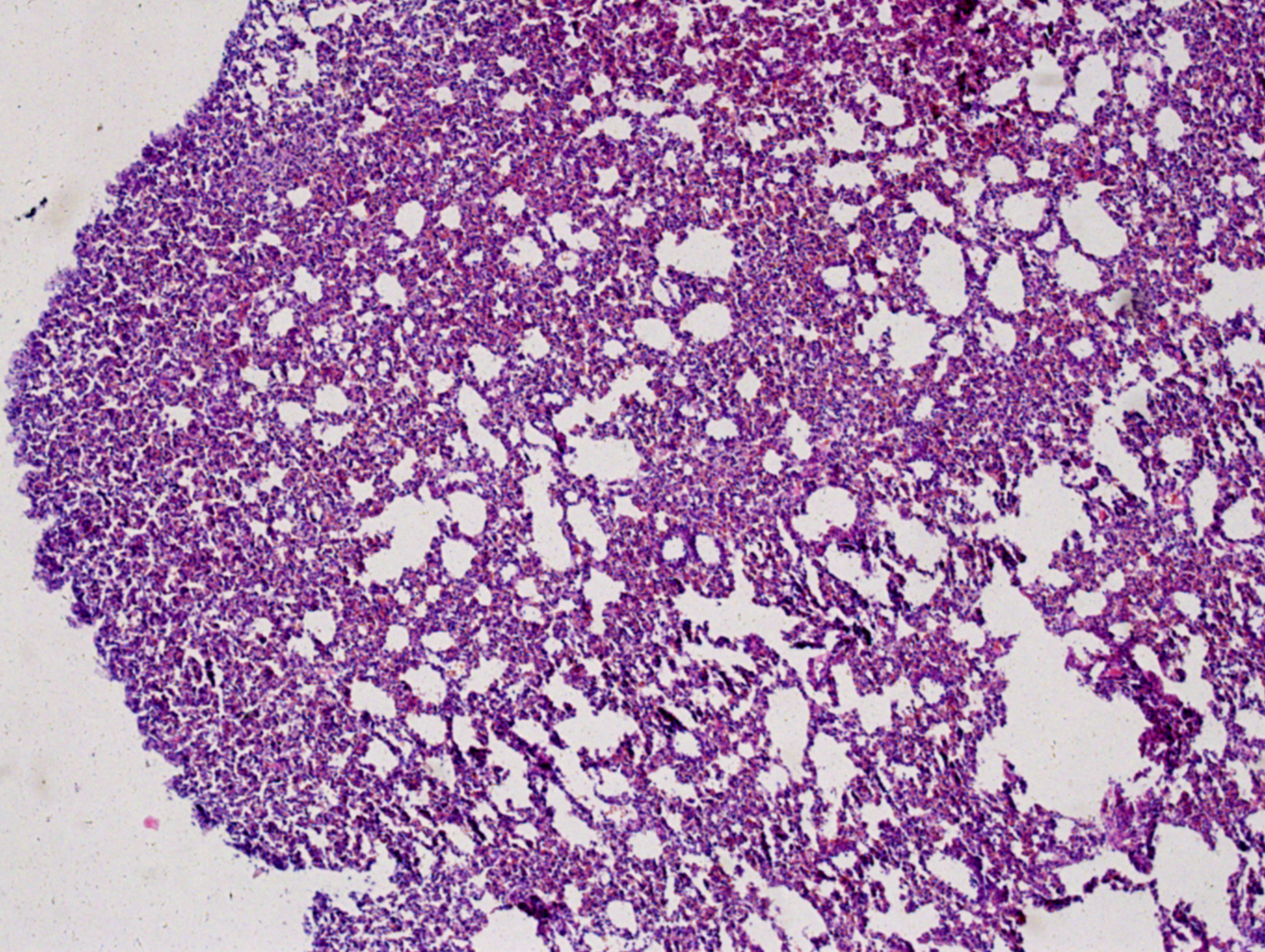

Supplement: Supplementary file 3 — Source Data [file 41467_2021_24610_MOESM3_ESM.zip › sounce data/Figure 1g HE/FKO4 4x.tif]

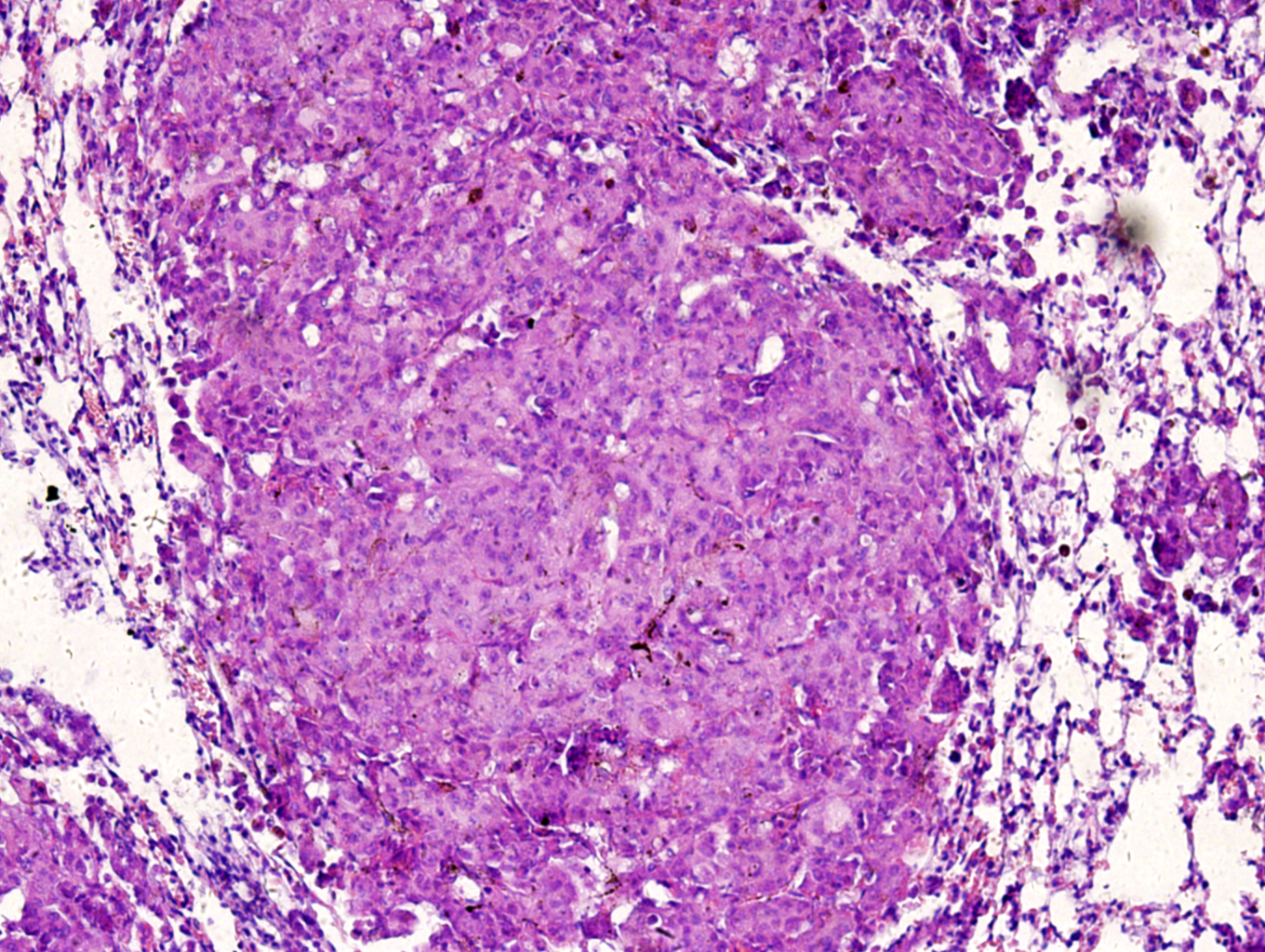

Supplement: Supplementary file 3 — Source Data [file 41467_2021_24610_MOESM3_ESM.zip › sounce data/Figure 1g HE/WT X10.tif]

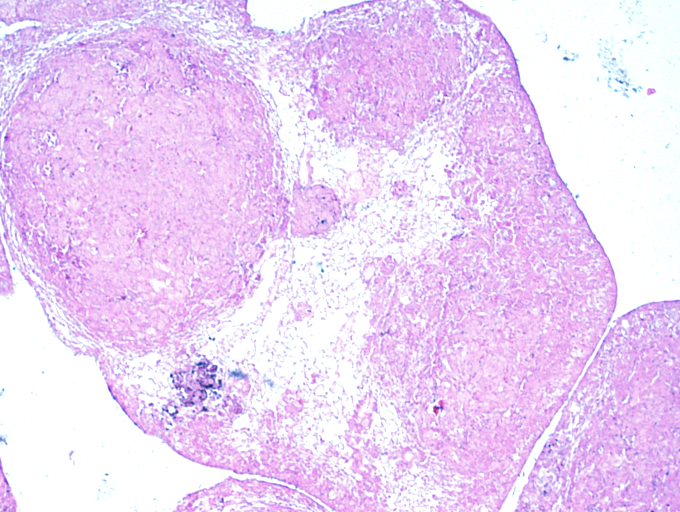

Supplement: Supplementary file 3 — Source Data [file 41467_2021_24610_MOESM3_ESM.zip › sounce data/Figure 1g HE/WT1 4x.tif]

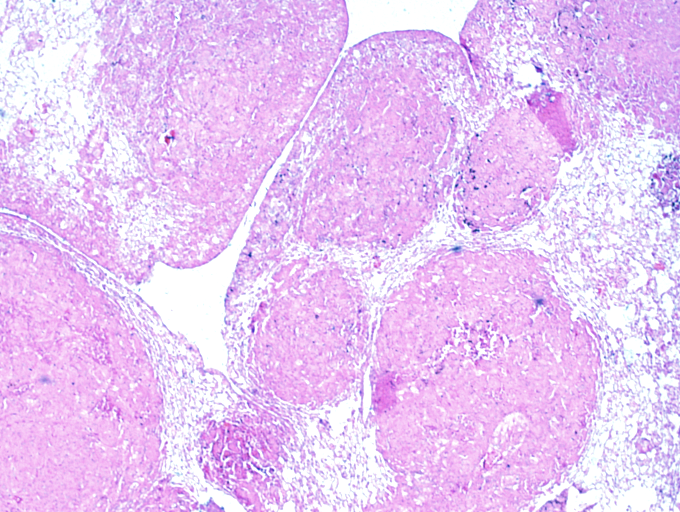

Supplement: Supplementary file 3 — Source Data [file 41467_2021_24610_MOESM3_ESM.zip › sounce data/Figure 1g HE/WT2 4x.tif]

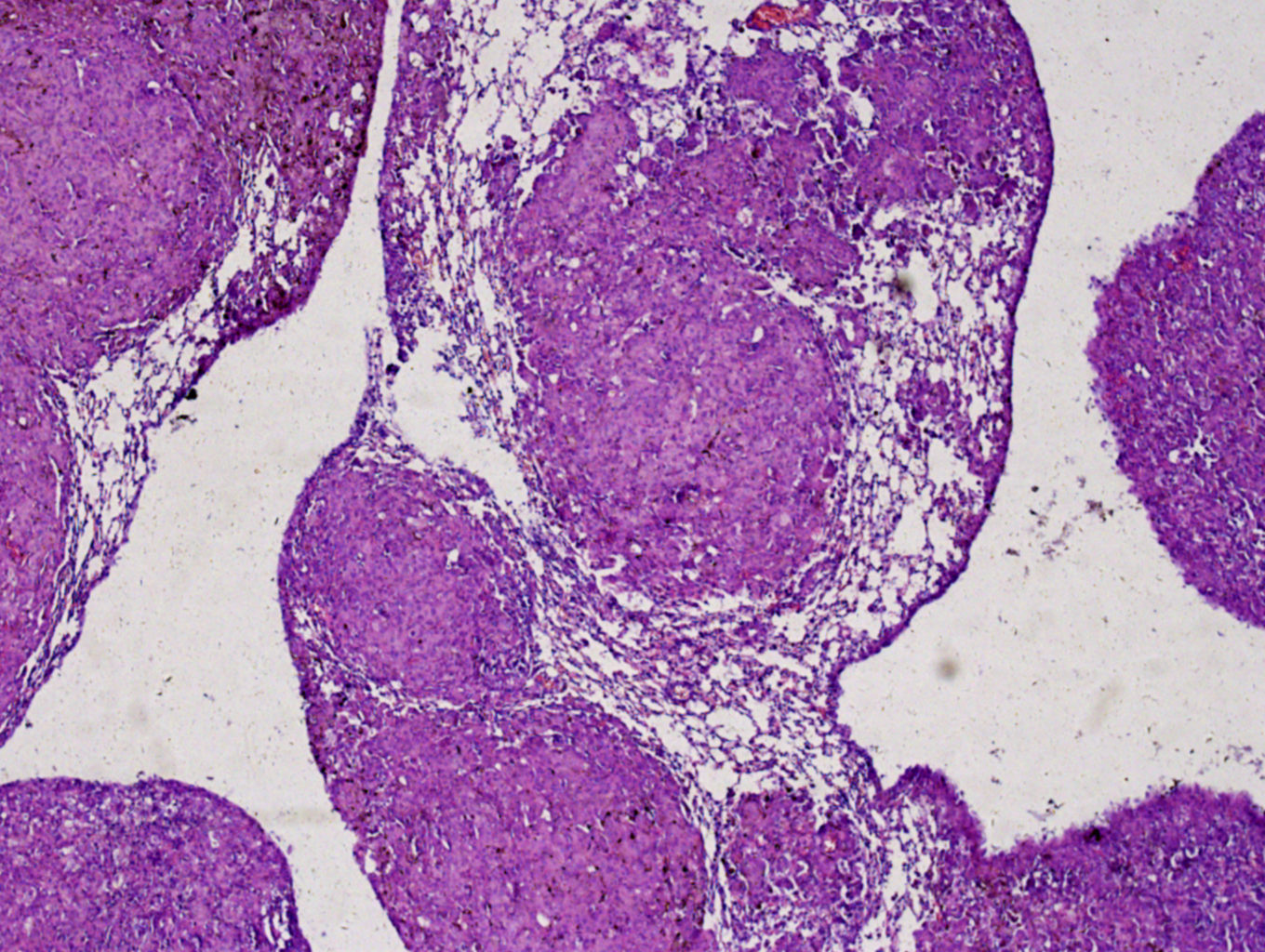

Supplement: Supplementary file 3 — Source Data [file 41467_2021_24610_MOESM3_ESM.zip › sounce data/Figure 1g HE/WT3 4X.tif]

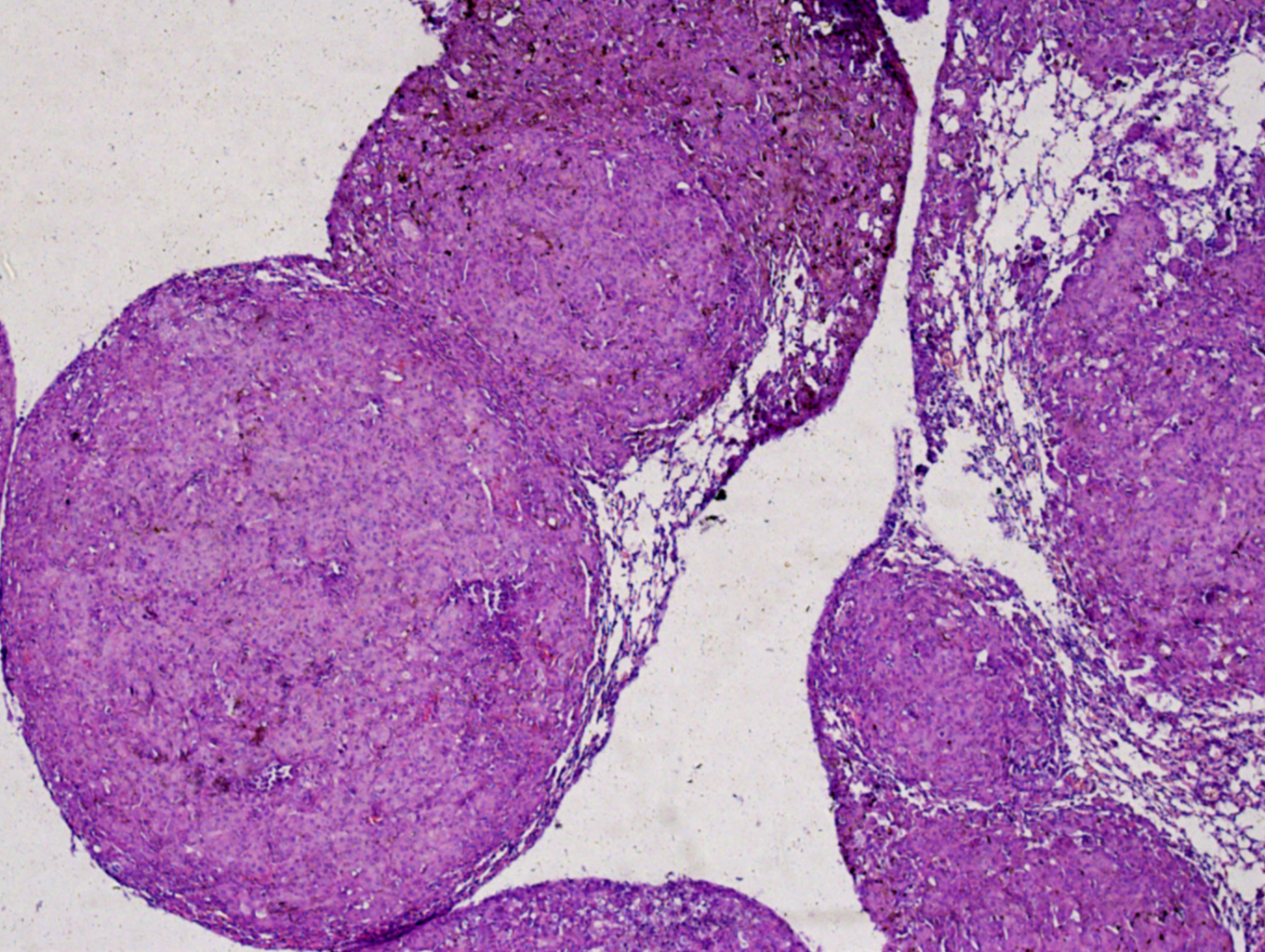

Supplement: Supplementary file 3 — Source Data [file 41467_2021_24610_MOESM3_ESM.zip › sounce data/Figure 1g HE/WT4 4X.tif]

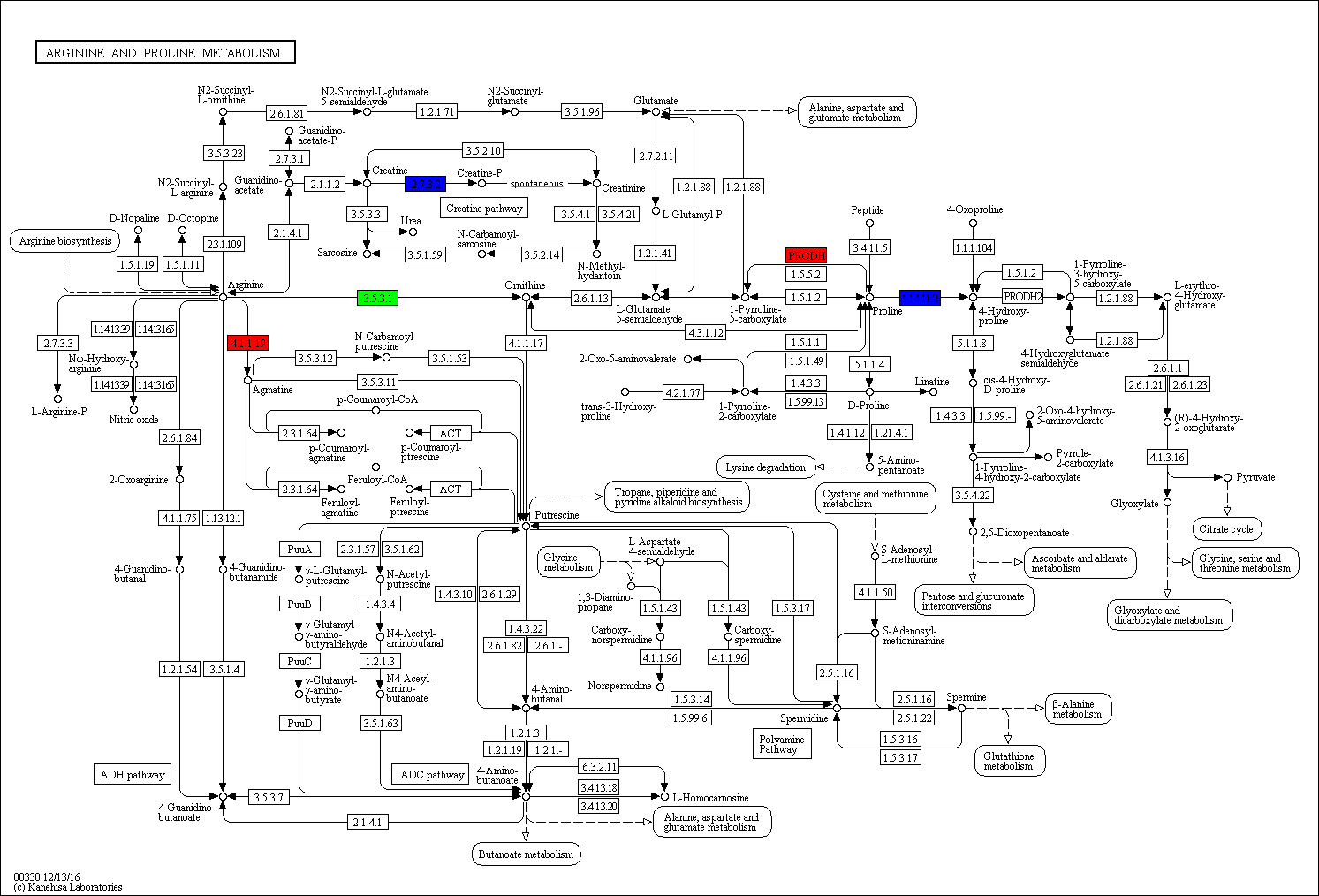

Supplement: Supplementary file 3 — Source Data [file 41467_2021_24610_MOESM3_ESM.zip › sounce data/RNAseq/path/CM0-VS-KOMO_ko00330.png]

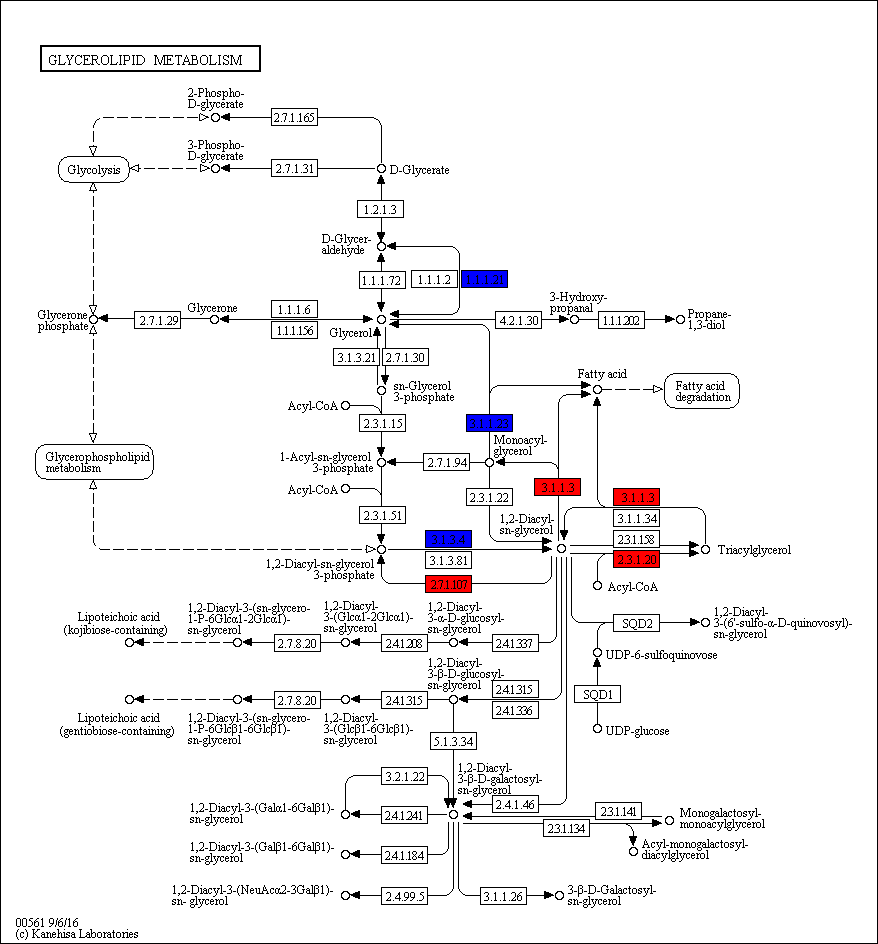

Supplement: Supplementary file 3 — Source Data [file 41467_2021_24610_MOESM3_ESM.zip › sounce data/RNAseq/path/CM0-VS-KOMO_ko00561.png]

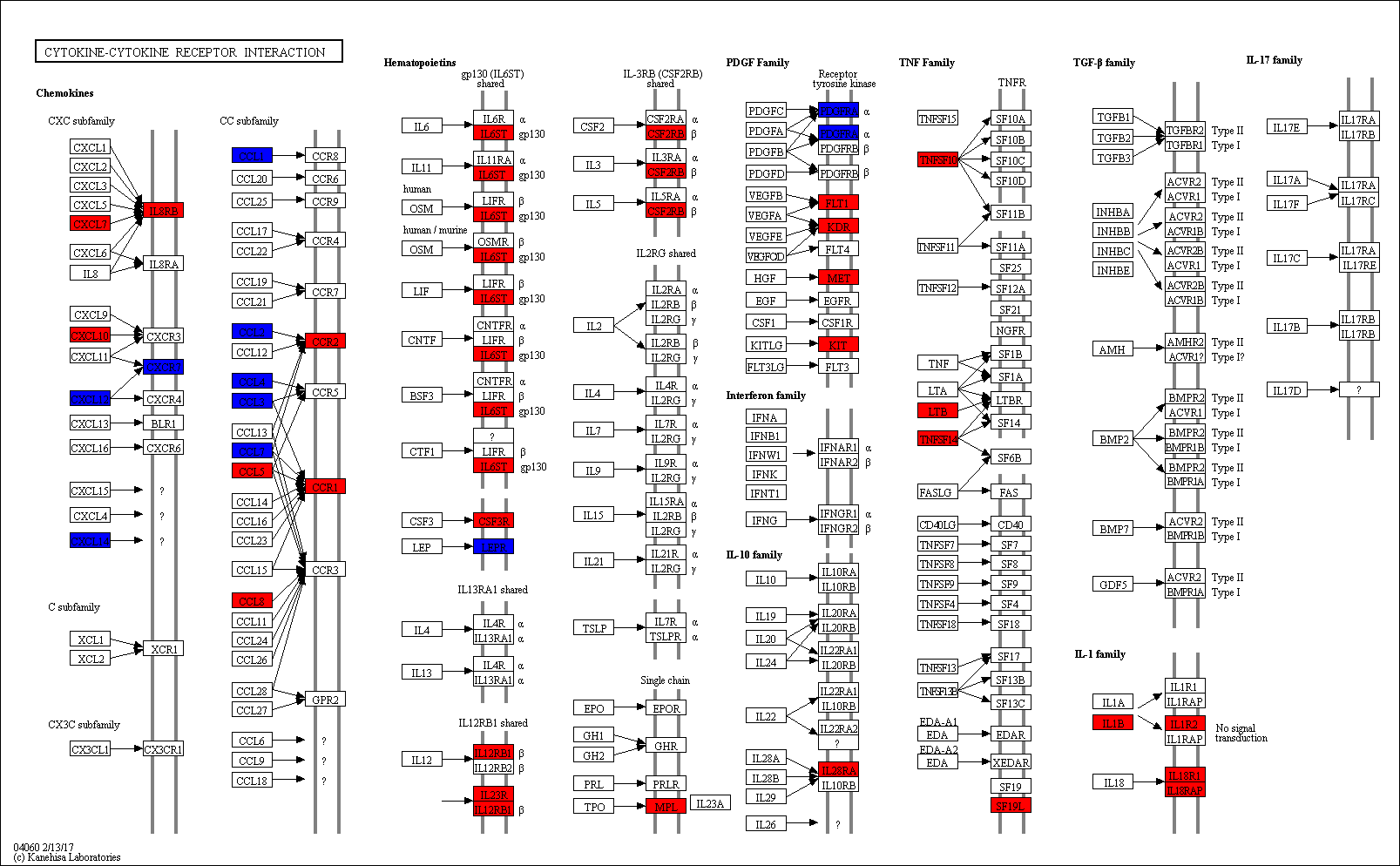

Supplement: Supplementary file 3 — Source Data [file 41467_2021_24610_MOESM3_ESM.zip › sounce data/RNAseq/path/CM0-VS-KOMO_ko04060.png]

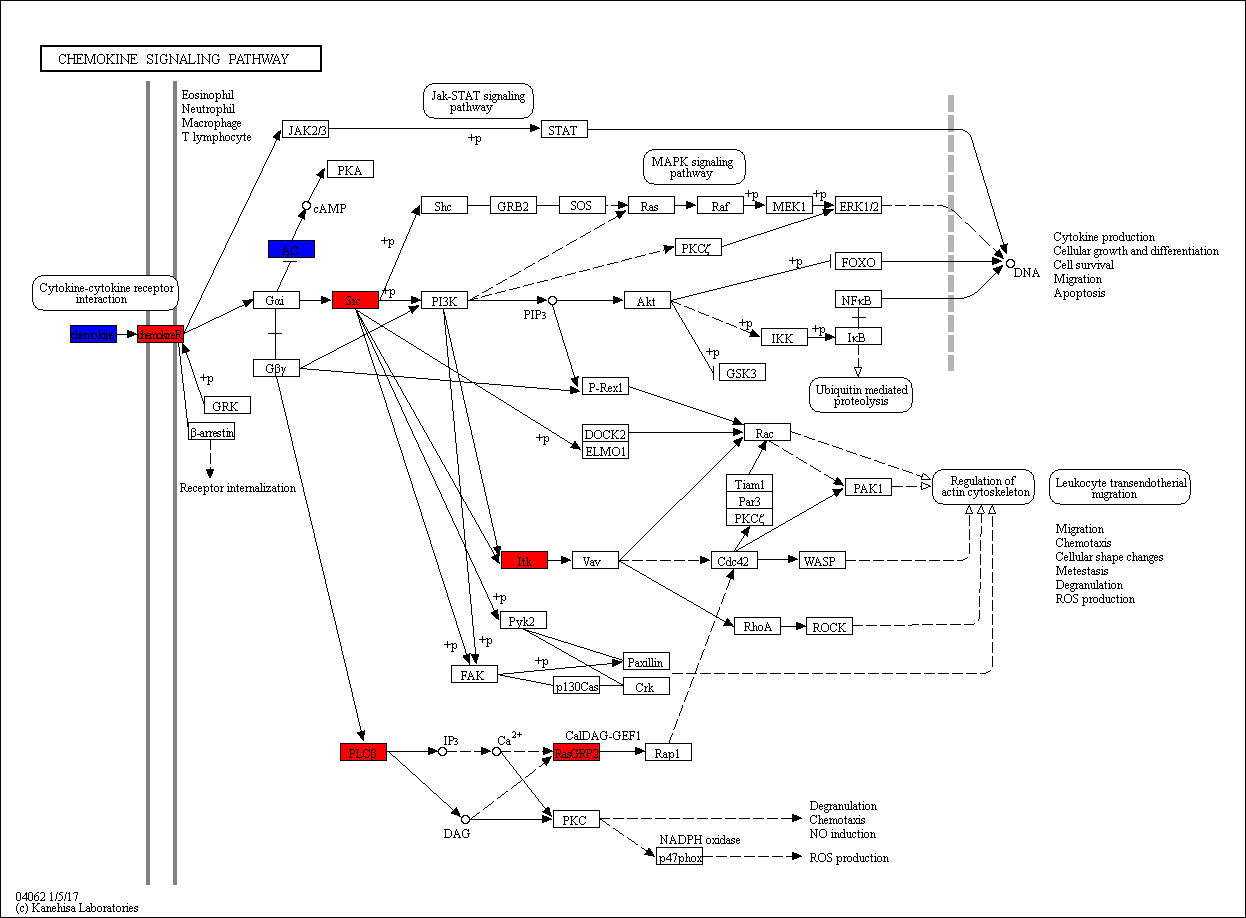

Supplement: Supplementary file 3 — Source Data [file 41467_2021_24610_MOESM3_ESM.zip › sounce data/RNAseq/path/CM0-VS-KOMO_ko04062.png]

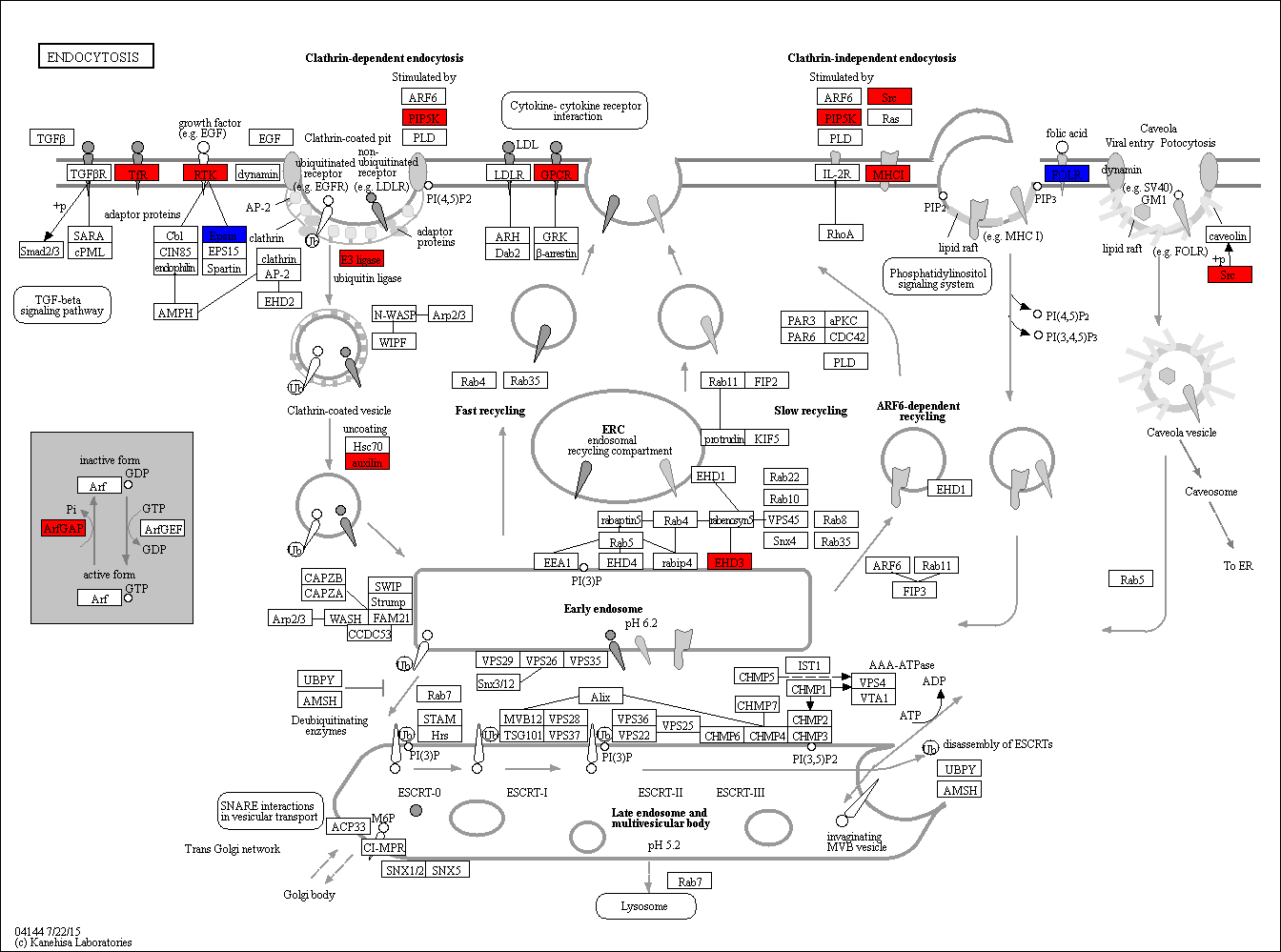

Supplement: Supplementary file 3 — Source Data [file 41467_2021_24610_MOESM3_ESM.zip › sounce data/RNAseq/path/CM0-VS-KOMO_ko04144.png]

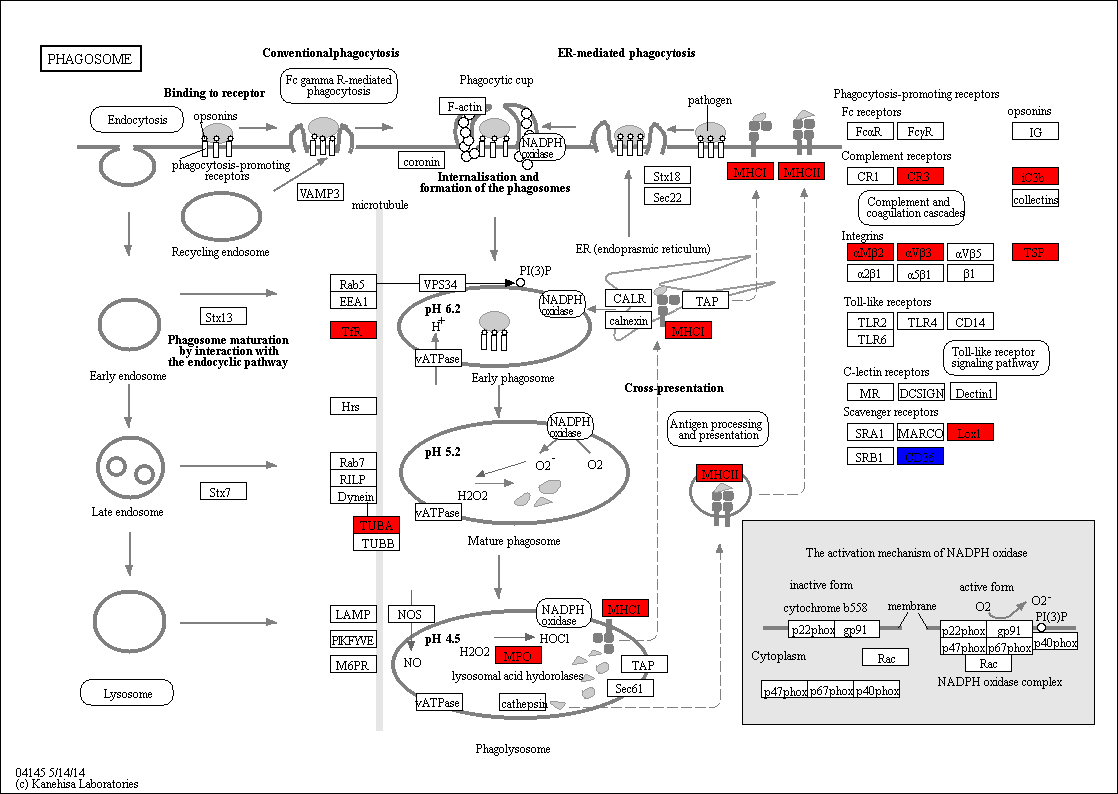

Supplement: Supplementary file 3 — Source Data [file 41467_2021_24610_MOESM3_ESM.zip › sounce data/RNAseq/path/CM0-VS-KOMO_ko04145.png]

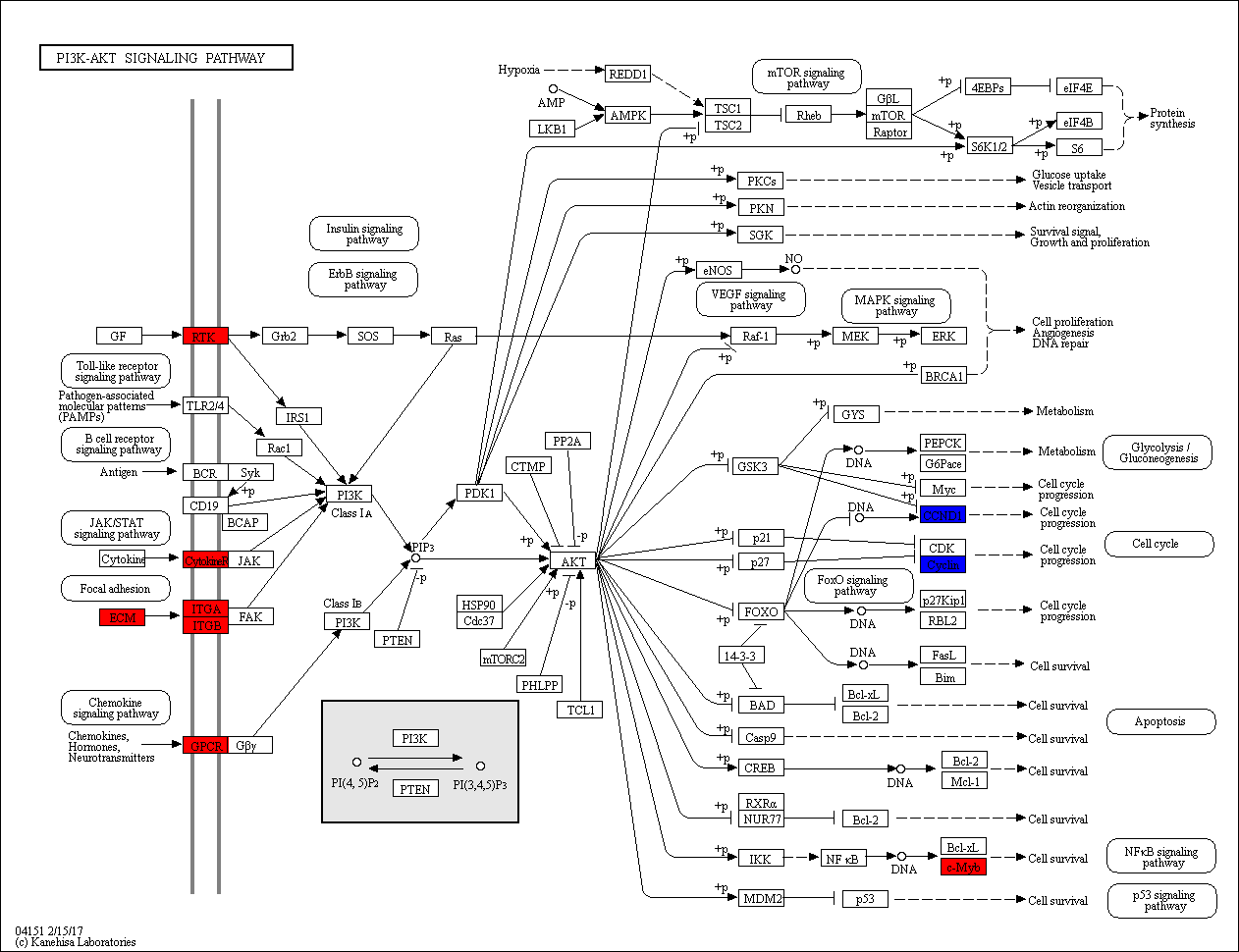

Supplement: Supplementary file 3 — Source Data [file 41467_2021_24610_MOESM3_ESM.zip › sounce data/RNAseq/path/CM0-VS-KOMO_ko04151.png]

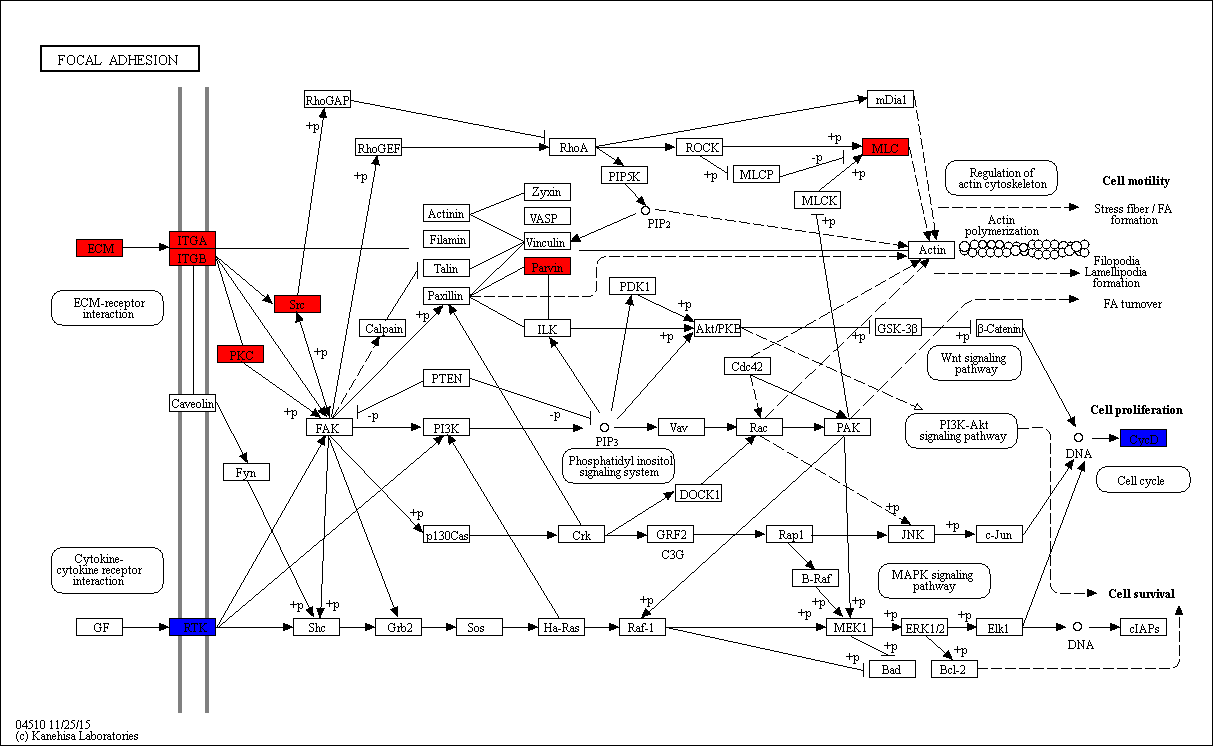

Supplement: Supplementary file 3 — Source Data [file 41467_2021_24610_MOESM3_ESM.zip › sounce data/RNAseq/path/CM0-VS-KOMO_ko04510.png]

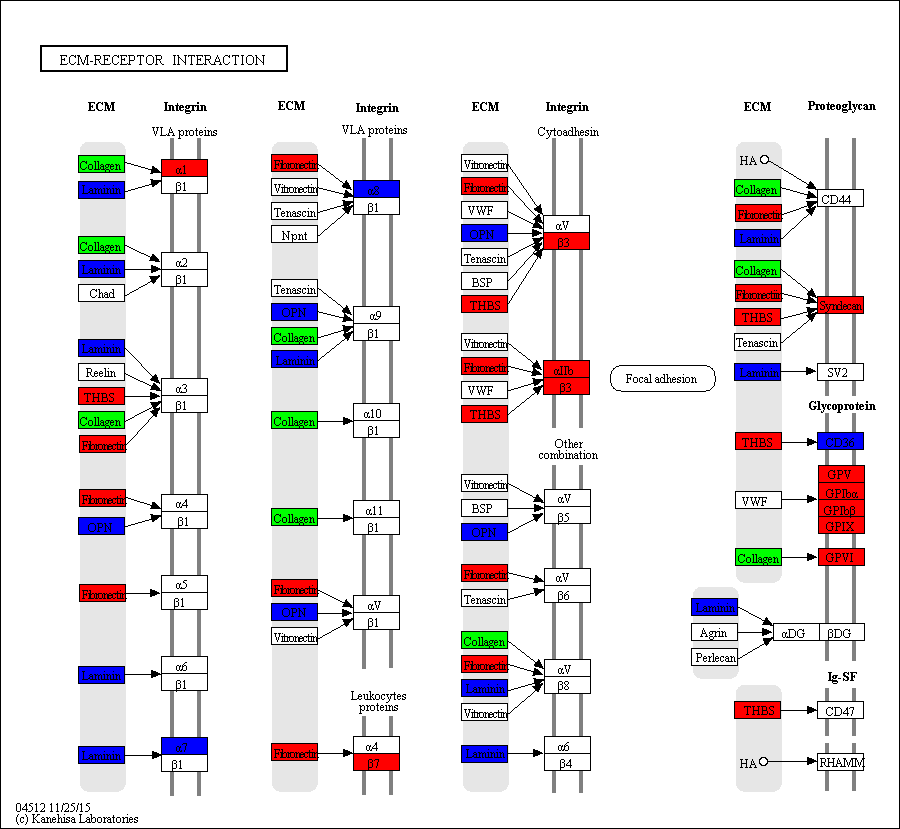

Supplement: Supplementary file 3 — Source Data [file 41467_2021_24610_MOESM3_ESM.zip › sounce data/RNAseq/path/CM0-VS-KOMO_ko04512.png]

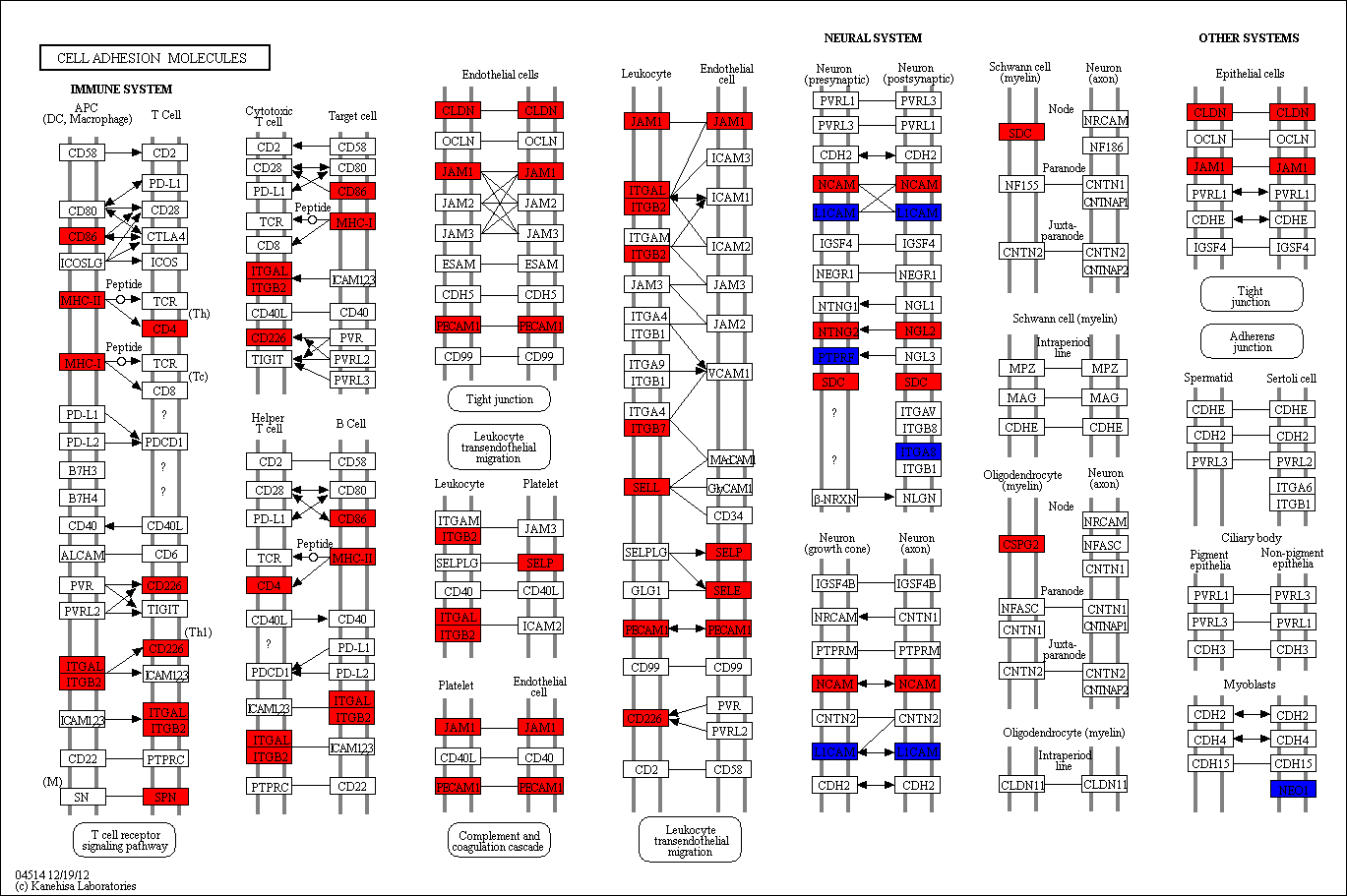

Supplement: Supplementary file 3 — Source Data [file 41467_2021_24610_MOESM3_ESM.zip › sounce data/RNAseq/path/CM0-VS-KOMO_ko04514.png]

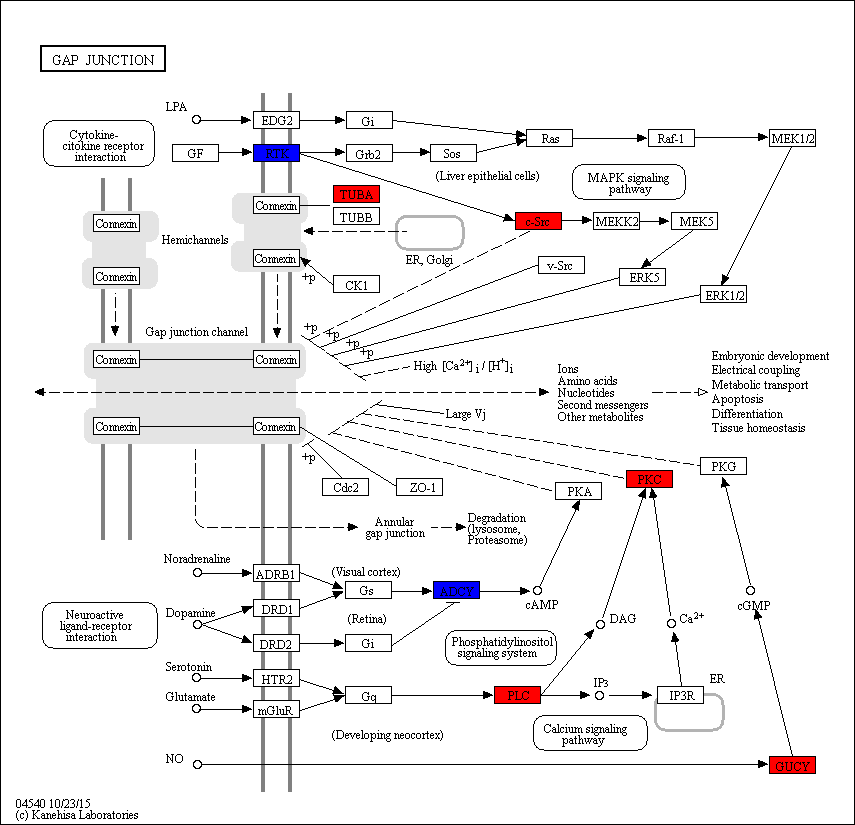

Supplement: Supplementary file 3 — Source Data [file 41467_2021_24610_MOESM3_ESM.zip › sounce data/RNAseq/path/CM0-VS-KOMO_ko04540.png]

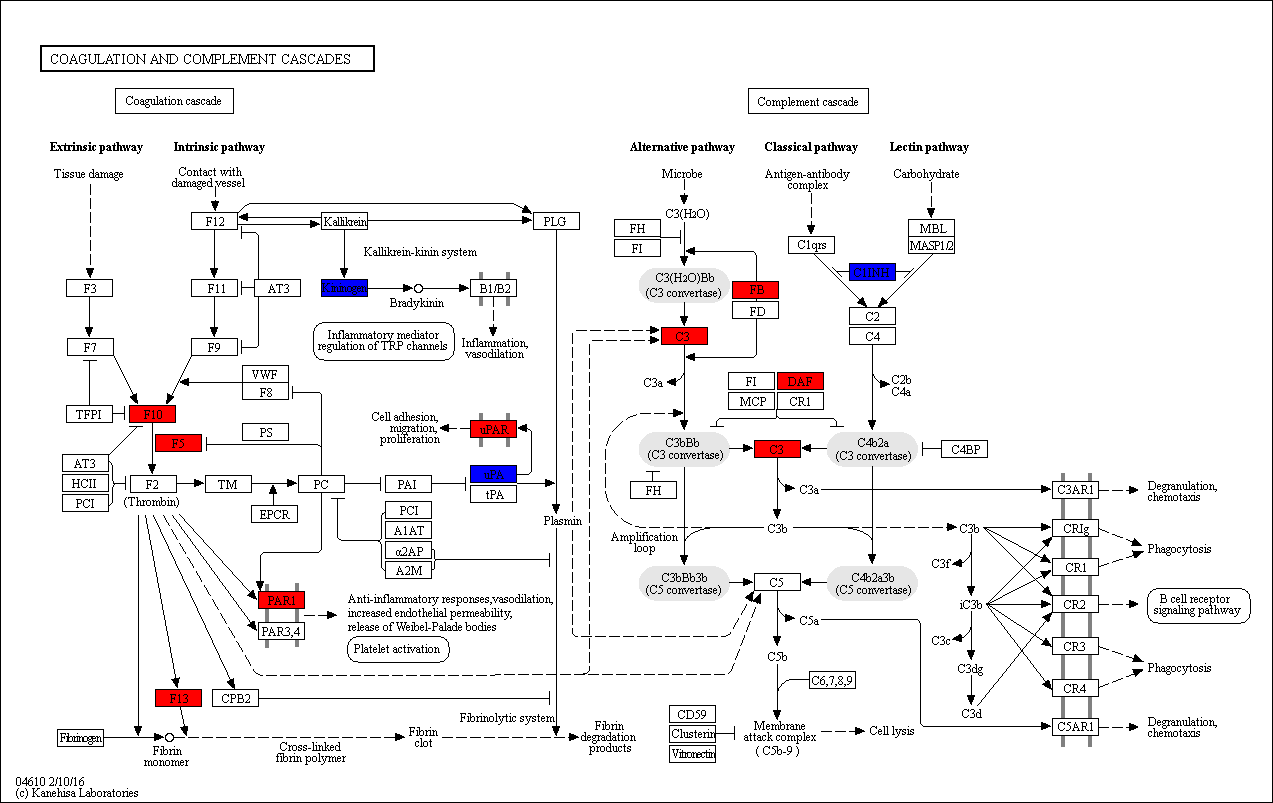

Supplement: Supplementary file 3 — Source Data [file 41467_2021_24610_MOESM3_ESM.zip › sounce data/RNAseq/path/CM0-VS-KOMO_ko04610.png]

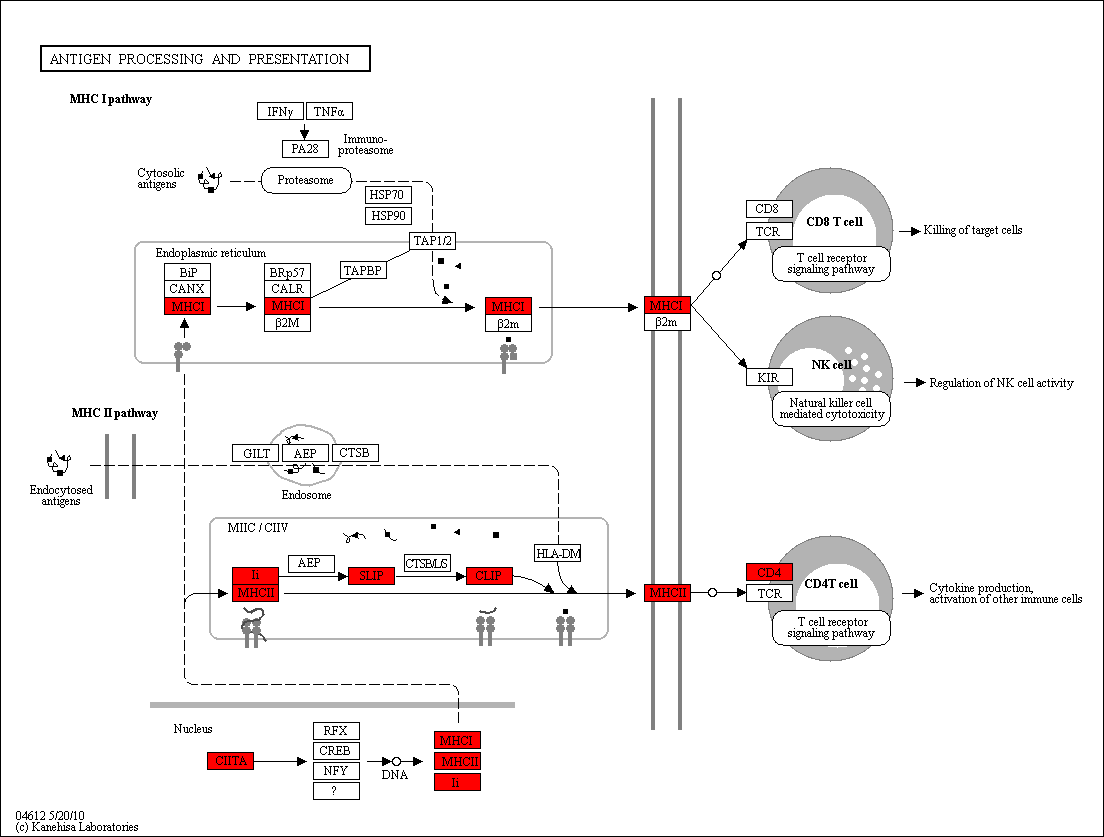

Supplement: Supplementary file 3 — Source Data [file 41467_2021_24610_MOESM3_ESM.zip › sounce data/RNAseq/path/CM0-VS-KOMO_ko04612.png]

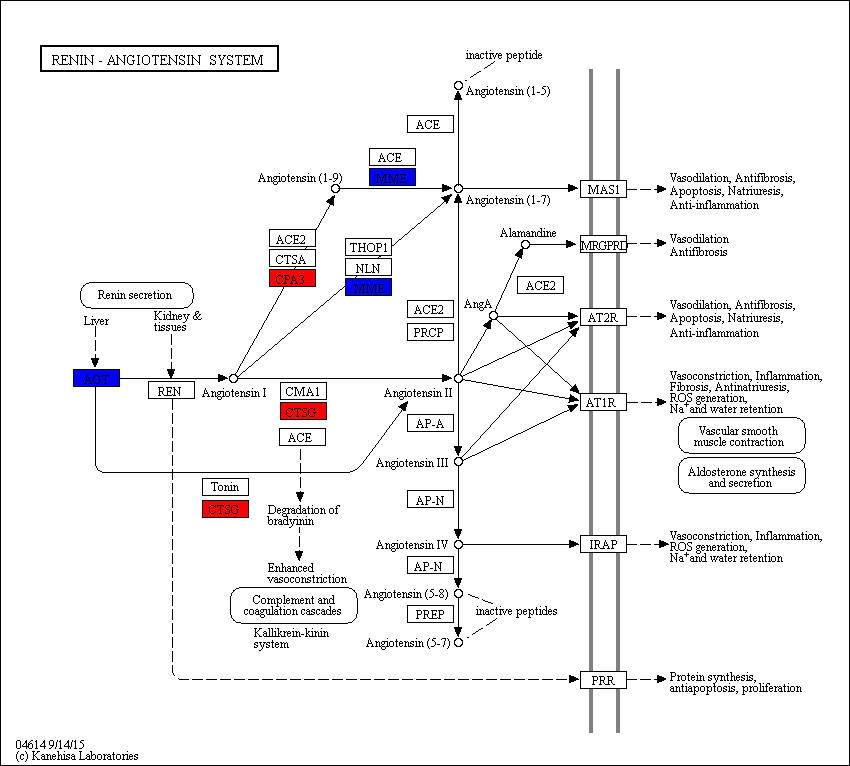

Supplement: Supplementary file 3 — Source Data [file 41467_2021_24610_MOESM3_ESM.zip › sounce data/RNAseq/path/CM0-VS-KOMO_ko04614.png]

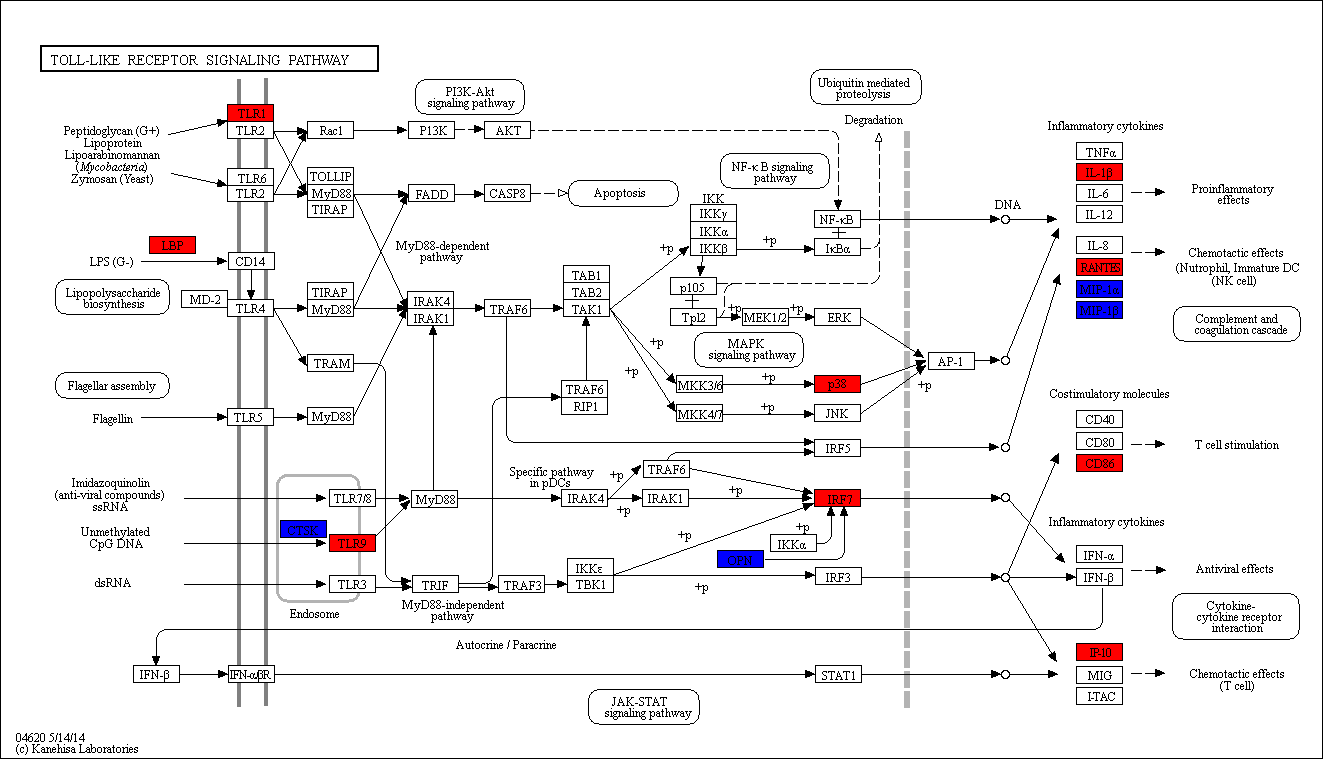

Supplement: Supplementary file 3 — Source Data [file 41467_2021_24610_MOESM3_ESM.zip › sounce data/RNAseq/path/CM0-VS-KOMO_ko04620.png]

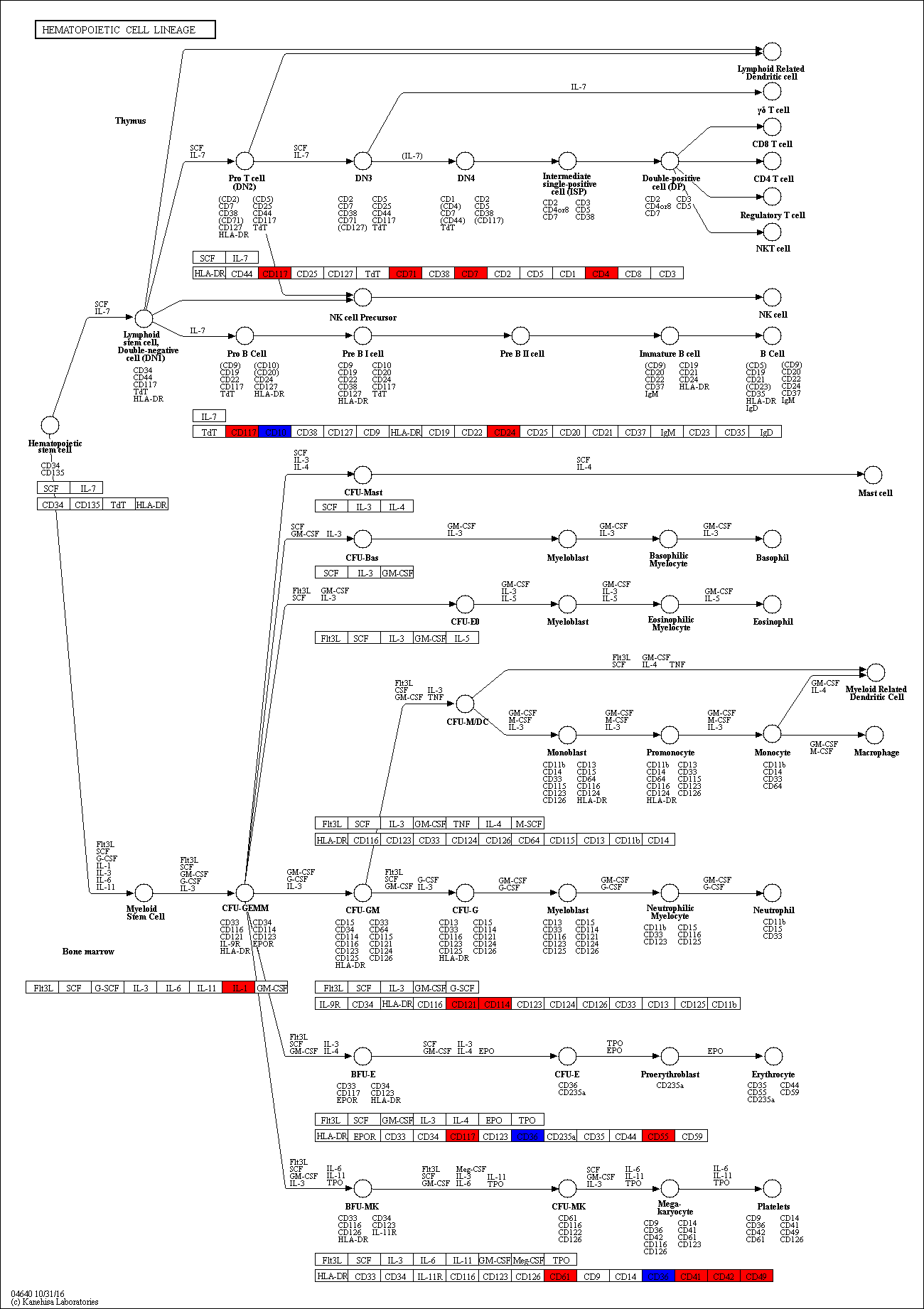

Supplement: Supplementary file 3 — Source Data [file 41467_2021_24610_MOESM3_ESM.zip › sounce data/RNAseq/path/CM0-VS-KOMO_ko04640.png]

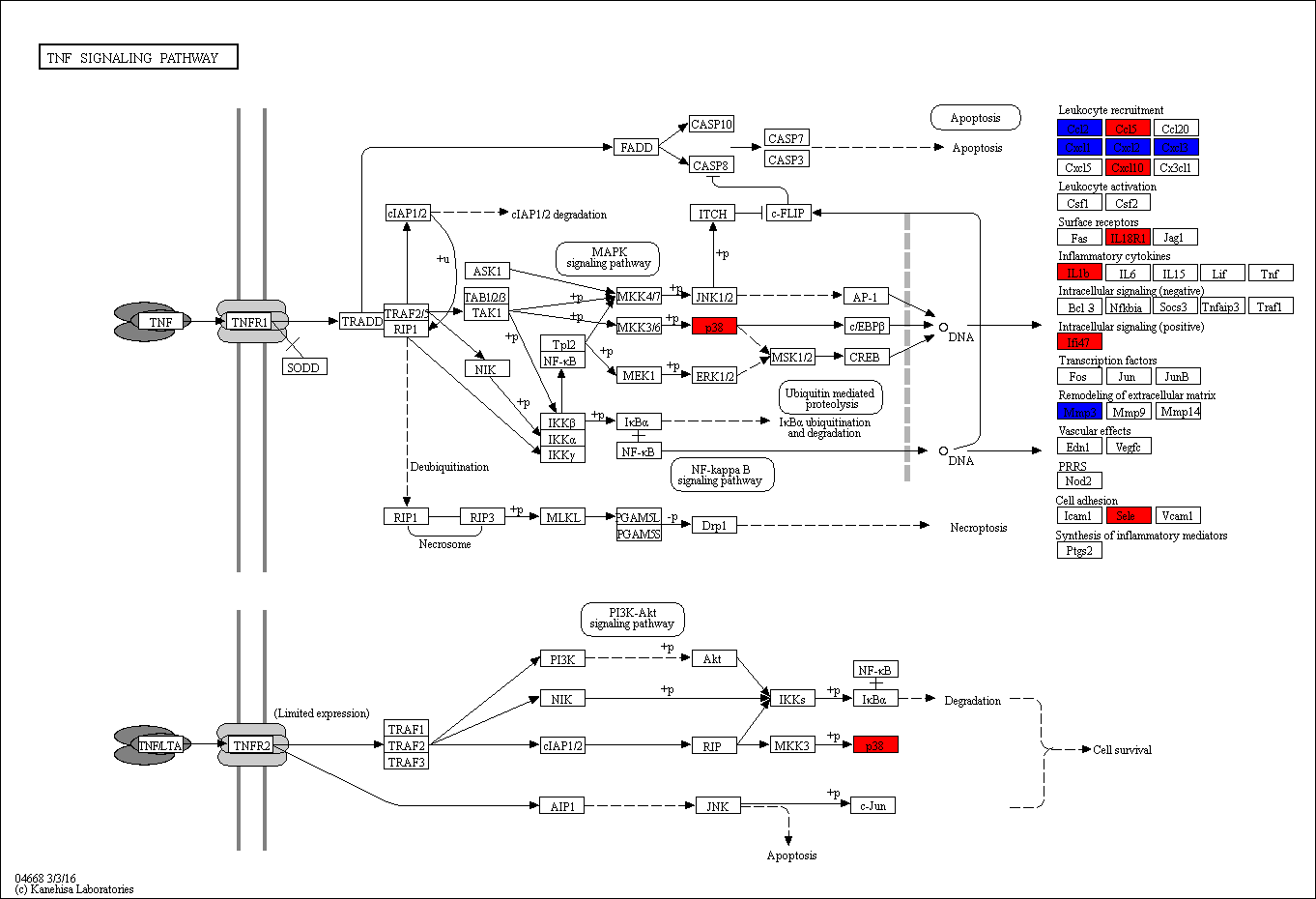

Supplement: Supplementary file 3 — Source Data [file 41467_2021_24610_MOESM3_ESM.zip › sounce data/RNAseq/path/CM0-VS-KOMO_ko04668.png]

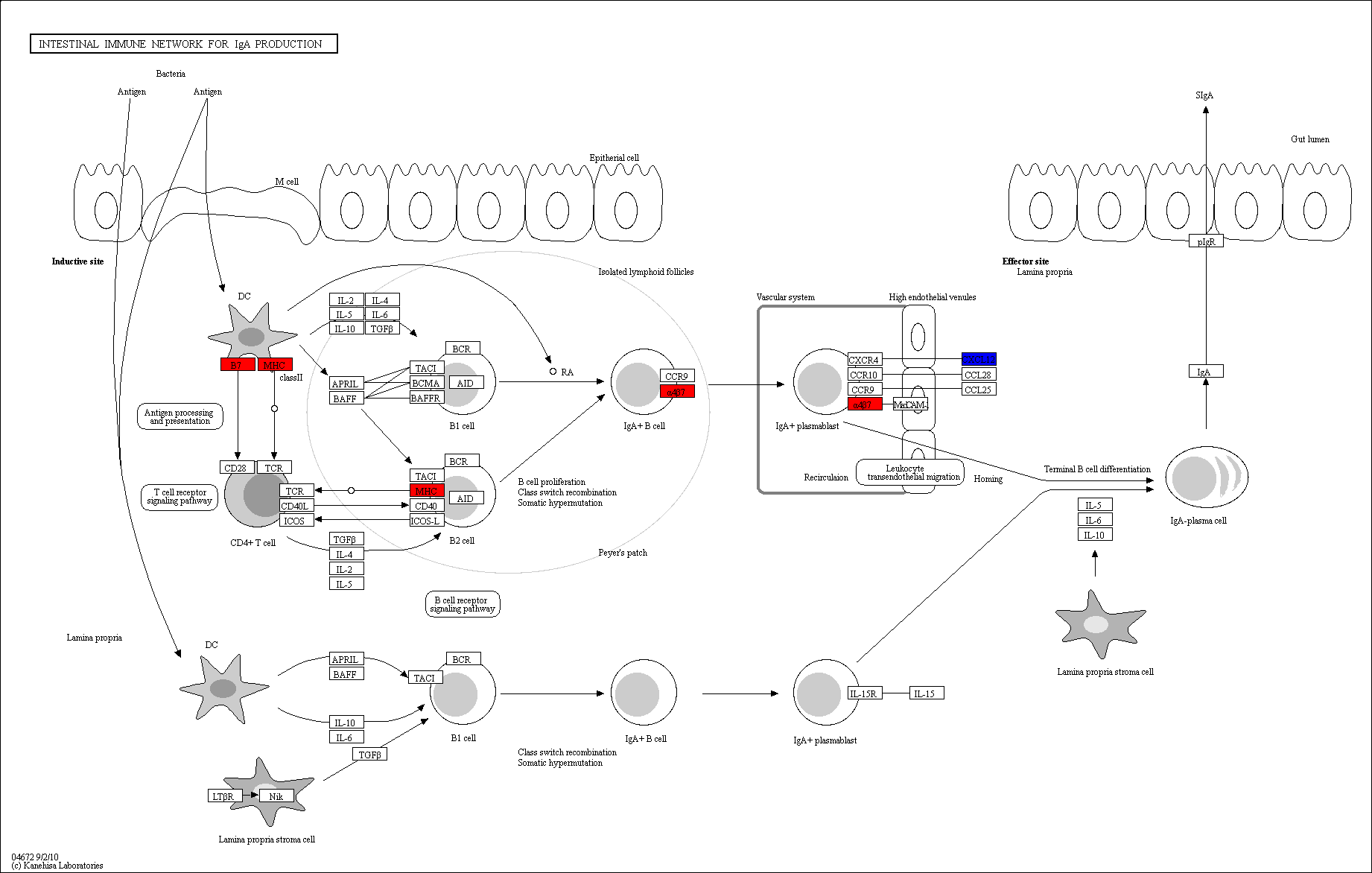

Supplement: Supplementary file 3 — Source Data [file 41467_2021_24610_MOESM3_ESM.zip › sounce data/RNAseq/path/CM0-VS-KOMO_ko04672.png]

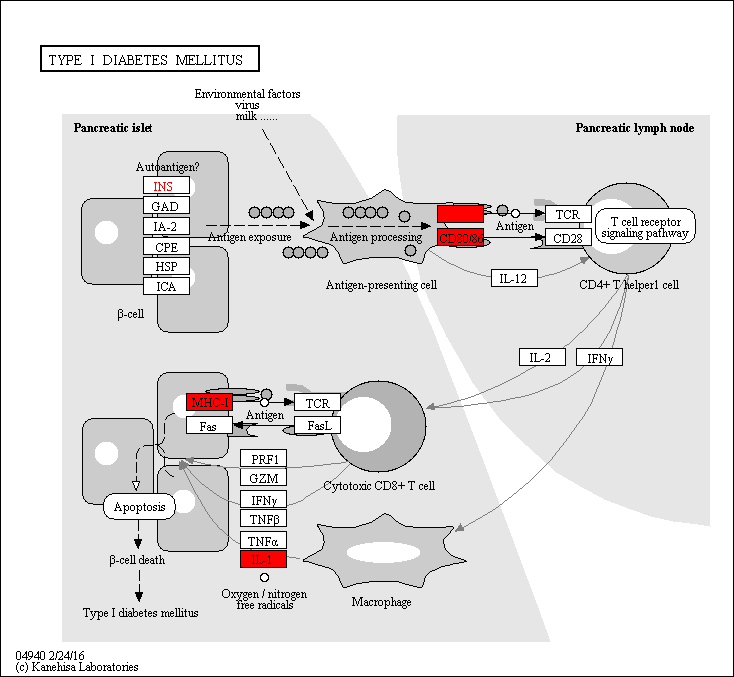

Supplement: Supplementary file 3 — Source Data [file 41467_2021_24610_MOESM3_ESM.zip › sounce data/RNAseq/path/CM0-VS-KOMO_ko04940.png]

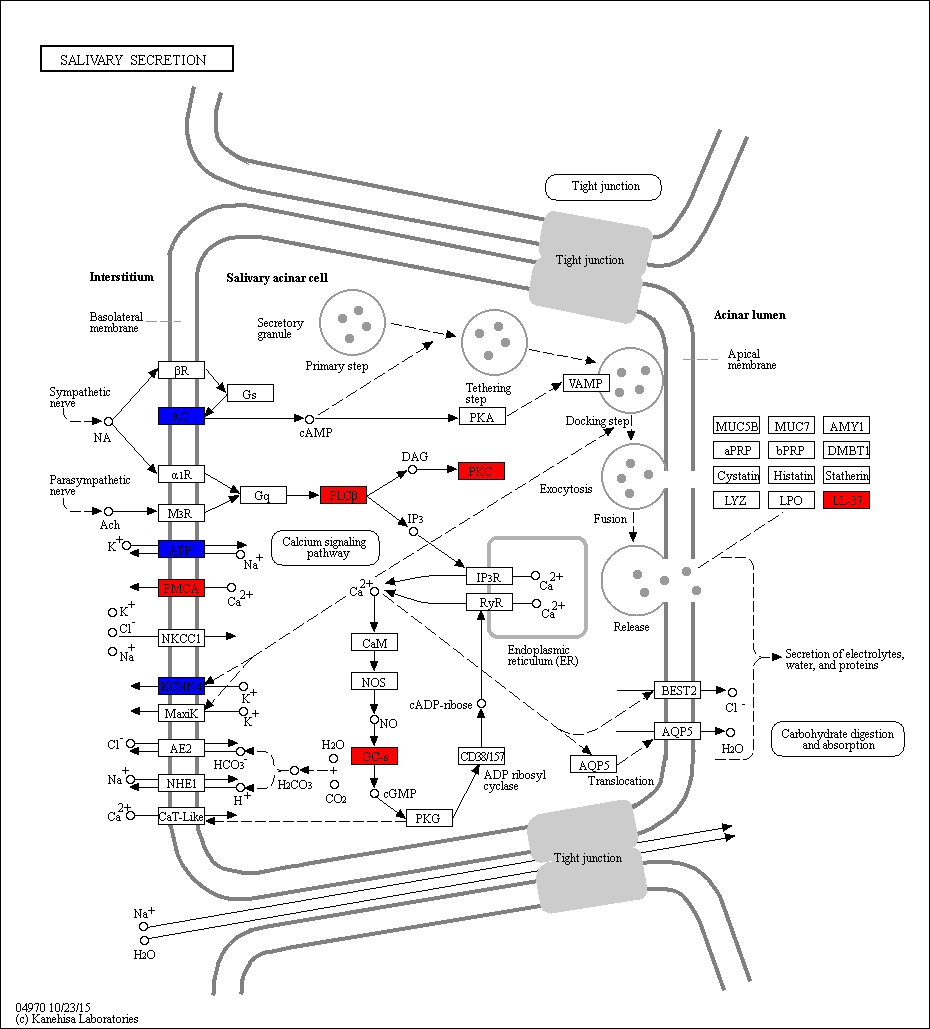

Supplement: Supplementary file 3 — Source Data [file 41467_2021_24610_MOESM3_ESM.zip › sounce data/RNAseq/path/CM0-VS-KOMO_ko04970.png]

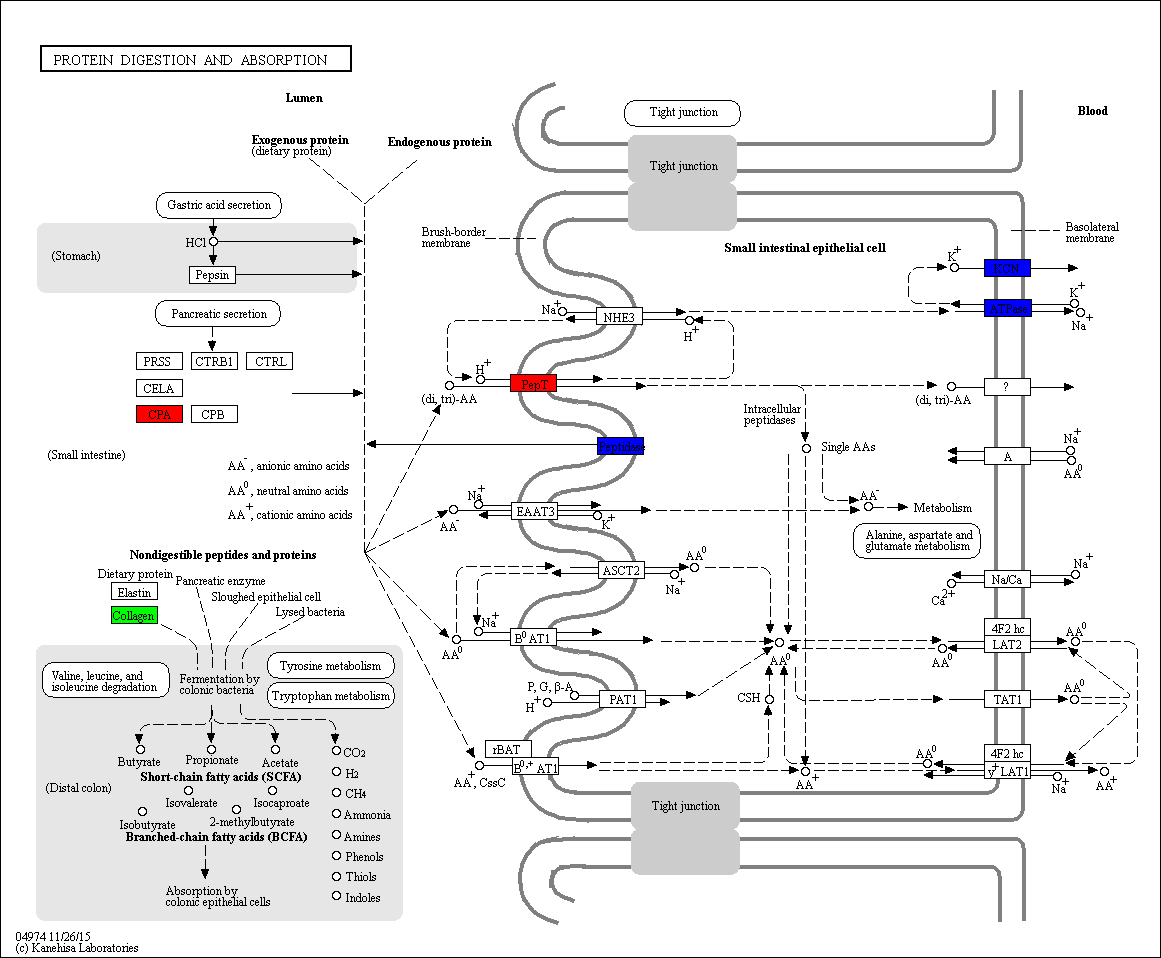

Supplement: Supplementary file 3 — Source Data [file 41467_2021_24610_MOESM3_ESM.zip › sounce data/RNAseq/path/CM0-VS-KOMO_ko04974.png]

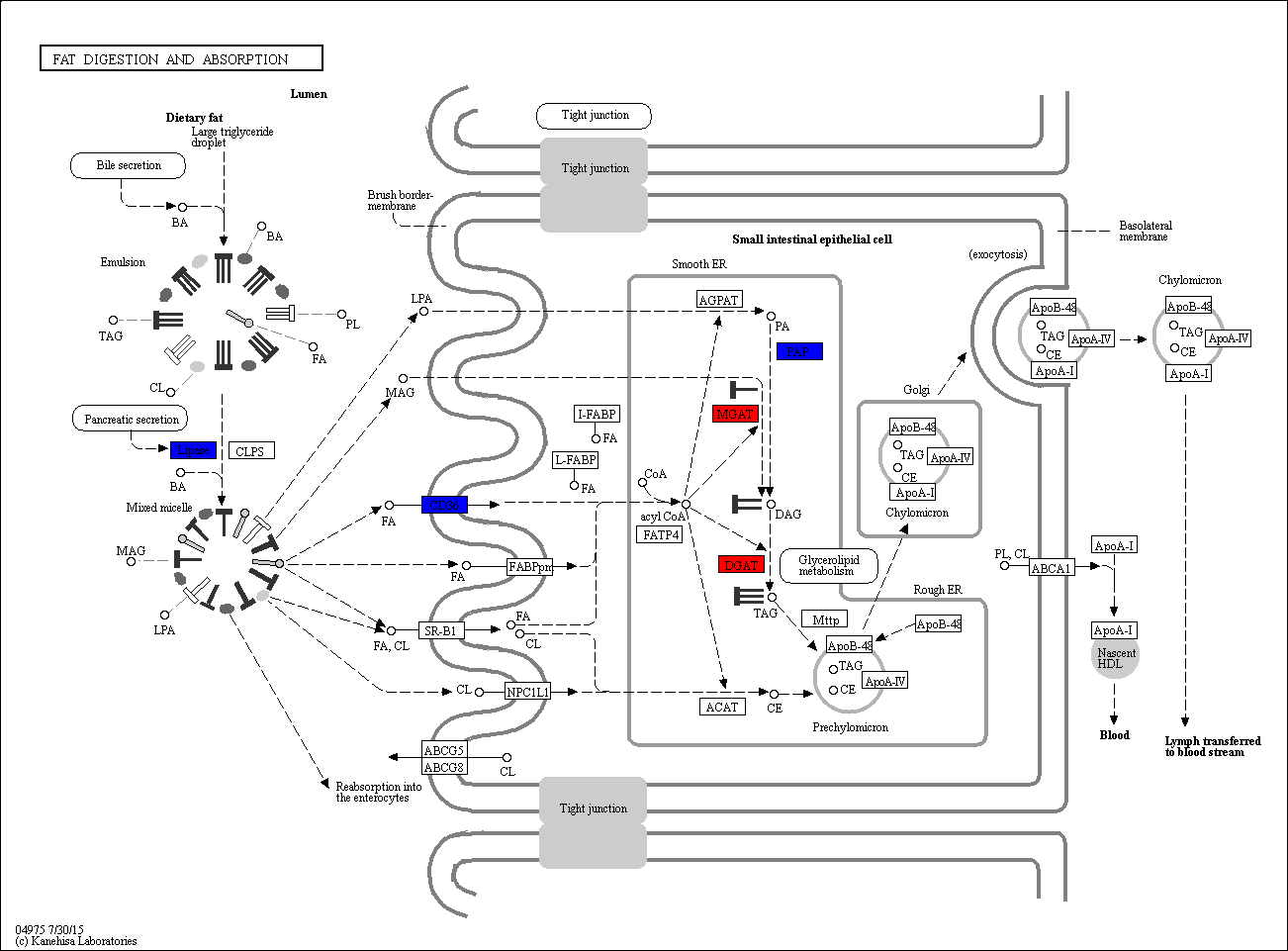

Supplement: Supplementary file 3 — Source Data [file 41467_2021_24610_MOESM3_ESM.zip › sounce data/RNAseq/path/CM0-VS-KOMO_ko04975.png]

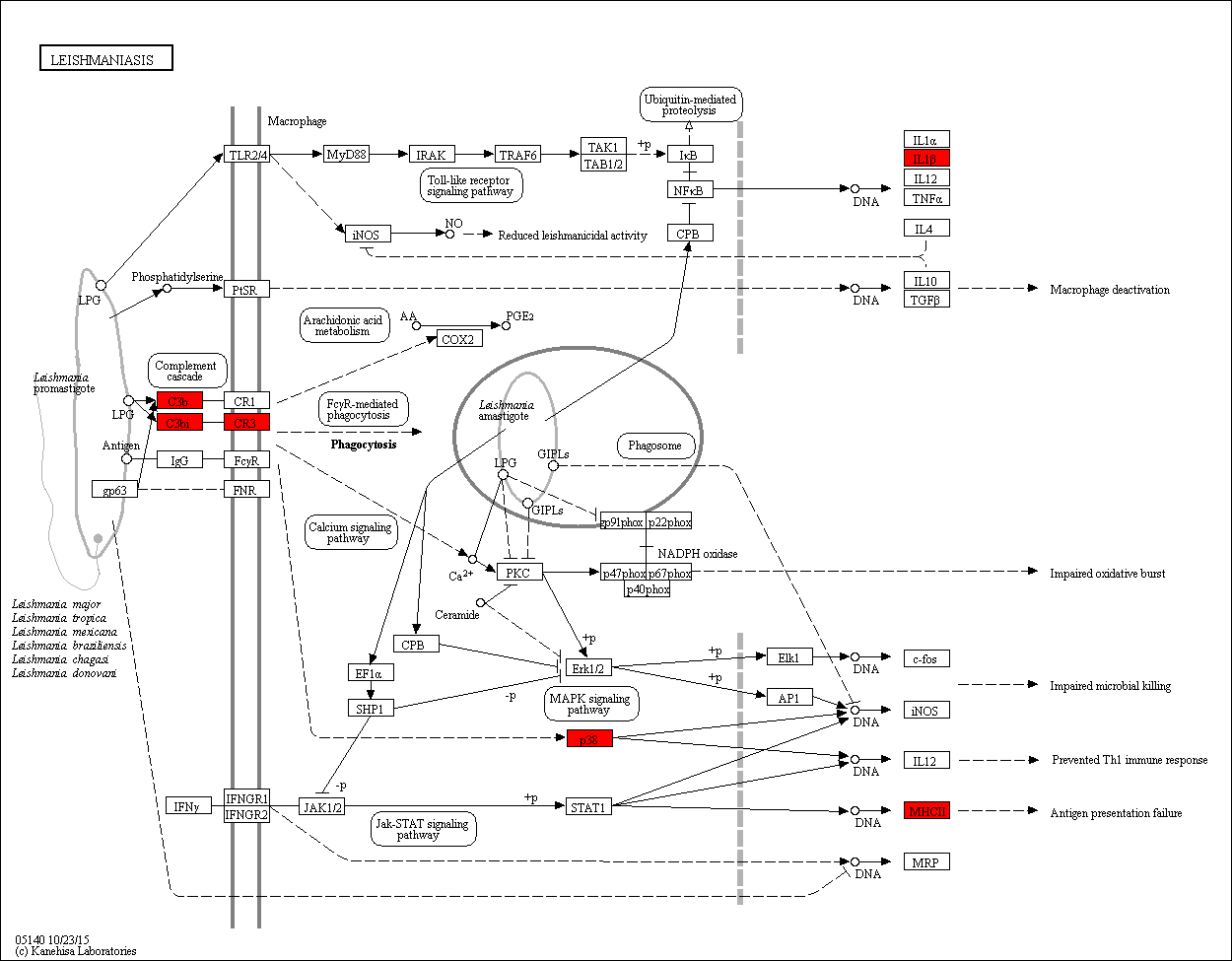

Supplement: Supplementary file 3 — Source Data [file 41467_2021_24610_MOESM3_ESM.zip › sounce data/RNAseq/path/CM0-VS-KOMO_ko05140.png]

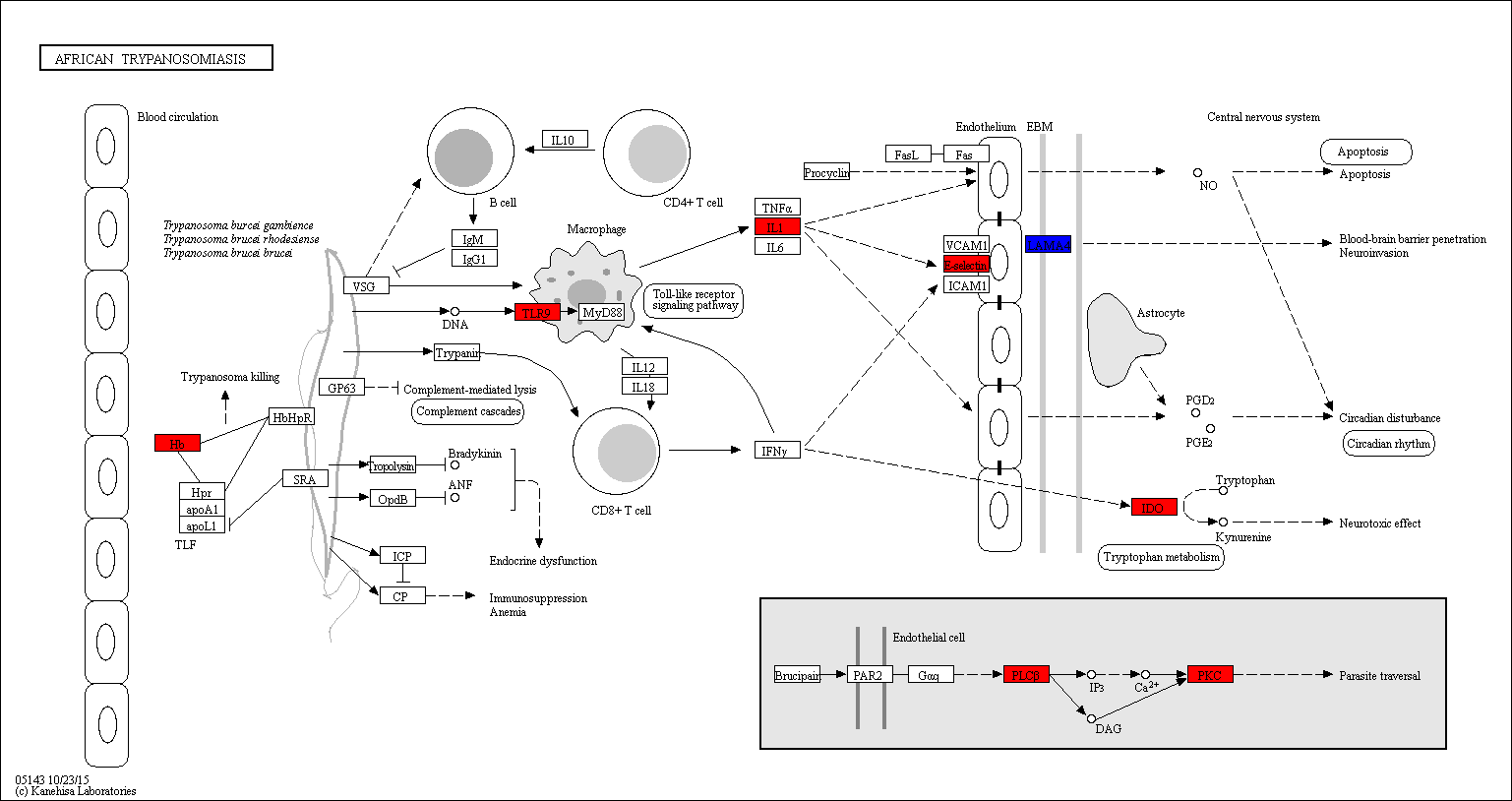

Supplement: Supplementary file 3 — Source Data [file 41467_2021_24610_MOESM3_ESM.zip › sounce data/RNAseq/path/CM0-VS-KOMO_ko05143.png]

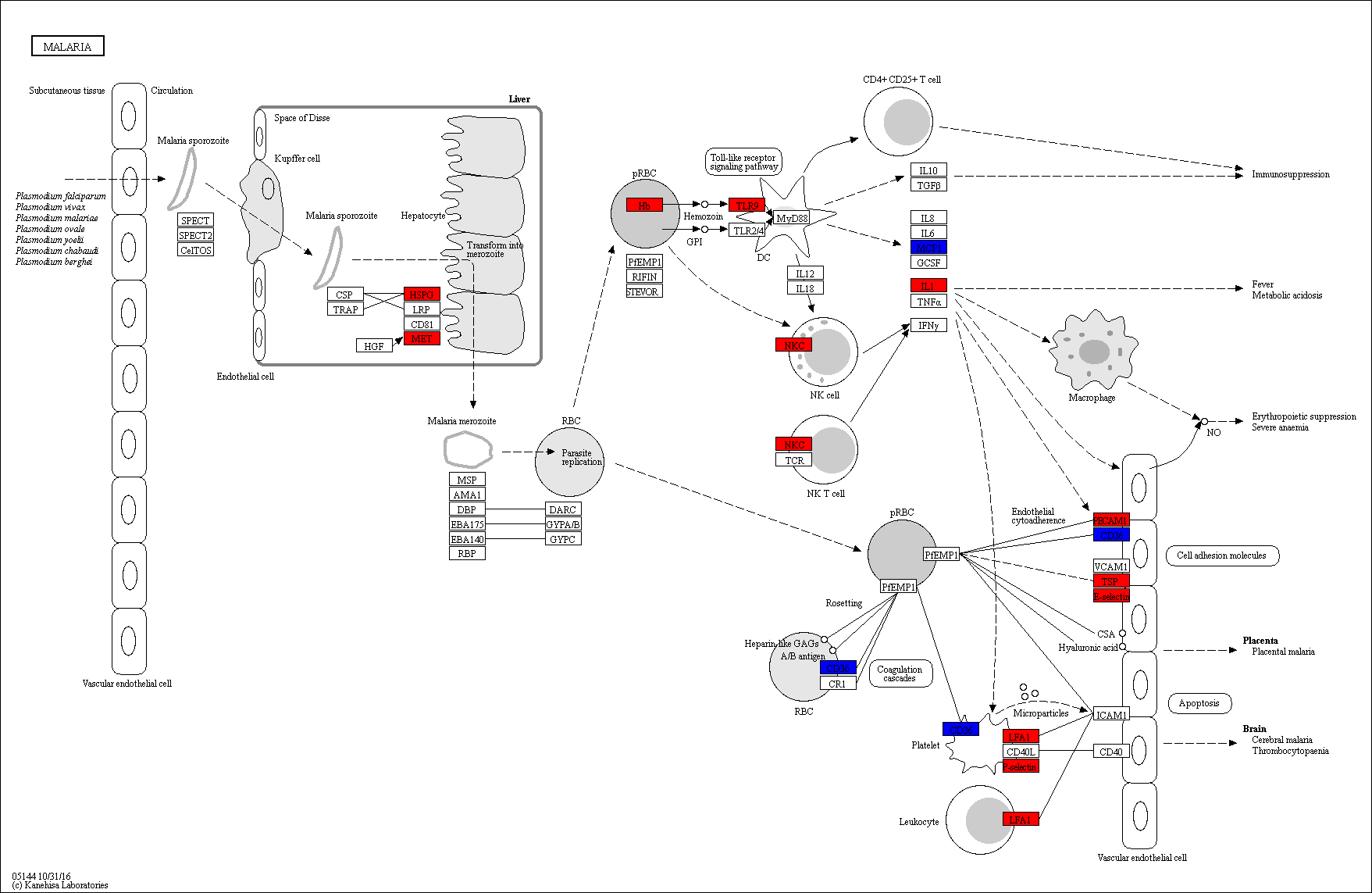

Supplement: Supplementary file 3 — Source Data [file 41467_2021_24610_MOESM3_ESM.zip › sounce data/RNAseq/path/CM0-VS-KOMO_ko05144.png]

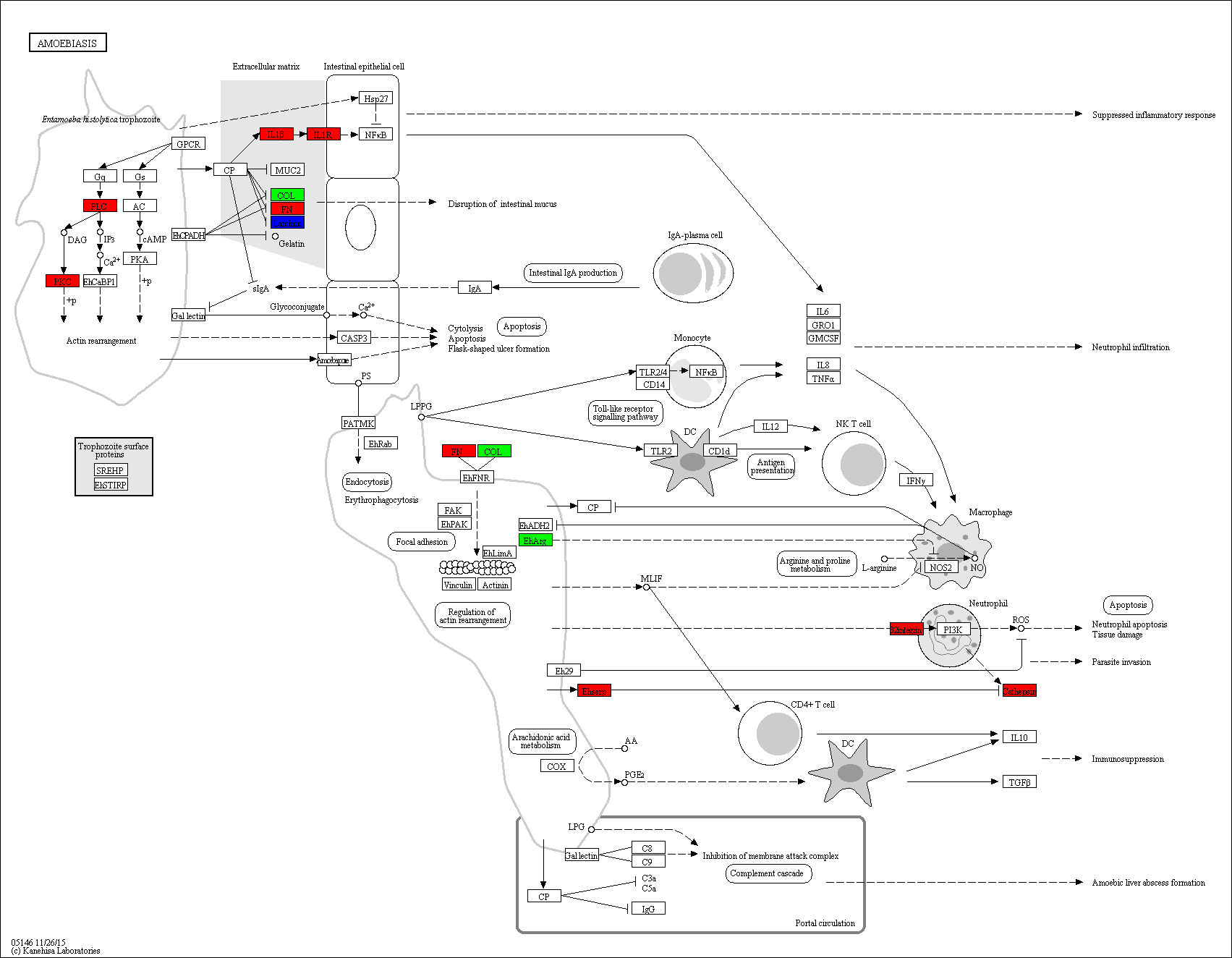

Supplement: Supplementary file 3 — Source Data [file 41467_2021_24610_MOESM3_ESM.zip › sounce data/RNAseq/path/CM0-VS-KOMO_ko05146.png]

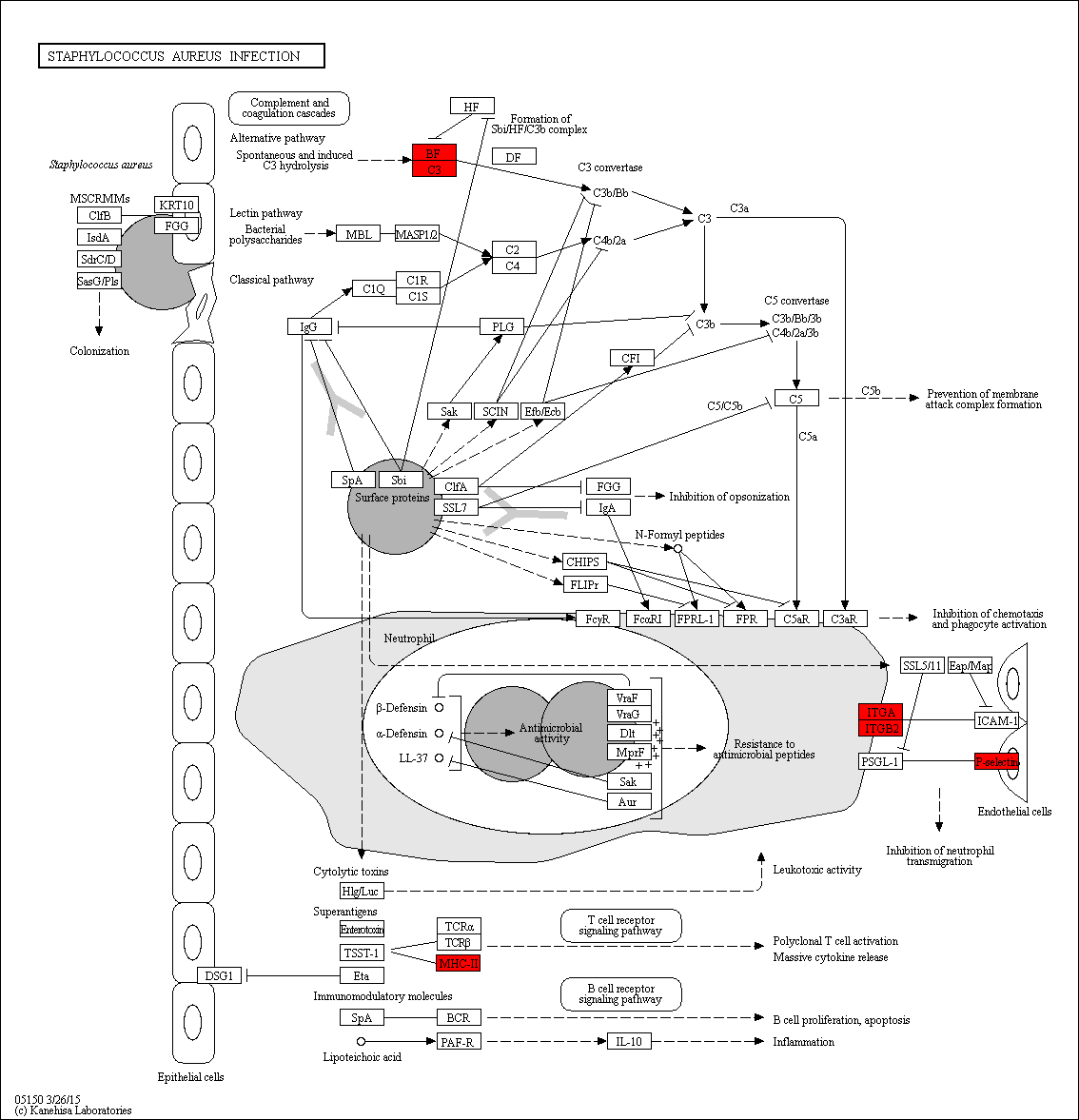

Supplement: Supplementary file 3 — Source Data [file 41467_2021_24610_MOESM3_ESM.zip › sounce data/RNAseq/path/CM0-VS-KOMO_ko05150.png]

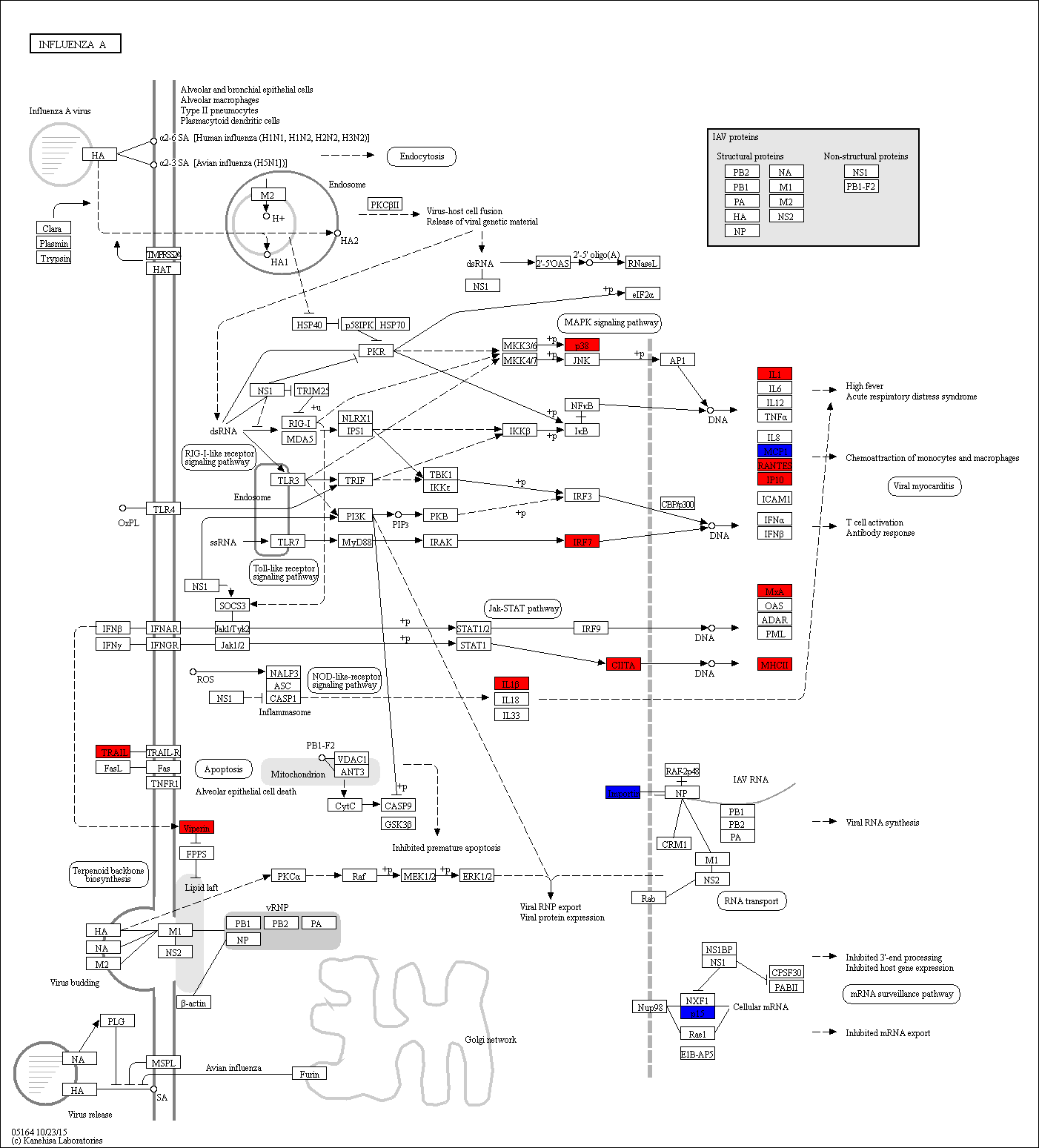

Supplement: Supplementary file 3 — Source Data [file 41467_2021_24610_MOESM3_ESM.zip › sounce data/RNAseq/path/CM0-VS-KOMO_ko05164.png]

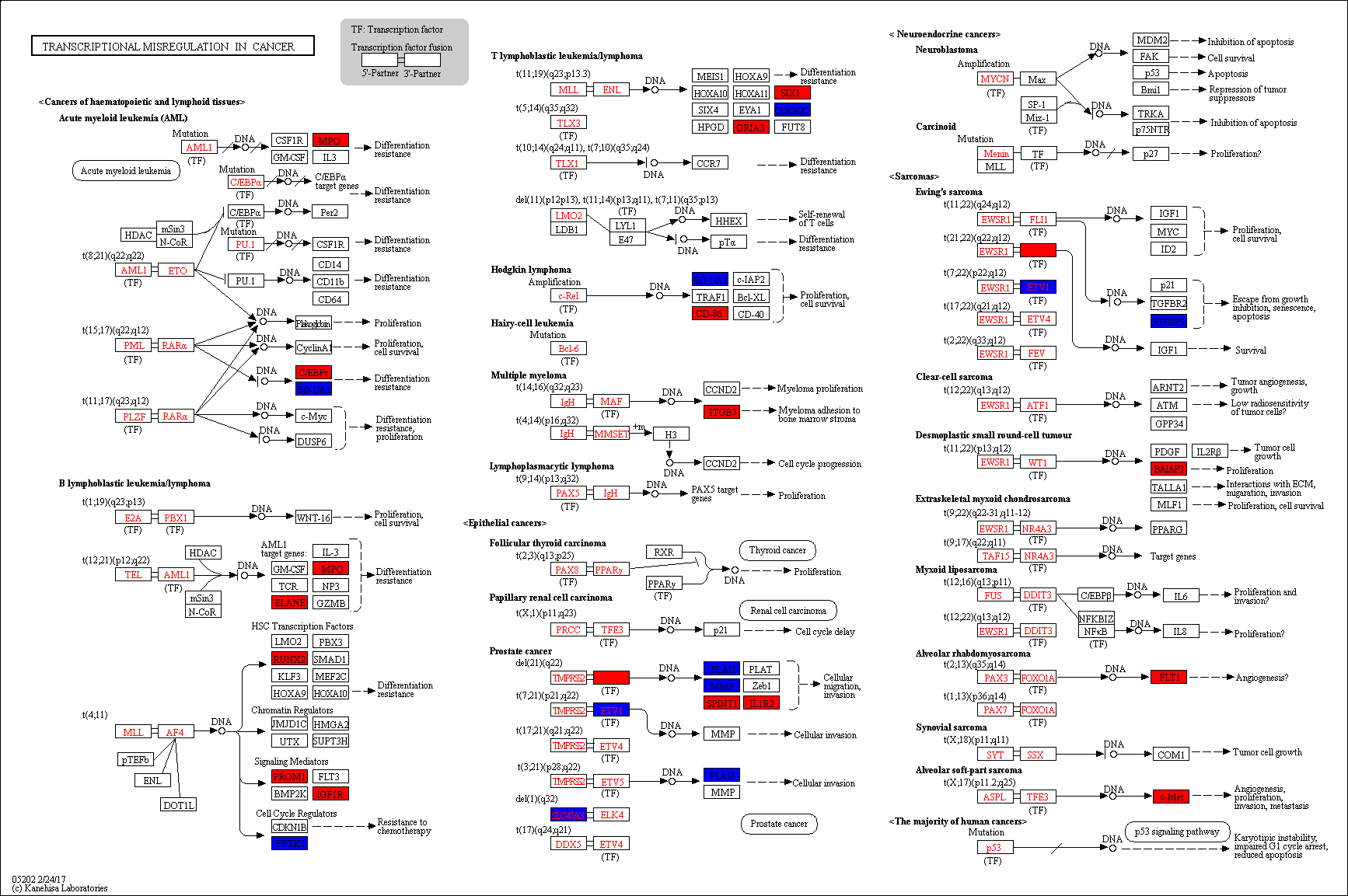

Supplement: Supplementary file 3 — Source Data [file 41467_2021_24610_MOESM3_ESM.zip › sounce data/RNAseq/path/CM0-VS-KOMO_ko05202.png]

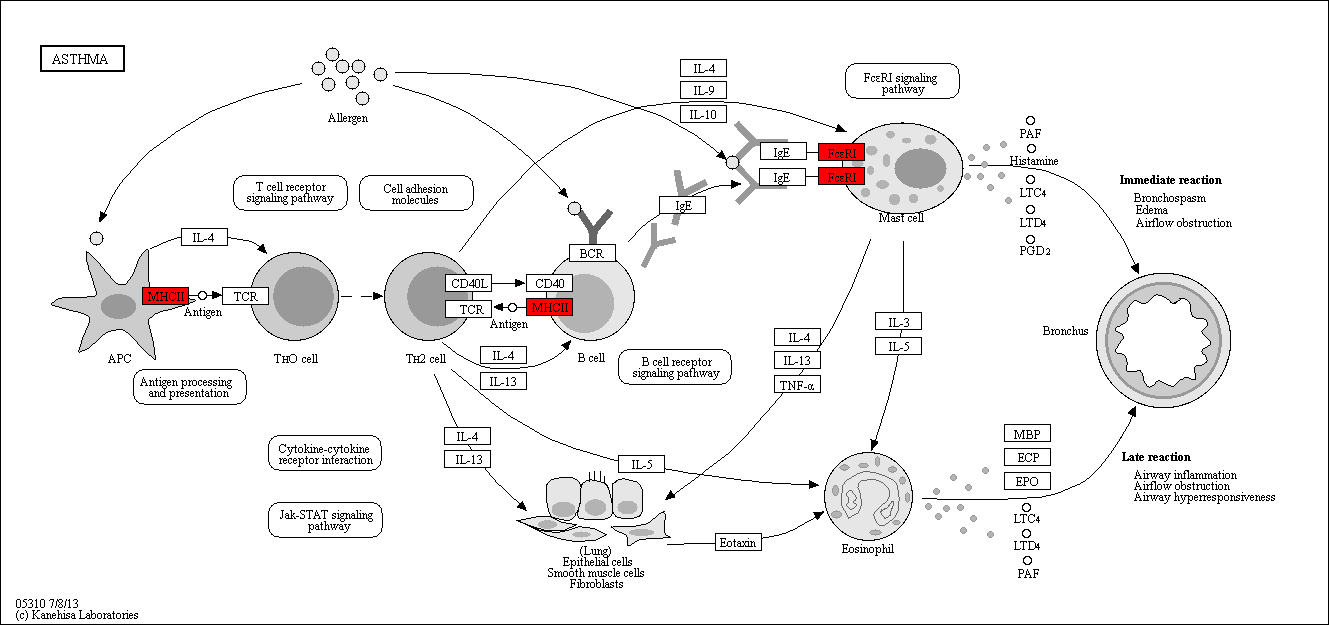

Supplement: Supplementary file 3 — Source Data [file 41467_2021_24610_MOESM3_ESM.zip › sounce data/RNAseq/path/CM0-VS-KOMO_ko05310.png]

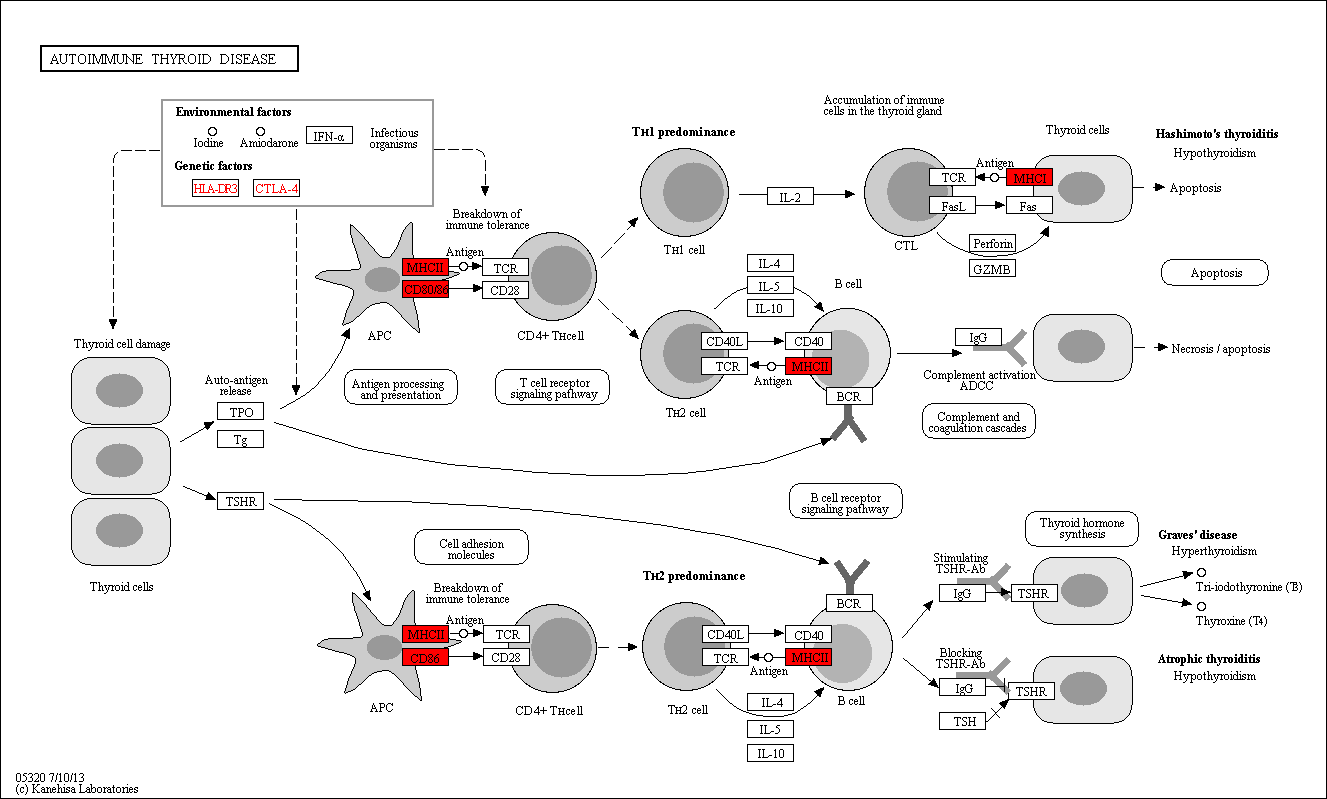

Supplement: Supplementary file 3 — Source Data [file 41467_2021_24610_MOESM3_ESM.zip › sounce data/RNAseq/path/CM0-VS-KOMO_ko05320.png]

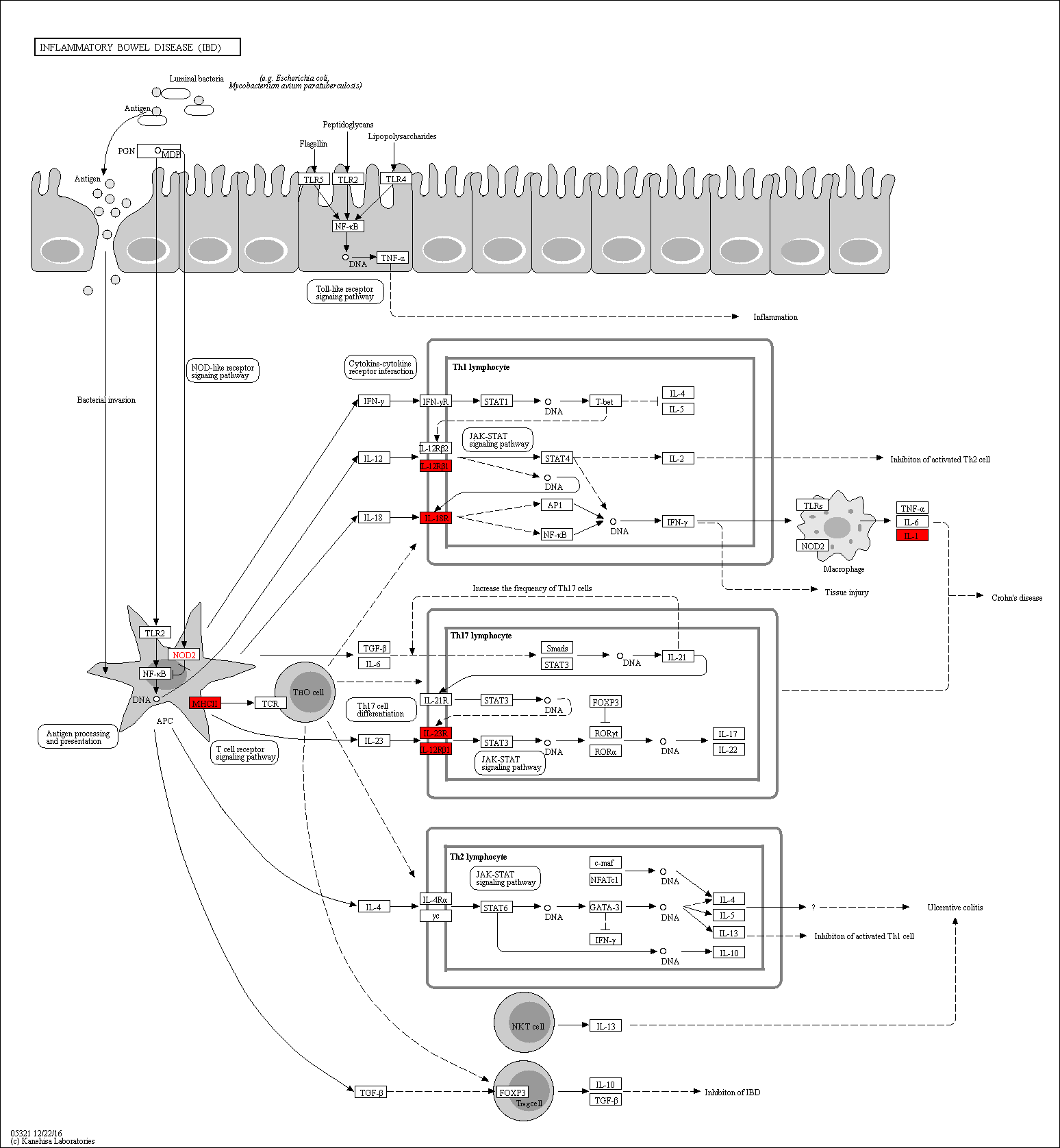

Supplement: Supplementary file 3 — Source Data [file 41467_2021_24610_MOESM3_ESM.zip › sounce data/RNAseq/path/CM0-VS-KOMO_ko05321.png]

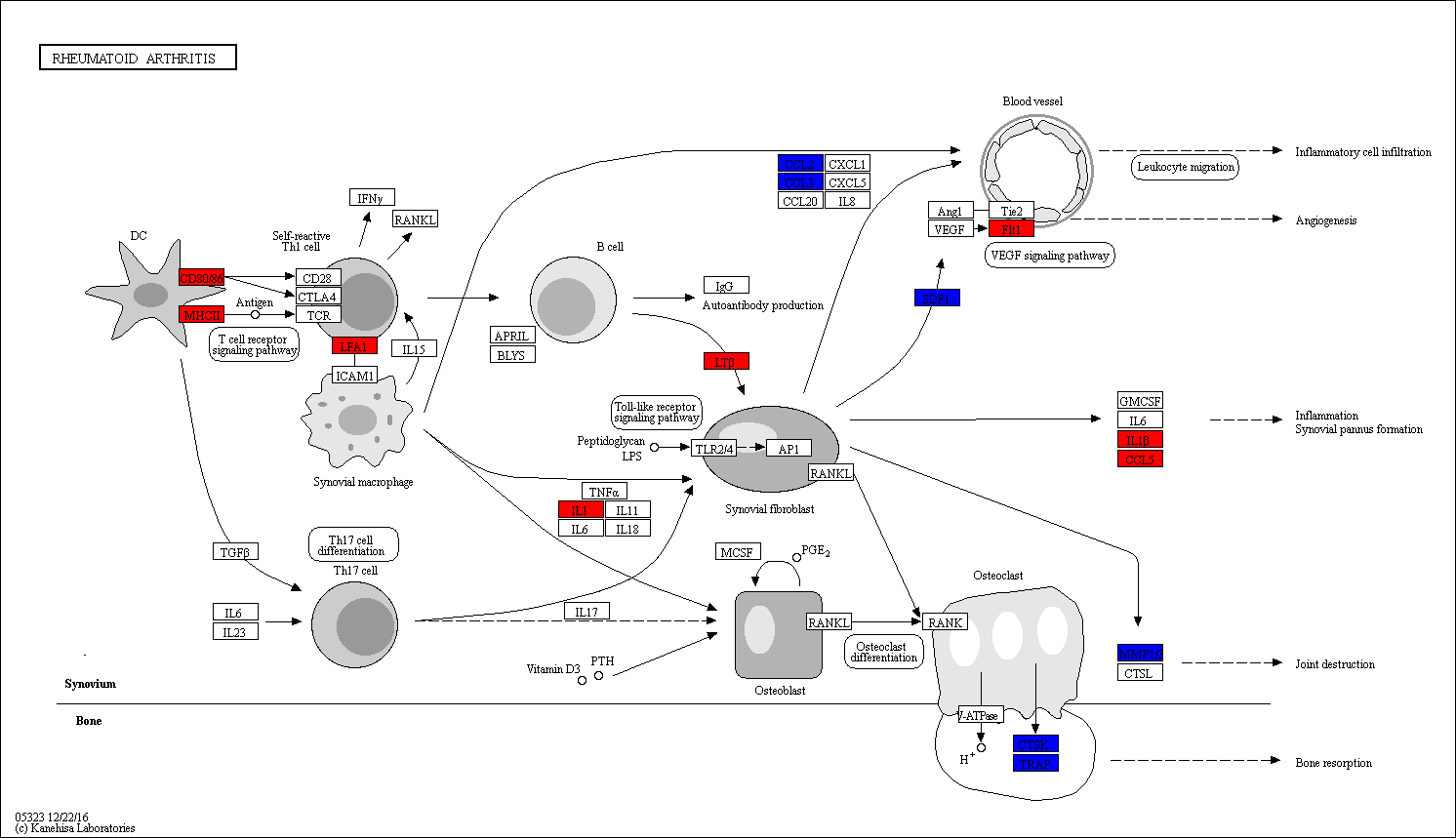

Supplement: Supplementary file 3 — Source Data [file 41467_2021_24610_MOESM3_ESM.zip › sounce data/RNAseq/path/CM0-VS-KOMO_ko05323.png]

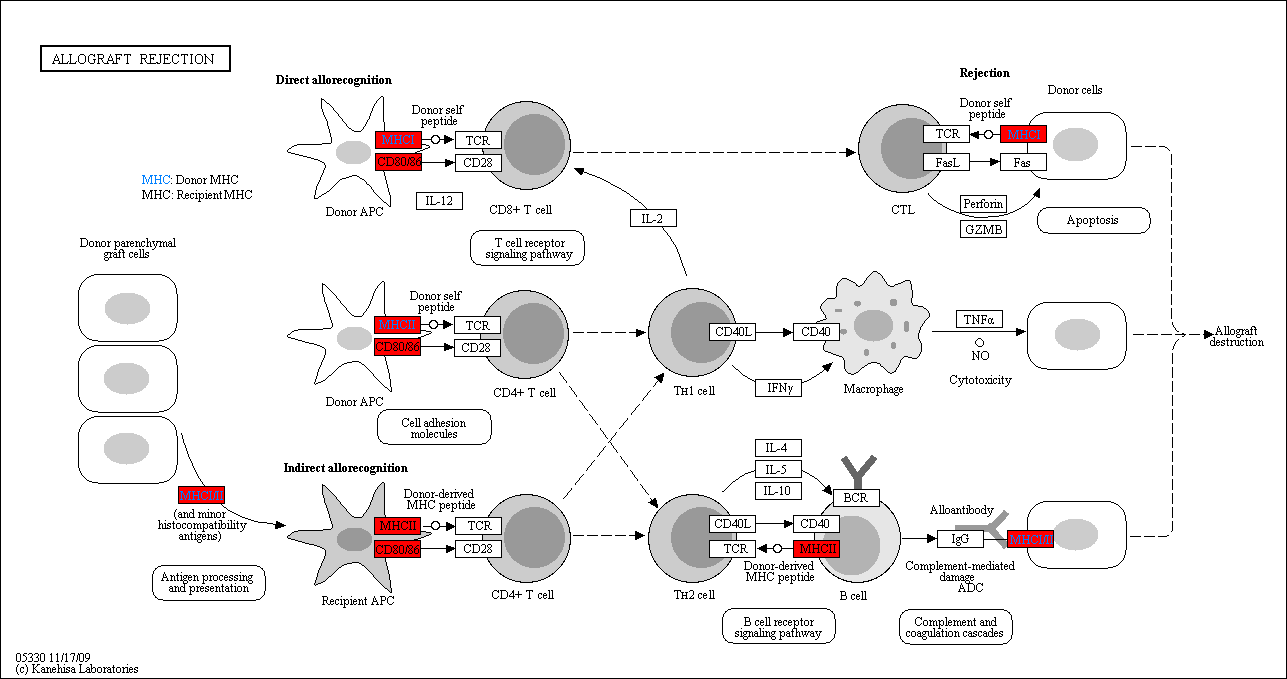

Supplement: Supplementary file 3 — Source Data [file 41467_2021_24610_MOESM3_ESM.zip › sounce data/RNAseq/path/CM0-VS-KOMO_ko05330.png]

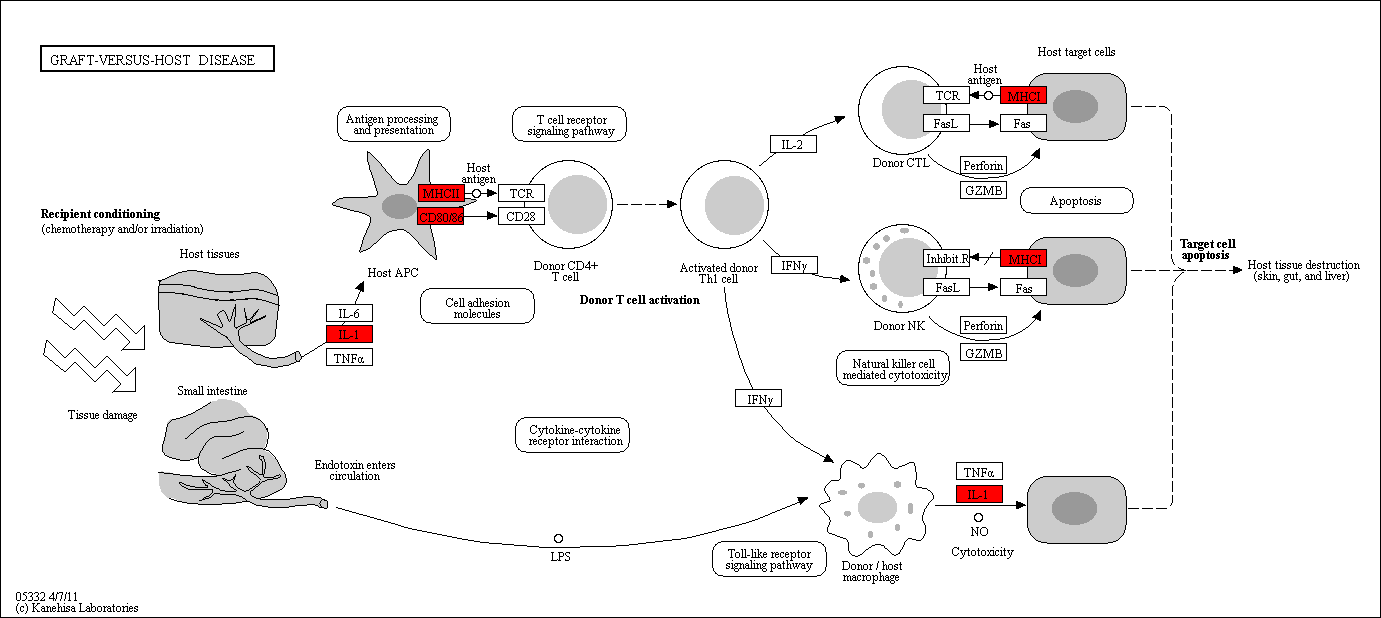

Supplement: Supplementary file 3 — Source Data [file 41467_2021_24610_MOESM3_ESM.zip › sounce data/RNAseq/path/CM0-VS-KOMO_ko05332.png]

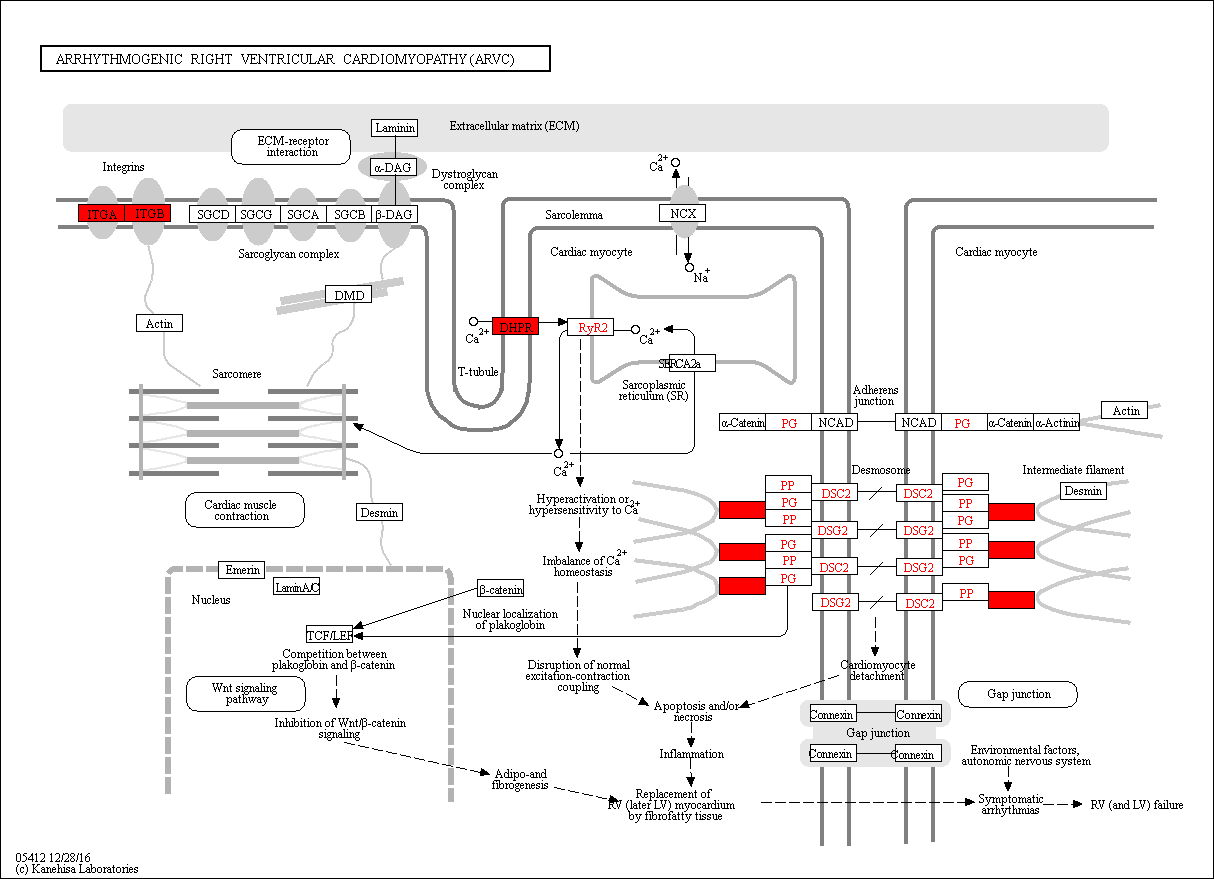

Supplement: Supplementary file 3 — Source Data [file 41467_2021_24610_MOESM3_ESM.zip › sounce data/RNAseq/path/CM0-VS-KOMO_ko05412.png]

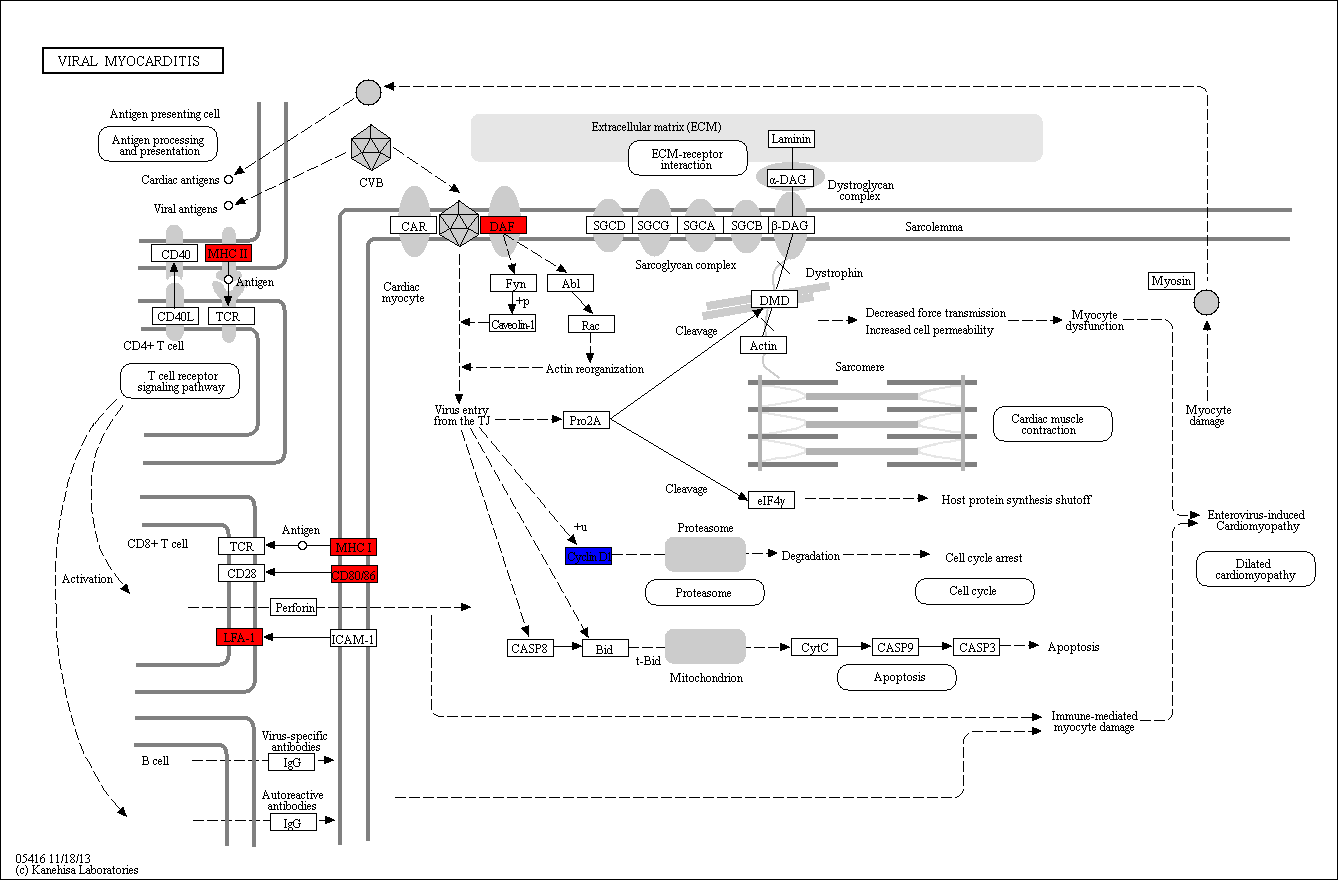

Supplement: Supplementary file 3 — Source Data [file 41467_2021_24610_MOESM3_ESM.zip › sounce data/RNAseq/path/CM0-VS-KOMO_ko05416.png]

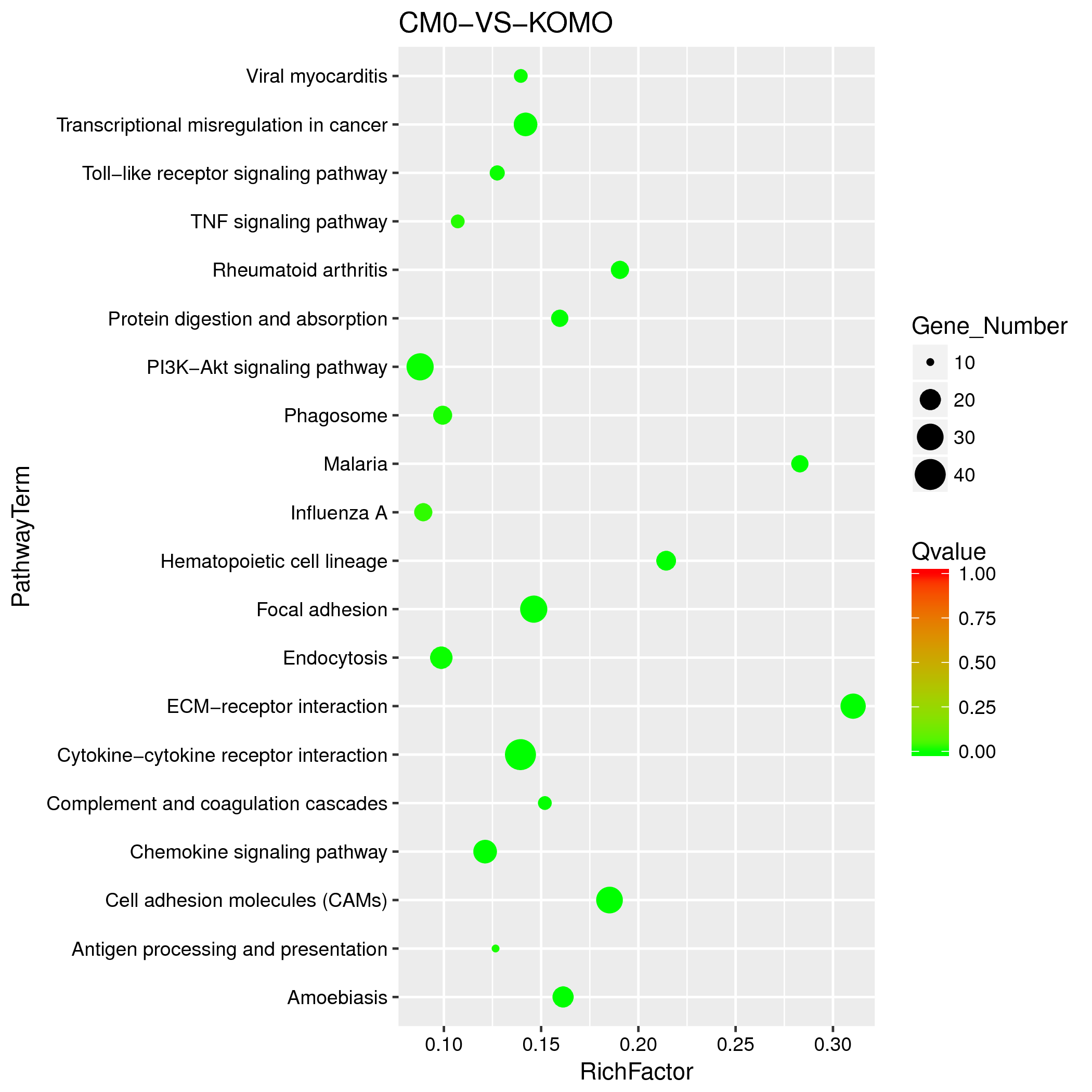

Supplement: Supplementary file 3 — Source Data [file 41467_2021_24610_MOESM3_ESM.zip › sounce data/RNAseq/path way/CM0-VS-KOMO_rich.png]
